# Nicotinic acid improves mitochondrial function and associated transcriptional pathways in older inactive males

**DOI:** 10.1515/teb-2024-0030

**Published:** 2024-11-25

**Authors:** Colleen S. Deane, Craig R. G. Willis, Iain J. Gallagher, Matthew S. Brook, Nima Gharahdaghi, Lee J. Wylie, Daniel J. Wilkinson, Kenneth Smith, Philip J. Atherton, Timothy Etheridge

**Affiliations:** Faculty of Health and Life Sciences, 3286 University of Exeter, Exeter, UK; Human Development & Health, Faculty of Medicine, University of Southampton, Southampton General Hospital, Southampton, UK; School of Chemistry and Biosciences, Faculty of Life Sciences, University of Bradford, Bradford, UK; Centre for Biomedicine & Global Health, Edinburgh Napier University, Edinburgh, UK; MRC-Versus Arthritis Centre for Musculoskeletal Ageing Research and National Institute for Health Research Nottingham Biomedical Research Centre, School of Medicine, University of Nottingham, Derby, UK; School of Life Sciences, University of Nottingham, Nottingham, UK

**Keywords:** acipimox, NAD^+^, nicotinic acid, mitochondria, ageing, skeletal muscle

## Abstract

**Objectives:**

To examine the effect of the NAD^+^ precursor, nicotinic acid (NA), for improving skeletal muscle status in sedentary older people.

**Methods:**

In a double-blind, randomised, placebo-controlled design, 18 sedentary yet otherwise healthy older (65–75 y) males were assigned to 2-weeks of NA (acipimox; 250 mg × 3 daily, n=8) or placebo (PLA, n=10) supplementation. At baseline, and after week 1 and week 2 of supplementation, a battery of functional, metabolic, and molecular readouts were measured.

**Results:**

Resting and submaximal respiratory exchange ratio was lower (p<0.05) after 2 weeks in the NA group only, but maximal aerobic and anaerobic function and glucose handling were unchanged (p>0.05). Bayesian statistical modelling identified that leak, maximal coupled and maximal uncoupled mitochondrial respiratory states, increased over the 2-week supplemental period in the NA group (probability for a positive change (pd) 85.2, 90.8 and 95.9 %, respectively) but not in PLA. Citrate synthase and protein content of complex II (SDHB) and V (ATP5A) electron transport chain (ETC) components increased over the 2-week period in the NA group only (pd 95.1, 74.5 and 82.3 %, respectively). Mitochondrial and myofibrillar protein synthetic rates remained unchanged in both groups. NA intake altered the muscle transcriptome by increasing the expression of gene pathways related to cell adhesion/cytoskeleton organisation and inflammation/immunity and decreasing pathway expression of ETC and aerobic respiration processes. NAD^+^-specific pathways (e.g., *de novo* NAD^+^ biosynthetic processes) and genes (e.g., *NADSYN1*) were uniquely regulated by NA.

**Conclusions:**

NA might be an effective strategy for improving ageing muscle mitochondrial health.

## Introduction

By 2050 the World’s population of older adults (>60 years) is projected to more than double from one billion at present to 2.1 billion. A defining feature of ageing is the loss of muscle mass (sarcopenia, ∼1 % per year) and functional strength (dynapenia, 2–4 % per year) [1], which is prevalent in up to 16 % of ≥60-year old’s [2]. Since skeletal muscles are integral for whole-body metabolism and locomotion, sarcopenia/dynapenia are key risk factors of frailty-related falls [3], morbidity [4] and mortality [5]. Combined with an estimated £2.5 billion annual healthcare cost attributed to muscle weakness [6], effective interventions against sarcopenia/dynapenia will have significant socio-economic implications.

A consistent feature in the aetiology of sarcopenia/dynapenia is declining mitochondrial health [7]. For example, ageing muscle displays reduced mitochondrial volume [8], with the remaining mitochondria displaying reduced oxidative enzyme activity [8, 9]. These age-related mitochondrial changes translate to impaired respiratory capacity, maximal ATP production [10, 11] and lowered fatty acid β-oxidation with subsequent glucose intolerance [12]. Thus, when mitochondria become dysfunctional the net effect is an energy homeostatic imbalance sufficient to compromise muscle quality, metabolic health and capacity to perform even low-intensity activities of daily living [13]. Targeting mitochondrial dysfunction, therefore, represents an attractive therapeutic target for countering age-related muscle decline.

Mitochondrial oxidative phosphorylation-mediated ATP synthesis requires shuttling of protons between nicotinamide adenine dinucleotide (NAD^+^) and its reduced form NADH, and NAD^+^ is a rate-limiting co-substrate for the sirtuin enzymes to regulate oxidative metabolism [14]. Additionally, substrate level phosphorylation-based ATP resynthesis requires NAD^+^ during the anaerobic conversion of glucose molecules into pyruvate, NADH and ATP [15]. Thus, age-related changes in muscle NAD^+^ homeostasis could negatively affect oxidative and non-oxidative ATP generation and, therefore, endurance and power-based performance, respectively. Indeed, older inactive and sarcopenic muscle displays downregulation of NAD^+^ content and NAD^+^ networks 16], [17], [18, possibly linked to declines in the NAD^+^ biosynthesis rate-limiting enzyme, nicotinamide phosphoribosyltransferase (NAMPT) and/or increases in the nuclear DNA repair catalyst poly-ADP-ribose polymerase, an NAD^+^ consuming enzyme whose activity is upregulated in ageing muscle [19]. These lower levels of NAD^+^ correlate with reduced measures of muscle and mitochondrial function, including grip strength and complex I activity [17, 18]. On this basis, augmenting NAD^+^ levels might rejuvenate mitochondria dysfunction-related muscle decline during ageing.

Several dietary precursors of NAD^+^ target distinct metabolic pathways to promote *de novo* NAD^+^ synthesis. For example, nicotinamide riboside (NR) involves a unique two-step process to form NAD^+^ via the salvage pathway [20]. Conversely, alternate precursors such as nicotinic acid (NA) are independently converted to NAD^+^ through the three-step Preiss-Handler pathway [20]. Pre-clinical evidence suggests muscle NAD^+^ levels can be restored irrespective of the upstream precursor (NA, NR or nicotinamide mononucleotide) [21, 22] which, in turn, improves mitochondrial function, substrate utilisation and muscle mass in ageing and diabetic animals 21], [22], [23], [24], [25. Exogenous regulation of NAD^+^ metabolism in humans, however, appears more complex. Short-medium term NR supplementation consistently fails to improve mitochondrial function and/or aerobic performance parameters in young exercising [26, 27], overweight or obese [28], obese insulin resistant [29] or older [27, 30] people with varying habitual activity levels, despite increasing NAD^+^, markers of NAD^+^ synthesis, and the associated metabolome–indicative of some level of bioavailability [27, 28, 30, 31]. While longer-term NR supplementation has been shown to improve mitochondrial biogenesis, it cannot be discounted that this is a compensatory mechanism for NR’s toxicity to mitochondria [32]. In contrast, NA ingestion increases mitochondrial mass and cytochrome *c* oxidase activity in healthy adults and mitochondrial myopathy patients [33]. The NA slow release donor (acipimox) also improves insulin sensitivity [34] and increases mitochondrial respiration in type II diabetics [35, 36]. As such, NA might confer specific advantages for NAD^+^-based mitochondrial therapies, but this has not yet been examined in ageing human muscle.

**Figure 1:**
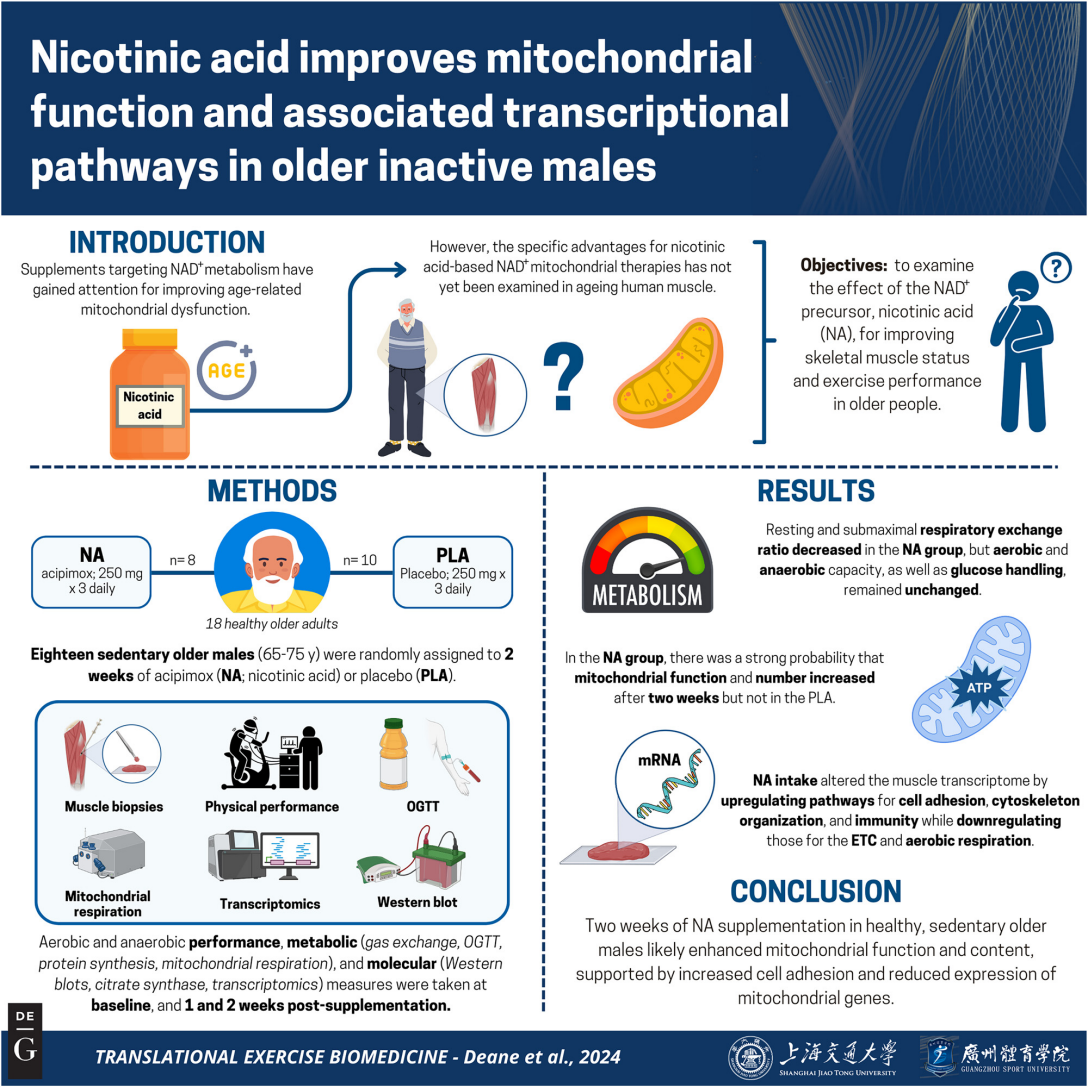
Graphical representation of this study. Key points: (1) NA decreased resting and submaximal RER but did not alter (an)aerobic capacity, (2) there was a strong probability NA increased mitochondrial number and function, and (3) NA intake altered the muscle transcriptome by upregulating genes related to cell adhesion, cytoskeleton organization and immunity, and downregulating genes related to the ETC and aerobic respiration. ETC, electron transport chain; NA, nicotinic acid; OGTT, oral glucose tolerance test; RER, respiratory exchange ratio. Figure created with BioRender.

This study, therefore, utilised a systems biological approach to examine the efficacy of 2 weeks supplementation with an NA analogue (acipimox) for altering skeletal muscle performance, protein turnover, mitochondrial function and molecular/transcriptomic profiles in physically inactive older people. The summary of this article is presented in Figure 1.

## Methods

### Study design and ethics

**Figure 2:**
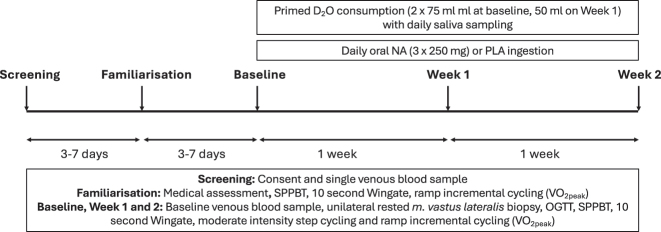
Study schematic. D_2_O, deuterium oxide; NA, nicotinic acid; OGTT, oral glucose tolerance test; PLA, placebo; SPPBT, short physical performance battery test.

Twenty healthy older (65–75 years) physically inactive males were recruited for this randomised, double-blind, placebo-controlled trial. Of these, 18 completed the study (Table 1) (see Deane et al.*,* [37] for comprehensive study recruitment details). During screening, the risks and procedures of the study were explained, after which volunteers provided written informed consent and a venous blood sample was obtained, which was screened for routine blood biochemistry (Table 1). Volunteers were instructed to refrain from exercise and analgesics throughout the duration of the study and to avoid alcohol consumption 72 h before all experimental study visits. The study design is illustrated in Figure 2 and was approved by the South West Frenchay Research Ethics Committee (NHS ethics) (16/SW/0,099) and conducted according to the Declaration of Helsinki (clinical trial registration #NCT02792621).

**Table 1:** Volunteer baseline characteristics.

Characteristic	NA	PLA	p-Value
Age, years	69.1 ± 1.0	69.4 ± 0.8	p=0.84
Height, m	1.78 ± 0.03	1.75 ± 0.01	p=0.41
Weight, kg	80.1 ± 3.2	79.5 ± 1.4	p=0.84
BMI, kg/m^2^	25.4 ± 1.1	25.9 ± 0.6	p=0.71
Resting heart rate, bpm	62 ± 2	58 ± 2	p=0.22
Systolic blood pressure, mm Hg	142 ± 5	134 ± 5	p=0.30
Diastolic blood pressure, mm Hg	79 ± 3	79 ± 4	p=0.98
Fasting plasma glucose, mmol/L	5.08 ± 0.1	5.02 ± 0.1	p=0.72
HbA_1c_, mmol/mmol	35.8 ± 0.7	36.9 ± 0.7	p=0.26
Total cholesterol, mmol/L	5.2 ± 0.4	5.6 ± 0.2	p=0.41
HDL cholesterol, mmol/L	1.4 ± 0.1	1.5 ± 0.1	p=0.64
LDL cholesterol, mmol/L	3.3 ± 0.3	3.4 ± 0.4	p=0.81
Triglycerides, mmol/L	1.5 ± 0.3	1.2 ± 0.2	p=0.57

Data are mean ± S.E.M. comparisons between NA (n=8) and PLA (n=10) made by unpaired *t*-tests. BMI, body mass index; HbA_1c_, glycated haemoglobin; HDL, high-density lipoproteins; LDL, low-density lipoproteins; NA, nicotinic acid; PLA, placebo.

Volunteers returned 3–7 days after screening for a medical examination and familiarisation of the study procedures, which included height, weight, resting heart rate and blood pressure measurements, and a resting electrocardiogram (ECG). Volunteers were excluded if they were: physically active (defined herein as taking part in any planned physical activity), had a body mass index <19 or >29 kg/m^2^, taking chronic medication known to affect muscle metabolism such as nonsteroidal anti-inflammatory drugs, statins, paracetamol or aspirin or had the following: active cardiovascular, respiratory or metabolic disease, renal impairment, musculoskeletal injury, a peptic ulcer, hypersensitivity to acipimox and/ or vertigo. Following medical clearance, volunteers were familiarised with the following functional assessments; short physical performance battery test (SPPBT) to measure lower body mobility, 10 s cycling Wingate to measure anaerobic power and an incremental cycle ramp test to measure maximal oxygen uptake (VO_2peak_) (Supplementary Methods). Gas exchange and ventilatory data collected during the incremental ramp test were used to determine the gas exchange threshold using the V-slope method (Figure S1) [38]. Upon conclusion of the familiarisation visit, volunteers were randomised to either acipimox (NA) or placebo (PLA) conditions. A computer-generated randomisation log was generated by a statistician in liaison with the Exeter Clinical Trials Pharmacy Unit and was securely stored by a non-research team member of the Clinical Research Facility support team, ensuring a randomised, double-blind, placebo-controlled design. All randomisation and blinding procedures were performed by the Exeter Clinical Research Facility in accordance with their standard operating procedures.

Volunteers arrived 3–7 days after familiarisation for the first experimental study visit (Baseline) in an overnight fasted state. Upon arrival, weight, blood pressure, heart rate and ECG were recorded, a cannula was inserted into the antecubital vein and a baseline blood sample was collected. A resting muscle biopsy was obtained from the *m. vastus lateralis* of the dominant leg*,* and a 2 h oral glucose tolerance test (OGTT) (Polycal Liquid, Nutricia, Netherlands) commenced, with blood samples taken 15, 30, 45, 60, 90 and 120 min following the consumption of 113 mL Polycal (Polycal Liquid, Nutricia, Netherlands), equivalent to 75 g glucose. Following completion of the OGTT, volunteers provided a baseline saliva sample for measurement of body water and then performed the SPPBT, Wingate, moderate intensity step and ramp incremental test (Supplementary Methods). Afterwards, volunteers consumed the first dose of a two-week supplemental period (described below). To obtain free-living measures of muscle protein synthesis [39], volunteers consumed 150 mL deuterium oxide (D_2_O), provided in 2 × 75 mL doses separated by 30 min, and thereafter provided daily saliva samples. Volunteers wore a FitBit One (FitBit Inc, San Francisco, USA) for the duration of the two-week intervention to measure daily step count. Dietary intake was monitored via a 4-day diet diary and was analysed using Nutritics software (Nutritics Ltd, Dublin, Ireland).

After baseline testing, volunteers returned to the laboratory after one week (week 1) and two weeks (week 2) supplementation. Volunteers arrived fasted and had not taken any supplements on the morning of testing to avoid studying the acute effects of NA [36]. Procedures during week 1 and week 2 experimental visits were identical to the Baseline visit, except the volume of D_2_O consumed was reduced to 50 mL (Figure 2).

### Supplementation protocol

NA activates the G protein-coupled receptor GPR109A, sometimes causing skin flushing side-effects. Clinical applications of oral NA have, therefore, been limited [40]. Consequently, synthetic and sustained-release NA formulations have been developed, including acipimox, a clinically available drug usually used to treat hyperlipidaemia [41]. Compared with naturally occurring NA, acipimox is associated with fewer incidences of side effects including flushing, is well tolerated by patients [42], and has been shown to improve mitochondrial function in diabetic humans [36]. Thus, acipimox was the chosen NA supplement for this study. The acipimox protocol matched dosing regimens that augment mitochondrial function in diabetic patients [36]: 3 × 250 mg capsules per day, consumed morning, noon and evening, over a two-week supplementation period (PLA was cellulose microcrystalline, 3 × 250 mg per day). Crucially, on study visits, supplements (NA or PLA) were not consumed until after all testing was complete so that the prolonged treatment effect of NA was studied, *not* the acute effect of the last dose taken. Side-effect incidences and supplement adherence were monitored throughout via supplement diaries and the return of supplement bottles to pharmacy for counting.

### Muscle biopsies

Muscle biopsies were obtained from the mid-portion of the *m. vastus lateralis* using the Bergström biopsy technique, taken under local anaesthetic using 2 % lidocaine (Hameln Pharmaceuticals Ltd, Gloucester, UK) under sterile techniques. For high resolution respirometry (HRR) analysis, a portion of the muscle sample (∼20 mg) was immediately submerged in ice-cold biopsy preservation solution (BIOPS, pH 7.1) containing 10 mM Ca-EGTA buffer, 0.1 μM free Ca^2+^, 20 mM imidazole, 20 mM taurine, 50 mM K-MES, 0.5 mM DTT, 6.56 mM MgCl_2_, 5.77 mM ATP, 15 mM phosphocreatine, which remained on ice until analysis. Although mitochondrial respiration is stable for up to 24 h in BIOPS [29], processing and analysis of muscle samples commenced <6 h after collection. The remaining muscle biopsy sample was rapidly dissected free of visible fat and connective tissue, washed in ice-cold phosphate buffered saline (PBS), blotted on gauze and frozen in liquid nitrogen, and stored at −80 °C until subsequent metabolic and molecular analysis.

### Preparation and permeabilisation of muscle fibres for HRR

Muscle fibre bundles preserved in BIOPS for HRR were mechanically separated in ice-cold BIOPS solution on a petri dish using sharp dissecting needles under a dissecting microscope (Motic SMZ-161, Motic, Hong Kong, Asia). All visible fat and connective tissue were removed, and fibres were incubated in single wells containing 1 mL of BIOPS, in duplicate. To permeabilise the sarcolemmal membrane, fibre bundles were transferred into 1 mL of freshly prepared saponin solution (50 μg/mL BIOPS) and incubated at 4 °C for 30 min with intermittent manual agitation. To remove excess saponin, fibre bundles were incubated in mitochondrial respiration medium (MiR05, pH 7.1) containing 0.5 mM EGTA, 3 mM MgCl_2_, 60 mM lactobionic acid, 20 mM taurine, 10 mM KH_2_PO_4_, 20 mM HEPES, 110 mM d-sucrose, 1 g/L fatty acid free bovine serum albumin, at 4 °C for 10 min. Approximately 2–4 mg of permeabilised myofibres were blotted and weighed (XP205 Delta Range, Mettler Toledo, Ohio, USA) and transferred in ice-cold MiR05 to the Oxygraph-2K (O2K) respirometer chamber (Oroboros Instruments, Innsbruck, Austria) containing 2 mL of MiR05 medium.

### HRR and substrate uncoupler inhibitor titration protocol

Prior to each run, a background calibration was conducted for each polygraphic oxygen sensor (POS) to ensure the POS was acceptable. Once myofibre bundles were added to the chambers, ∼3 mL of 100 % O_2_ was added to each chamber to achieve an O_2_ concentration ∼400 μM. In order to minimise O_2_ dependency artefacts in the data, O_2_ flux measurements were made when the O_2_ concentration was between 200 and 400 μM. The substrate uncoupler inhibitor titration (SUIT) protocol used has been described previously [43]. Leak respiration (Li) was achieved through the simultaneous addition of 5 mM pyruvate, 2 mM malate and 10 mM glutamate to stimulate electron flow through mitochondrial complex I. The addition of 5 mM ADP coupled electron transfer to phosphorylation (P_i_). In order to induce maximal phosphorylating state 3 respiration with electron input from complex I and II, 10 mM succinate was injected (P_i+ii_). Integrity of the mitochondrial membranes was assessed via the addition of 10 μM cytochrome *c* (<10 % change in O_2_ flux indicated intact membranes). Chambers were re-oxygenated prior to uncoupling oxidative phosphorylation by titrating 1 μL of 1 mM FCCP (E). Finally, 12 μM Antimycin A was added to inhibit mitochondrial complex III. For each sample, the protocol was simultaneously performed in duplicate using both chambers. Following the first injection (i.e. substrates), ∼5–10 min elapsed before injecting the subsequent substrate/ inhibitor/ uncoupler, ensuring at least 2 min of steady state O_2_ flux data was achieved for data analysis [43]. For FCCP, peak O_2_ flux data was used since FCCP initially causes a peak, followed by a decline in respiration [43].

### Citrate synthase activity

Citrate synthase (CS) activity was measured as previously described [44]. Approximately 3–5 mg of muscle was mechanically homogenised in 1 % Triton X-100 buffer following centrifugation at 22,000*g* for 3 min at 4 °C and the resultant supernatant transferred to a fresh ice-cold Eppendorf. Thereafter, 300 μL of CS master mix (containing 28 % 0.05 M Tris buffer (pH 7.6), 1.3 % 1 mM 5,5′-dithiobis-2-nitrobenzoic acid, 7 % acetyl-coenzyme A (1.36 mg mL^−1^), 0.08 % oxaloacetate (9.88 mg mL^−1^) and 63 % ddH_2_O) was added to 96-plate wells and incubated at 25 °C for 5 min, prior to being measured at 412 nm as a blank. Finally, 20 μL of sample supernatant was used to measure the V_max_, defined as the maximum rate of reaction [44].

### Immunoblotting electron transport chain components

Immunoblotting was performed on mitochondria-containing sarcoplasmic fractions extracted from ∼10 mg of frozen muscle tissue, with protein concentrations determined by spectrophotometry. Samples were standardised to 1 μg/μL by dilution with 3× Laemmli loading buffer and heated at 40° for 4 min. Precisely 5 μL of proteins were loaded onto Criterion XT Bis-Tris-12 % SDS-PAGE gels (Biorad, Hemel Hempstead, UK) for electrophoresis at 185 V for 50 min. Samples were transferred to methanol-soaked polyvinylidene difluoride membranes at 100 V for 45 min. Membranes were blocked in 2.5 % low-fat milk (diluted in Tris-buffered saline and 0.1 % Tween-20 (TBS-T)) for 1 h at room temperature and then incubated in total oxidative phosphorylation (OXPHOS, Abcam, Cambridge, MA, USA, ab110411) primary antibody (1:10,000 dilution in 2.5 % bovine serum albumin in TBS-T) for 4 h at 4 °C. Thereafter, membranes were washed 3 × 4 min in TBS-T and incubated in horseradish peroxidase (HRP)-conjugated secondary antibody (New England Biolabs; 1:2,000 in 2.5 % milk in TBS-T) for 1 h at room temperature. Membranes were washed in TBS-T (3 × 4 min) and subsequently submerged in Chemiluminescent HRP substrate (Millipore Corporation, Billerica, MA, USA) and imaged on a Chemidoc MP (Bio-Rad Laboratories, Hertforshire, UK). Protein bands were quantified by densitometry (ensuring no pixel saturation) and the relative arbitrary units were normalised to Coomassie stained proteins to correct for protein loading [30].

### Body water and protein bound alanine muscle fraction enrichment

Body water and muscle protein enrichment were measured as previously described [25, 31]. Briefly, after collection, saliva was centrifuged at 13,300 rpm for 10 min at 4 °C, aliquoted and stored at −20 °C until further processing. Saliva samples were defrosted and 100 μL was pipetted into the cap of an inverted auto-sampler vial, placed inverted on a heating block for 4 h at 95 °C and then rapidly cooled on ice for 10 min. The remaining water distillate was transferred into a vial insert within a new auto-sampler vial. Body water (0.1 μL) was injected into a high-temperature conversion elemental analyzer (Thermo Finnigan, Thermo Scientific, Hemel Hempstead, UK) connected to an isotope ratio mass spectrometer (Delta V Advantage, Thermo Scientific).

For the isolation of myofibrillar and mitochondrial protein, ∼100 mg of muscle was homogenised in ice-cold buffer followed by rotation for 10 min and centrifugation for 15 min at 11,000*g* at 4 °C. The resultant pellet was Dounce homogenised in 500 μL mitochondrial extraction buffer (MEB) and centrifuged at 1,000*g* for 5 min 4 °C, generating a myofibrillar/collagen pellet which was stored at −80 °C until further processing. The mitochondrial-containing supernatant was centrifuged twice at 11,000*g* for 15 min at 4 °C, separated by a MEB wash, and the mitochondrial pellet was stored at −80 °C until further processing. The myofibrillar-containing pellet was solubilised in 750 μL of 0.3 M NaOH and separated from the insoluble collagen fraction via centrifugation. Myofibrillar proteins were precipitated using 1 mL of 1 M perchloric acid and pelleted via centrifugation. Protein-bound amino acids derived from the myofibrillar and mitochondrial pellets were released using acid hydrolysis by incubating at 110 °C in 0.1 M HCl in a Dowex H+ resin slurry overnight followed by elution with 2 M NH_4_OH and subsequent drying down. Dried AAs were derivatised by suspension in 60 μL distilled water and 32 μL methanol, vortexing, and then the addition of 10 μL pyridine and 8 μL of methylchloroformate. The AA *n-*methoxycarbonyl methyl esters were extracted into 100 μL chloroform and a molecular sieve was added for ∼20 s to remove water before transferring the sample into a clean glass Gas Chromatography insert. The incorporation of deuterium into protein-bound alanine was determined by gas-chomotography-pyrolysis-isotope ratio mass spectrometry (Delta V Advantage, Thermo, Hemel Hempstead, UK) [29, 31].

### Calculation of mitochondrial and myofibrillar fractional synthesis rate

Muscle protein synthesis was calculated from the deuterium enrichment (APE) in alanine in myofibrillar and mitochondrial proteins using body water enrichment (APE, corrected for the mean number of deuterium moieties incorporated per alanine, 3.7, and the dilution from the total number of hydrogens in the derivative, i.e. 11) as the precursor labelling between subsequent biopsies [29]. Fractional synthesis rate was calculated as:

$$\text{FSR }\left({\%\;\text{day}}^{-1}\right)=-\text{In}\left[\frac{1-\left[\frac{{\text{APE}}_{\text{Ala}}}{{\text{APE}}_{P}}\right]}{t}\right]$$

### Generation and pre-processing of RNA-seq data

Extraction, library preparation and sequencing of RNA from muscle samples was performed by the Beijing Genomics Institute (BGI), with 100 bp paired-end reads generated in a strand-specific manner (second strand cDNA synthesis with dUTP) using the DNBSEQ platform. Raw reads were cleaned of adaptor sequences, contamination and low-quality reads, which were checked with FastQC (Babraham Bioinformatics) and deemed to be of adequate quality (median per base qualities >30 and no identifiable presence of adaptor sequences). Clean reads were subsequently pseudo-aligned using Kallisto (v0.46.1; with bias correction) [45] to estimate transcript-level abundances. In which case, the reference transcriptome was compiled based on Ensembl annotated transcript sequences, which were extracted from the Ensembl Release 107 transcript annotation file (‘Homo_sapiens.GRCh38.107.gtf.gz’) in conjunction with the associated primary assembly version of the human reference genome (‘Homo_sapiens.GRCh38.dna.primary_assembly.fa.gz’) using GFFread [46]. The tximport R package [47] was then used to infer gene-level counts from estimated transcript abundances. Violunteers with RNA-seq data available at every time-point were kept (n=1 PLA subject removed on this ground) and genes displaying consistently detectable levels of expression (count ≥10 in each remaining sample) retained, with 11,511 genes consequently remaining for downstream analyses.

### Analyses of RNA-seq data

Differential expression analysis of retained genes was first performed using the DESeq2 R package [48], with a design matrix implemented as per the developer’s recommendations for mixed-design experiments, and Wald tests used to analyse for genes differentially regulated at week 1 or week 2 per condition relative to their corresponding baselines. In each case, log2 fold-change estimates were derived via the ‘ashr’ adaptive shrinkage algorithm [49], and differentially expressed genes defined as those with a Benjamini-Hochberg False Discovery Rate (FDR)≤0.1. Genes specifically regulated by NA were consequently defined as any gene differentially expressed at week 1 and/or week 2 in the NA group that was neither differentially expressed at week 1 nor week 2 in the PLA group. Such NA-specific genes were further overlayed to deduce time-(in)dependent NA-specific gene regulation.

The functional characteristics of NA-specific gene signatures were examined by testing for enrichment of Gene Ontology (GO) Biological Process (BP) terms, as well as Reactome Pathway terms, using the gprofiler2 R package [50]. In each case, all genes utilised for differential expression analysis were input as background and the annotated statistical domain scope option employed, with enriched terms selected as those with FDR<0.05 enriched for at least two genes. To elucidate biological processes and pathways unique to NA gene signatures, the above process was also repeated for PLA-specific gene signatures (i.e., genes regulated at week 1 and/or week 2 with PLA but not at all regulated by NA) and signatures of generically regulated genes (i.e., genes regulated at week 1 and/or week to in both NA and PLA groups), with biological processes/pathways enriched for NA-specific genes then filtered to remove any that were also enriched with PLA-specific and/or generically regulated genes.

Finally, we also performed a more ‘targeted’ NAD gene analysis. In which case, we derived a core set of NAD-related pathways by searching GO BP, Reactome, KEGG and WikiPathway terms (obtained from MsigDB v7.5.1 using the msigdbr R package) for those that include ‘NAD’, ‘Nicotinate’ or ‘Nicotinamide’. Gene Set Enrichment Analysis (GSEA) of this core set of NAD-related pathways was then performed for initial differential outputs (NA week 1 vs. NA baseline, etc.) using the fgsea R package [51], with genes ranked by test statistics and significant enrichment defined at the FDR≤0.1 level. Key NAD genes were consequently defined as NA-specific genes (as above) that also manifested as leading-edge genes (i.e., genes that contribute most to enrichment signal) of NAD-related pathways displaying exclusive enrichment in the NA group (as per GSEA analysis).

### Statistical analysis

Data are expressed as mean ± standard error of the mean (SEM) unless otherwise stated. Normality was tested via the Kolmogorov-Smirnov test. Comparisons between NA and PLA group characteristics were analysed using unpaired t-tests. Characteristic comparisons (e.g., BMI) across time within treatment group were analysed using one-way ANOVA. Exercise capacity and protein turnover outcomes within NA or PLA groups during the supplementation period were analysed by repeated measures ANOVA/mixed effects model with a Bonferroni correction using Prism 8 (GraphPad Software, La Jolla, CA, USA), as done previously [52]. Cohens effect sizes (ES) were calculated and considered as follows; 0 to <0.20 considered ‘trivial’, 0.20 to <0.50 considered ‘small’, 0.50 to <0.80 considered ‘medium’ and ≥0.80 considered large [32]. Statistical significance was considered as p≤0.05.

For exploratory analyses of mitochondrial parameters, a Bayesian approach was used as recommended for small, exploratory clinical studies [53, 54]. A hierarchical mixed effects models with subject as a random intercept was constructed. Data were mean centred before modeling to ease the choice of mean values for priors. The model we estimated was:

$$y_{i}=\left(\beta_{0}+\tau_{i}\right)+\beta_{1}\times \text{tim}e_{i}+\beta_{2}\times \text{con}d_{i}+\beta_{3}\times \left(\text{tim}e_{i}\times \text{con}d_{i}\right)+\epsilon_{i}$$

where *y*_*i*_ is the mean centred value for each variable we assayed. The *τ*_*i*_ term is an offset from the intercept for each individual to accommodate the repeated measures nature of the data. The prior on *τ*_*i*_ was student *t* (3, 0, SD). The ϵ_i_ term represents the residuals with a prior set as student *t* (3, 0, SD). The dispersion parameter for these priors (SD) was set as the standard deviation of the data overall multiplied by 2. The prior on the overall intercept (*β*_0_) was set as Normal(0, SD) where SD was defined as the standard deviation of the data overall multiplied by 4. The slope parameters (*β*_1_, *β*_2_ & *β*_3_) were set to student t (3, 0, SD) where SD was defined as the standard deviation of the data for each variable (to accommodate variables on different scales) multiplied by 4. These priors were wide on the scale of the data and were chosen to provide more regularisation than the flat prior implied by the standard null hypothesis significance testing. Bayesian analysis gives rise to an entire posterior distribution rather than a maximum likelihood point estimate [55, 56]. As such there are several indices of “effect”. The probability of direction (pd) is defined as the proportion of the posterior distribution that is of the posterior median sign [57, 58] and is useful for assessing the direction of effect in exploratory analysis. Thus, we have chosen to present the pd as the effect index for our exploratory analysis of the effect of NA on mitochondrial parameters. All analyses were carried out using the Stan probabilistic programming language [59] via the brms package [60] in R (version 4.1.1) [61]. Further details, data and analysis scripts are available in Supplementary Analysis. Data and analysis scripts for the Bayesian analysis are available at https://zenodo.org/records/13255668 and (for the western blot data only) in the Supplementary Material.

## Results

### Volunteer characteristics and supplement tolerance

NA and PLA groups were well matched with no differences in baseline characteristics (Table 1). Since variations in diet and physical activity levels can have profound effects on muscle function and metabolism [23], both were monitored as essential control measures during the supplementation period. Habitual dietary and physical activity behaviours were similar between groups (all p>0.05, Table 2). Both NA and PLA supplementation had no effect on body mass index, heart rate or systolic or diastolic blood pressure (all p>0.05, Figure S2).

**Table 2:** Habitual dietary and physical activity behaviours during 2-week NA or PLA supplementation.

	NA	PLA	p-Value^a^
Daily step count	6,821 ± 1,246	8,005 ± 976	p=0.46
Energy, kcal d^−1^	1983 ± 135	2,130 ± 126	p=0.45
Carbohydrate, g d^−1^	231 ± 22	249 ± 18	p=0.54
protein, g d^−1^	80 ± 7	86 ± 6	p=0.52
Fat, g d^−1^	76 ± 4	83 ± 6	p=0.40

Data are mean ± S.E.M. ^a^comparisons between NA (n=8) and PLA (n=10) made by unpaired *t*-tests. NA, nicotinic acid; PLA, placebo.

Volunteer compliance to NA or PLA was 97 % (Table S1). Skin flushing presented in 3 of 8 NA volunteers (Table S1) but did not persist beyond the first day (i.e., beyond 3 NA doses) of the supplemental period. No side-effects were reported in PLA volunteers. NA was, therefore, well-tolerated and no volunteers dropped-out of the clinical trial due to NA side effects.

### NA effects on aerobic capacity and anaerobic sprint performance

To understand whether NA influences whole body substrate metabolism and aerobic muscle function, we performed gas exchange and ventilatory data analysis in response to moderate incremental and ramp incremental cycling. Indicative of an NA-related shift in substrate utilisation towards fat oxidation, the NA group displayed a main effect of time for respiratory exchange ratio (RER) at the beginning and end of moderate intensity exercise (p=0.005), where RER was unchanged after week 1 of supplementation but decreased by week 2 (baseline RER: p=0.045, ES=0.50; end exercise RER: p=0.006, ES=0.91) (Table 3). There was also a main effect of time for RER at baseline during ramp exercise in NA (p=0.057), where RER was again unchanged after week 1 of supplementation but decreased by week 2 (p=0.006, ES=1.56) (Table 3). There was no effect of NA on $\dot{\;V}$ O_2_, $\dot{\;V}$ CO_2_ or $\dot{\;V}$ _E_ at the beginning or end of exercise during moderate and ramp exercise, and no differences were observed in any aerobic performance measure in the PLA group (all p>0.05, Table 3). There was no change in SPPBT following NA or PLA intake (p>0.05, Figure S2).

**Table 3:** Ventilatory and gas exchange dynamics during moderate and ramp exercise at baseline, week 1 and week 2 post-NA or PLA supplementation.

	NA			PLA		
Moderate intensity exercise	Baseline (n=7)	Week 1 (n=7)	Week 2 (n=7)	Baseline (n=9)	Week 1 (n=7)	Week 2 (n=9)
Baseline V_O2_, l/min	0.68 ± 0.05	0.67 ± 0.04	0.68 ± 0.03	0.69 ± 0.03	0.66 ± 0.03	0.67 ± 0.03
End exercise V_O2_, l/min	0.94 ± 0.06	0.94 ± 0.06	0.96 ± 0.07	0.94 ± 0.04	0.96 ± 0.05	0.94 ± 0.05
Baseline V_CO2_, l/min	0.63 ± 0.04	0.62 ± 0.03	0.60 ± 0.03	0.62 ± 0.02	0.58 ± 0.02	0.61 ± 0.02
End exercise V_CO2_, l/min	0.91 ± 0.08	0.90 ± 0.07	0.88 ± 0.07	0.86 ± 0.04	0.88 ± 0.06	0.88 ± 0.05
Baseline RER	0.94 ± 0.02	0.93 ± 0.02	0.90 ± 0.02^a,#^	0.90 ± 0.02	0.88 ± 0.02	0.91 ± 0.03
End exercise RER	0.97 ± 0.03	0.96 ± 0.01	0.91 ± 0.03^a,#^	0.92 ± 0.02	0.92 ± 0.02	0.94 ± 0.02
Baseline V_E_, l/min	23 ± 2	22 ± 1	23 ± 1	23 ± 1	22 ± 1	22 ± 1
End exercise V_E_, l/min	31 ± 3	30 ± 2	31 ± 3	30 ± 1	31 ± 1	30 ± 1

Ramp exercise	Baseline (n=8)	Week 1 (n=8)	Week 2 (n=8)	Baseline (n=10)	Week 1 (n=9)	Week 2 (n=10)
Baseline V_O2_, l/min	0.65 ± 0.05	0.67 ± 0.03	0.69 ± 0.04	0.71 ± 0.03	0.69 ± 0.04	0.67 ± 0.02
Peak V_O2_, l/min	1.87 ± 0.14	1.90 ± 0.13	1.91 ± 0.13	1.92 ± 0.08	2.02 ± 0.13	1.99 ± 0.10
Baseline V_CO2_, l/min	0.59 ± 0.04	0.62 ± 0.03	0.59 ± 0.04	0.64 ± 0.03	0.60 ± 0.03	0.60 ± 0.02
Peak V_CO2_, l/min	2.38 ± 0.16	2.40 ± 0.15	2.33 ± 0.13	2.40 ± 0.10	2.51 ± 0.18	2.45 ± 0.13
Baseline RER	0.91 ± 0.02	0.92 ± 0.02	0.85 ± 0.01^a,#^	0.89 ± 0.02	0.88 ± 0.02	0.89 ± 0.02
Peak RER	1.29 ± 0.05	1.27 ± 0.03	1.25 ± 0.05	1.27 ± 0.04	1.26 ± 0.06	1.24 ± 0.03
Baseline V_E_, l/min	22 ± 1	23 ± 1	22 ± 1	23 ± 1	21 ± 1	21 ± 1
Peak V_E_, l/min	98 ± 7	98 ± 7	101 ± 10	95 ± 5	98 ± 7	97 ± 8

Data are mean ± S.E.M., within treatment comparisons made by repeated measures ANOVA (NA: n=7–8) or mixed-model analysis (PLA: n=7–10), with Bonferonni correction ^a^p<0.05 from baseline. ^#^p<0.05 from week 1. NA, nicotinic acid; PLA, placebo; RER, respiratory exchange ratio.

To assess the effects of NA intake on anaerobic muscle function, we examined maximal cycling sprint power producing capacity. Peak power, average power and fatigue index were unchanged during the 10 s Wingate test in both NA and PLA groups (all p>0.05, Table 4). A main effect of time was found for minimum power production in NA (p=0.033) and PLA (p=0.038) groups (Table 4), presenting after week 2 of supplementation, (NA: p=0.020, ES=0.75; PLA: p=0.007, ES=0.63), possibly reflecting a learning response to the Wingate protocol, despite previous familiarisation.

**Table 4:** Anaerobic performance at baseline, week 1 and week 2 post-NA or PLA supplementation.

	NA			PLA		
	Baseline	Week 1	Week 2	Baseline	Week 1	Week 2
Peak power, W/kg	6.48 ± 0.89	6.66 ± 0.78	6.99 ± 0.83	7.08 ± 0.43	7.25 ± 0.36	7.81 ± 0.38
Average power, W/kg	5.05 ± 0.73	4.98 ± 0.64	5.40 ± 0.68	5.49 ± 0.37	5.77 ± 0.34	6.12 ± 0.31
Minimum power, W/kg	1.81 ± 0.31	1.72 ± 0.56	2.67 ± 0.49^a,#^	1.62 ± 0.61	2.18 ± 0.71	2.86 ± 0.63^a^

Data are mean ± S.E.M., within treatment comparisons made by repeated measures ANOVA (NA: n=8; PLA: n=10), with Bonferonni correction. ^a^p<0.05 from baseline. ^#^p<0.05 from week 1. NA, nicotinic acid; PLA, placebo.

### NA effects on glucose tolerance

Fasting blood glucose concentrations did not differ from baseline to week 1 or week 2 in the NA or PLA groups (p>0.05, Figure S3), indicating NA had no impact on post-absorptive glucose levels. At all timepoints (i.e., baseline, week 1 and week 2), there was a significant effect of time (p<0.005), reflecting the increase in blood glucose concentrations in response to the oral glucose, in both NA and PLA groups (Figure S3A and C). No interaction effect was observed in either NA or PLA group (p>0.05), and no significant changes were observed in blood glucose AUC (p>0.05, Figure S3B and D), indicating that NA did not impact glucose handling.

### NA positively affected mitochondrial respiration

**Figure 3:**
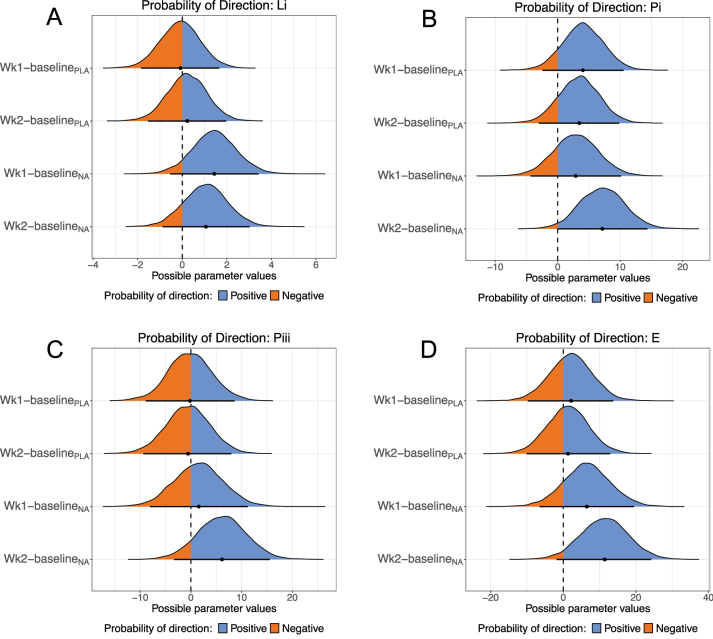
Probability of direction plots for mitochondrial respiratory states. For each respirometry variables assayed (A – Li, B – Pi, C – Pi+ii, D – E) the plots show the posterior distribution for the difference between time points in each treatment group. For each protein the differences are vertically week 1 – baseline and week 2 – baseline in the PLA group followed by the same differences in the NA group. Blue indicates positive values; orange indicates negative values.

To assess the mitochondrial effects of NA, we measured mitochondrial respiration in fresh muscle fibres. Baseline respiration slightly increased in the PLA group over the course of the study, while in the NA group baseline respiration initially rose at week 1 but then fell back to baseline at week 2 (Figure S4). In the PLA group, the probability of an increase in baseline respiration between baseline and week 1 was a modest 63.8 % but this increased between baseline and week 2–94 %. In the NA group, there was an 84 % probability of a positive change between baseline and week 1 and the probability of direction for the negative change between baseline and week 2 was 76 %. Leak (Li) direction of change remained flat over the study in the PLA group (Figure S4) with little probability for direction of change at either timepoint from baseline (week 1 pd=52.9 %; week 2 pd=60.3 %) (Figure 3A). In the NA group there was an increase in Li between baseline and week 1 with a pd of 92.5 % and a pd of 85.2 % for an increase from baseline to week 2 (Figures 3A and S4). Coupled respiration via complex I (Pi) increased from baseline to week 1 in both the PLA and NA groups (pd=89.3 % and pd=79.2 %, respectively), but whilst PLA remained stable at week 2 (pd=85.4 %) the NA group further increased Pi at week 2 (pd=97 %) (Figures 3B and S4). Maximal coupled respiration (Pi+ii) was mostly unchanged in the PLA group (Figure S4) with no indication of a change from baseline at week 1 (pd=51.3 %) or week 2 (pd=55.8 %) (Figure 3C). However, in the NA group, there was a modest increase in Pi+ii (Figure S4) at week 1 (pd=63.2 %), which was further strengthened at week 2 (pd=90.8 %) (Figure 3C). Maximal uncoupled respiration (E) was largely unaltered in the PLA group with modest pd of 64.9 % (baseline to week 1) and 58.7 % (baseline to week 2) (Figures 3D and S4). In contrast, E rose progressively from week 1 (pd=84.2 %) and week 2 (pd=95.9 %) (Figure 3D). Magnitudes and 95 % credible interval limits for these changes are shown in Table S2.

### NA positively affected mitochondrial content and ETC expression

**Figure 4:**
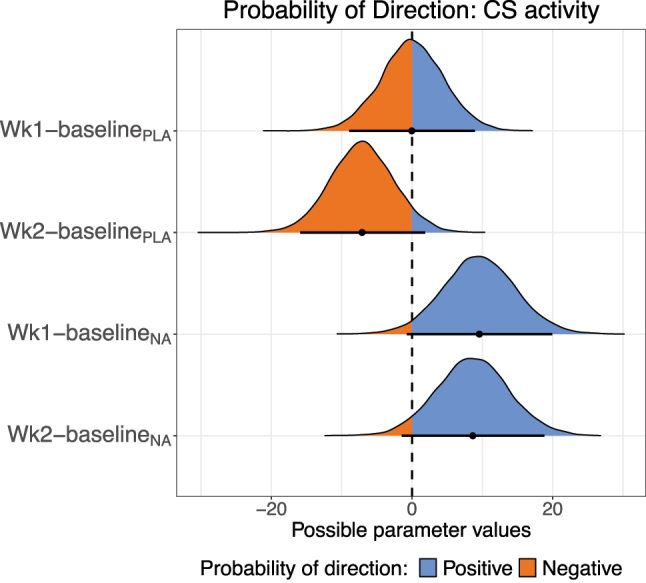
Probability of direction plot for citrate synthase (CS) activity. The plot shows the posterior distribution for the difference in CS activity between time points in each treatment group. The differences are vertically week 1 – baseline and week 2 – baseline in the PLA group followed by the same differences in the NA group. Blue indicates positive values; orange indicates negative values.

We examined CS activity as a marker of mitochondrial content in skeletal muscle (Figure S5). In the PLA group, CS activity was unchanged between baseline and week 1 (pd=50.4 %) (Figure 4) and CS activity was lower at week 2 than at baseline (Figure S5) with a robust probability for a negative change (pd=94.4 %) (Figure 4). In contrast, CS activity in the NA group displayed an increase between baseline and week 1 (pd=96.7 %) and then plateaued with a baseline to week 2 pd from baseline of 95.1 % (Figures 4 and S5). Magnitudes and 95 % credible interval limits for these changes are shown in Table S3.

**Figure 5:**
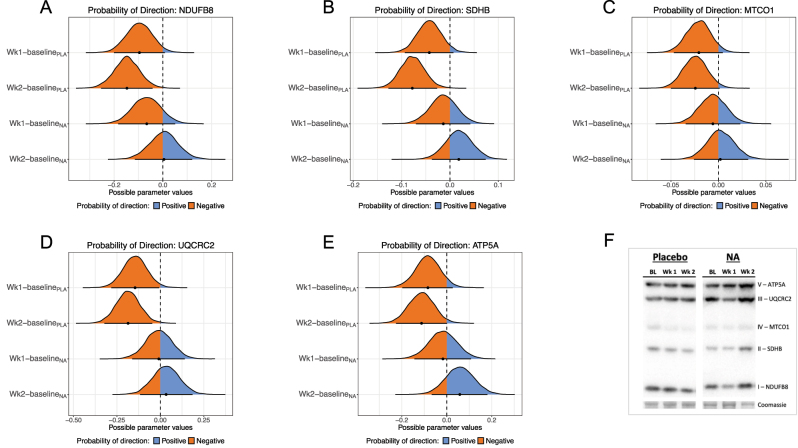
Probability of direction plots for ETC proteins. For each protein assayed (A – NDUFB8, B – SDHB, C – MTOC1, D – UQCRC2, E – ATP5A & F – representative western blots) the plots show the posterior distribution for the difference between time points in each treatment group. For each protein the differences are vertically week 1 – baseline and week 2 – baseline in the PLA group followed by the same differences in the NA group. Blue indicates positive values; orange indicates negative values.

We also examined the total expression of individual ETC complexes by Western blotting. In the PLA group, Bayesian analysis for each of the five ETC complexes revealed a pattern of strong reductions at both week 1 and week 2 of the study: NDUFB8 (pd=96.2 % week 1 and pd=99.7 % week 2), SDHB (pd=95.2 week 1 and pd=99.9 % week 2), MTCO1 (pd=94.9 % week1 and pd=96.7 % week 2), UQCRC2 (pd=98.2 % week 1 and pd=99.5 % week 2), ATP5A (pd=93 % week 1 and pd=97.6 % week 2) (Figures 5A–E and S6). Conversely, in the NA group, there was a pattern of marginally lower/unaltered probabilities of direction change from baseline at week 1 of supplementation, which generally remained unaltered from baseline or became a change by week 2: NDUFB (reduced pd=87.2 % week 1 and unaltered pd=54.3 % week 2), SDHB (reduced pd=68.2 % week 1 and increased pd=74.5 % week 2), MTCO1 (reduced pd=66 % week 1 and unaltered pd=56.2 % week 2), UQCRC2 (unchanged pd=54.5 % week 1 and pd=67.7 % week 2), ATP5A (unaltered pd=59.6 % week 1 and strongly increased pd=82.3 % week 2) (Figures 5A–E and S6). Magnitudes and 95 % credible interval limits for these changes are shown in Table S4.

### NA did not affect myofibrillar or mitochondrial muscle protein synthesis

**Figure 6:**
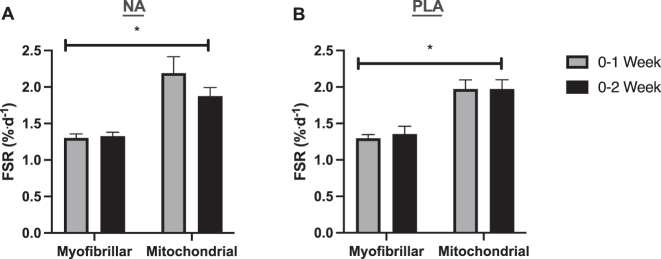
Skeletal muscle myofibrillar and mitochondrial fractional synthesis rates in response to NA (A) or PLA (B) supplementation. Data are expressed as mean ± SEM. Within treatment comparisons made by repeated measures ANOVA (NA: n=8) or mixed-model analysis (PLA: n=8–10). *p≤0.01 from myofibrillar. Grey bars = 0–1 week; black bars = 0–2 week. FSR, fractional synthesis rate; NA, nicotinic acid; PLA, placebo.

Within NA and PLA groups, there was a main effect of cumulative rates of myofibrillar and mitochondrial protein synthesis (NA: p=0.0007; PLA: p<0.0001) where mitochondrial FSR was consistently higher than myofibrillar FSR at weeks 1 and 2 of supplementation (Figure 6). However, NA had no effect on cumulative rates of myofibrillar (baseline to week 1 versus baseline to week 2, p>0.99, ES=0.14) or mitochondrial (baseline to week 1 versus baseline to week 2, p=0.13, ES=0.65) FSR (Figure 6A). Similarly, myofibrillar (baseline to week 1 versus baseline to week 2, p>0.99, ES=0.26) and mitochondrial (baseline to week 1 versus baseline to week 2, p>0.99, ES=0) FSR did not change in the PLA group (Figure 6B).

### Effects of NA on the expression of individual genes in older muscle

**Figure 7:**
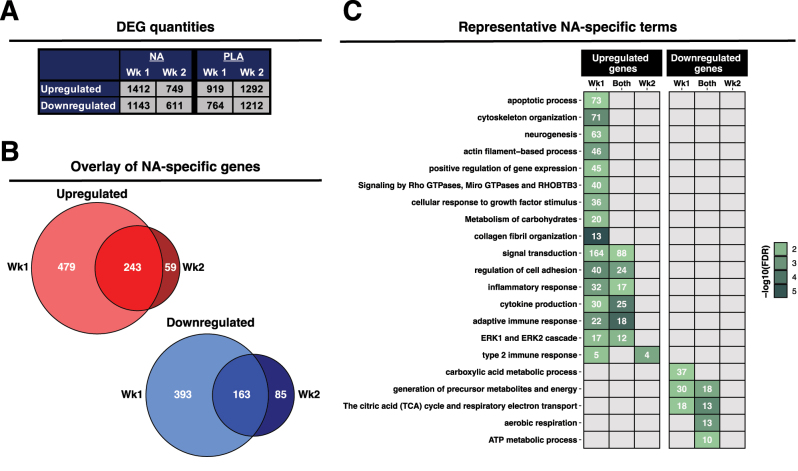
Untargeted analysis of the muscle transcriptome following NA supplementation. (A): Differential gene quantities at weeks 1 and 2 for the NA and PLA groups. (B): Venn diagrams illustrating the degree of overlap across time for genes specifically regulated by NA (i.e., genes differentially expressed at week 1 and/or week 2 in the NA group that are neither differentially expressed at week 1 nor week 2 in the PLA group). (C): Heatmap of representative enriched terms for NA-specific genes (i.e., representatives for NA-specific genes that are not also enriched for PLA-specific or generically-regulated genes). Enriched gene quantities are given within associated heatmap boxes, with colour shading strength denoting magnitude of enrichment significance (negative log10 of FDR). For all panels: NA, nicotinic acid; PLA, placebo; Wk, week; FDR, Benjamini-Hochberg false discovery rate; UR, upregulated; DR, downregulated.

We next investigated the impact of NA on muscle transcriptional responses. The total number of differentially expressed (DE) genes in the NA group was substantially greater than PLA at week one (NA: 2,555 vs. PLA: 1,683 DE genes), and this pattern was reversed at week two (NA: 1,360 vs. PLA: 2,504 DE genes) (Figure 7A, full lists of DE genes are provided in Table S5). NA uniquely altered the expression of 1,278 genes at week 1, which fell to 550 DE genes at week 2 (Figure 7B). Of these, 243 genes were commonly upregulated by NA at both week 1 and week 2 and were functionally related to cell adhesion regulation and inflammatory/immune processes (inflammatory response, cytokine production, adaptive immune response). 163 genes were commonly downregulated at both week 1 and 2 in the NA group, which were consistently related to aerobic metabolism (the citric acid (TCA) cycle and respiratory electron transport, aerobic respiration, ATP metabolic process). Genes uniquely upregulated at week 1 were associated with apoptotic process, cytoskeletal organisation and metabolism of carbohydrates, and genes uniquely downregulated were, again, associated with aerobic metabolism (carboxylic acid metabolic process, TCA cycle and respiratory electron transport) (Figure 7C). Of the far fewer genes uniquely upregulated at week 2, these were associated with the type 2 immune response, with no enriched terms for uniquely downregulated genes (Figure 7C).

### NA effects on NAD^+^ gene pathways

**Figure 8:**
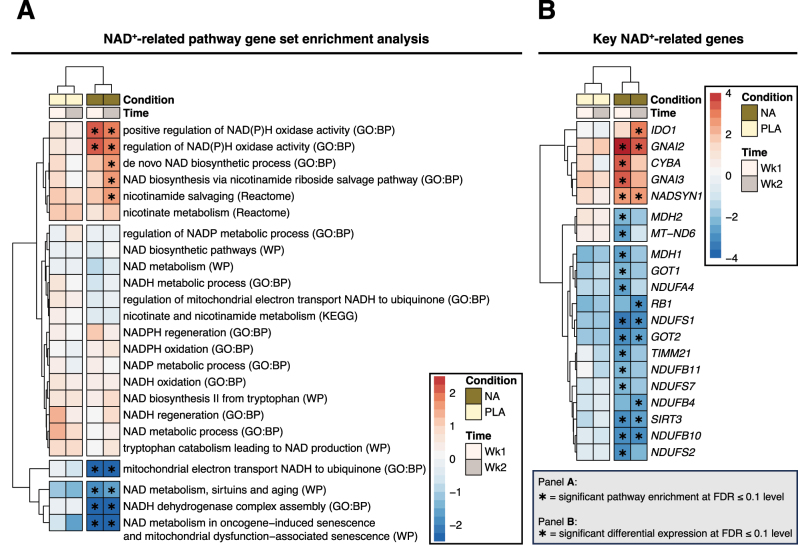
Muscle NAD-related gene signatures following NA supplementation. (A): Heatmap of results from Gene Set Enrichment Analysis (GSEA) of core NAD-related processes/pathways (24 in total). Dendrogram trees are defined based on clustering of the sign of each terms normalised enrichment score multiplied by the negative log10 of its enrichment p value, with this metric also used for colour shading within the heatmap to denote strength of enrichment significance. Asterisk denotes significant pathway enrichment (FDR≤0.1) in a given case. (B): Heatmap depicting key NAD genes (i.e., NA-specific genes that also manifest as leading-edge genes for NAD-related pathways with exclusive enrichment in the NA group, as per panel A). Dendrogram trees are defined based on clustering of depicted genes by their t-score, with this metric also used for heatmap colour shading. Asterisk denotes significant differential expression (FDR≤0.1) in a given case. For all panels: NA, nicotinic acid; PLA, placebo; Wk, week; FDR, Benjamini-Hochberg false discovery rate; NES, normalised enrichment score; DE, differential expression.

To probe whether NA impacted muscle gene networks specifically associated with NAD^+^ regulation and metabolism, we performed GSEA on a targeted set of NAD^+^-related pathways. In the NA group, gene pathways associated with NAD(P)H oxidase activity were significantly upregulated at both week 1 and 2, and pathways associated with NAD^+^ generation were significantly upregulated at week 2 (i.e., *de novo* NAD^+^ biosynthetic processes, NAD^+^ biosynthesis via nicotinamide riboside salvage pathway, nicotinamide salvaging) (Figure 8A). Supporting the NA-mediated upregulation of pathways related to NAD^+^ biosynthesis, specific genes facilitating the generation of NAD^+^ were significantly upregulated (e.g., NADSYN1) in the NA group at both timepoints (Figure 8B). NA supplementation was uniquely associated with the significant downregulation in gene pathways associated with NADH dehydrogenase complex assembly at both week 1 and 2, which was supported by the significant downregulation in multiple complex I-associated genes (i.e., *NDUFA4, NDUFB4, NDUFB10, NDUFB11, NDUFS1, NDUFS2, NDUFS7*) (Figure 8B).

## Discussion

Clinical evidence suggests the NAD^+^ precursor, NA, can ameliorate disease-associated mitochondrial dysfunction 33], [34], [35], [36. Since mitochondrial dysfunction also strongly associates with ageing muscle decline (e.g. [17]) we determined the efficacy of NA for improving muscle physical performance, metabolism and mitochondrial function, and associated molecular changes, in sedentary older people. We report that in inactive older males, two weeks NA supplementation significantly altered the NAD^+^-related transcriptome and there was a strong probability for increased mitochondrial function and content, which possibly manifested as increased fat utilisation at rest and during submaximal exercise. However, perhaps unsurprisingly given the brief 2 week supplement period, these molecular/metabolic effects failed to translate to muscle functional changes.

The observed strong probability of a positive NA-induced mitochondrial function effect, across all non-basal respiration states, presented after 1 week and tended to further increase (or was at least maintained) after 2 weeks NA intake. CS activity and expression of several ETC complexes also increased in parallel, signifying NA possibly improves mitochondrial health via both improved mitochondrial energetics and greater total mitochondrial volume. These mitochondrial improvements corresponded with an RER shift towards greater fat utilisation at rest and during submaximal exercise, as would be anticipated following enhanced mitochondrial capacity for lipid beta-oxidation [62], but only after 2 weeks of NA intake. Despite these metabolic effects, they did not translate into aerobic/anaerobic functional adaptation. Although mitochondrial volume density is closely associated with exercise capacity [63], increased mitochondrial volume density does not always directly translate into increased VO_2peak_ [64], but this does not necessarily mean that mitochondrial changes are not biologically/functionally important. It is plausible, therefore, that early NA-induced improvements in mitochondrial respiration accumulate with NA supplementation, ultimately manifesting as an improved capacity to oxidise fat – reflected in a lowered RER. Whether longer NA treatment adjunct to an exercise stimulus would translate to progressive improvements in mitochondrial function and fat metabolism, and overturn the lack of performance adaptation observed herein, should be explored.

Nonetheless, these results contradict reports employing NR in older people, where mitochondrial function, fuel oxidation or strength/endurance performance were unaltered, despite increased expression of NAD^+^ metabolome components [30]. Compared to NA, NR requires fewer enzymatic steps to produce NAD^+^ (two-steps via the salvage pathway) and skeletal muscle is enriched for these enzymes [65], hence the predominance of published studies utilising NR (e.g. [26, 29, 30, 32]). However, bioavailability of oral NR is purportedly poor [26, 30] and NR supplementation seems not to increase older muscle NR content [26, 30]. Muscle-enriched NAD^+^ salvage enzymes also decrease expression with ageing (e.g., NAMPT) [66, 67] which, combined, might account for the lack of NR efficacy in ageing muscle. NA alternatively generates NAD^+^ through the Preiss-Handler pathway, the enzymes of which (e.g., NaPRT) are expressed in human muscle [68] to efficiently synthesise NAD^+^ [65]. Additionally, gut epithelia express bilitranslocase, possessing specific affinity for NA to facilitate systemic NA bioavailability vs. other NAD^+^ precursors [69]. Our recruitment also focussed solely on sedentary volunteers, while others recruited recreationally active older people (e.g ([26]). Indeed, multiple tissues including muscle display age-correlated declines in NAD^+^ levels [17, 70], [71], [72. The combination of older age and sedentarism (vs. older age alone in previous NR studies) could possibly perturb muscle NAD^+^ homeostasis sufficiently to be responsive to NA. Whilst speculative, this would reflect a fundamental principle of the nutritional supplement field: that there is no scientific rationale for micronutrient intake to hold ergogenic or health-promoting qualities in healthy individuals, in the absence of preexisting micronutrient deficiency (e.g. [73]). Overall, our data suggest that employing NA, over NR, within specific populations (e.g. sedentary) tentatively promotes muscle health in older people.

One of the strongest NA-responsive transcriptomic signatures was increased expression of several cell-adhesion/muscle cell structure functional gene profiles. This finding echoes earlier studies of NAD^+^ precursor administration in pre-clinical models [74] and ageing human muscle [30], and infers a strong link between NAD^+^, mitochondrial health and gross muscle architectural integrity. Evidence suggests a bi-directional regulatory relationship between the mitochondria and cell adhesion/structure. In several animal models, adhesion complex proteins are required to maintain muscle structure and mitochondrial health [75, 76] and, in the opposite direction, mitochondrial function preserves muscle cell adhesion and, in-turn, muscle structure [75]. Congruent with NA effects on cell adhesion, NAD^+^ affects ADP-ribosylation of integrin receptors to augment cell adhesion processes (e.g., recruitment and binding of paxillin to laminin and integrin) [29], which could improve muscle architecture [67]. Thus, NA supplementation might facilitate cell adhesion both directly, and indirectly via improved mitochondrial function, to ultimately promote processes aimed at facilitating muscle integrity: a key long-term goal of ageing muscle therapies.

Specific analysis of NAD^+^ gene pathways identified NA-induced upregulation of several NAD^+^ biosynthetic gene clusters and NAD(P)H oxidase activity. Reinforcing the NA specificity of these muscle gene signatures, *NADSYN1* emerged as a top NA-responsive gene, which is involved in the conversion of NaAD to NAD^+^ in the Preiss-Handler pathway. The NA group also displayed downregulation of genes related to aerobic metabolism, the ETC, and NAD^+^ metabolism, concurring with previous human ageing NAD^+^ precursor interventions [30]. Interestingly, we observed consistent downregulation of several complex I subunits (*NDUFA4, NDUFB4, NDUFB10, NDUFB11, NDUFS1, NDUFS2, NDUFS7*), whose protein counterparts form the entry point of NAD^+^ into the ETC. While seemingly contradictory of improved mitochondrial content and function after NA intake, this might reflect enhanced mitochondrial efficiency and/or quality control (proteostasis, biogenesis, dynamic and mitophagy) to negate any functional requirement for transcriptional upregulation, as previously suggested [30].

### Limitations

A key limitation of this work is the relatively low volunteer numbers. Nonetheless, by implementing strict control measures (including the recruitment of inactive volunteers), employing a systems biological approach that encompasses multiple molecular/metabolic/functional endpoints, and applying statistical methodologies appropriate for the small, exploratory nature of this clinical study [53, 54], we have been able to detect mitochondrial-specific metabolic and molecular changes. Larger and longer clinical trials are warranted to validate the efficacy of NA for rejuvenating mitochondrial, and skeletal muscle health in older people. The standard clinical dose of NA used herein represents another study limitation, since this dose is not specific to ageing muscle but was chosen based on its efficacy for treating hyperlipidemia [41] and boosting mitochondrial function in type II diabetics [36]. As such, the optimal NA dosing regimen for augmenting muscle function in older adults may be refined.

The absence of non-esterified fatty acid (NEFA) analysis herein also precludes assessment of the ‘rebound’ effect, where acipimox treatment initiates an early (3 d) NEFA content decline, proceeded by a later (2–4 weeks) NEFA content increase [77]. However, since chronic NEFA elevation associates with impaired metabolism, including mitochondrial dysfunction 77], [78], [79], [80 and the rebound effect does not preclude acipimox-induced mitochondrial respiratory improvements in type-two diabetics [34], any rebound effect is unlikely to explain the present findings. Finally, whilst we did not observe increased mitochondrial synthesis rates with NA treatment, is likely that the modest mitochondrial volume changes reported fell below the limits of detection of our D_2_O stable isotope, mass spectrometry-based analytical method.

## Conclusions

We demonstrate that two weeks NA supplementation in heathy, sedentary, older males led to a strong probability of increasing mitochondrial function and content, underpinned by increased cell adhesion and decreased mitochondrial gene signatures, which potentially manifest as increased basal and submaximal fat substrate utilisation. These molecular and metabolic changes failed to translate to functional performance gains in this short, two-week intervention window. Thus, longer treatments in larger cohorts, and/or examination of potential synergistic effects of NA alongside aerobic exercise training are warranted.

## Supplementary Material

Supplementary Material

Supplementary Material

Supplementary Material

Supplementary Material

Supplementary Material

Supplementary Material

Supplementary Material

Supplementary Material

Supplementary Material

Supplementary Material

Supplementary Material

Supplementary Material

Supplementary Material
