# Supplementary figures and images for: Nicotinic acid improves mitochondrial function and associated transcriptional pathways in older inactive males
Source: Transl Exerc Biomed. 2024 Nov 25;1(3-4):277–94. doi: 10.1515/teb-2024-0030 (PMC11653476; doi:10.1515/teb-2024-0030)

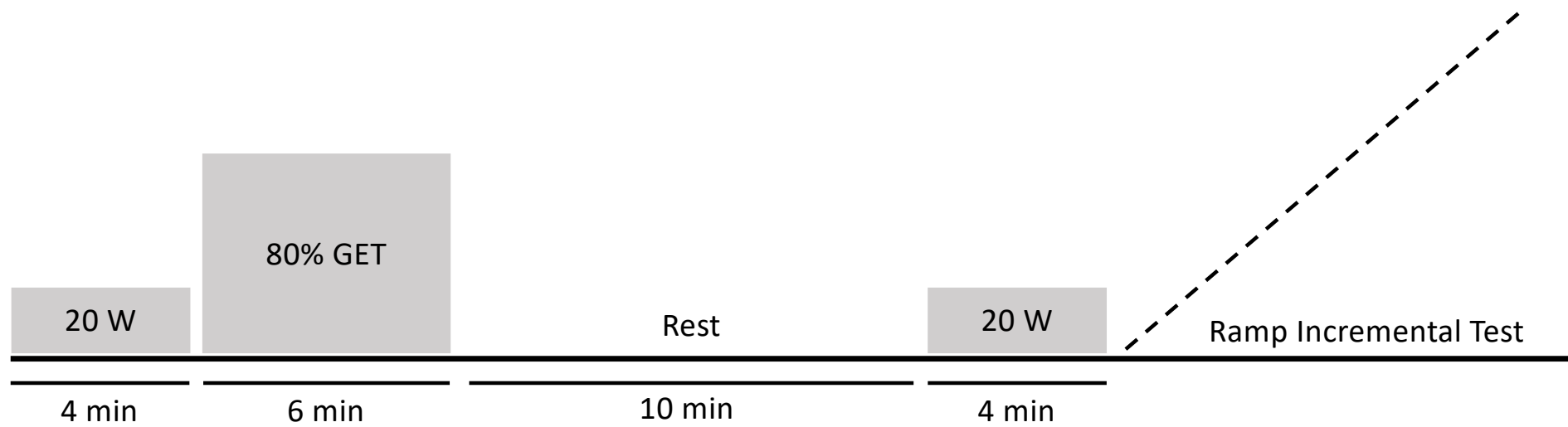

Supplement: Supplementary file 1 — Supplementary Material [file j_teb-2024-0030_suppl_001.pdf]

NA

A

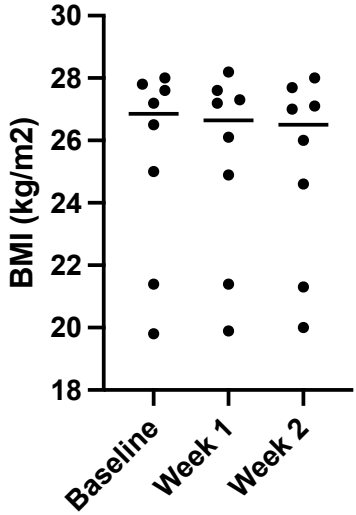

B

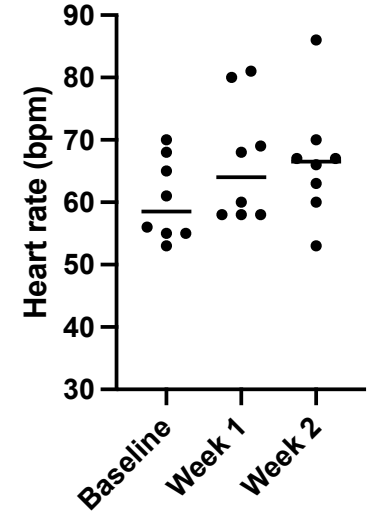

C

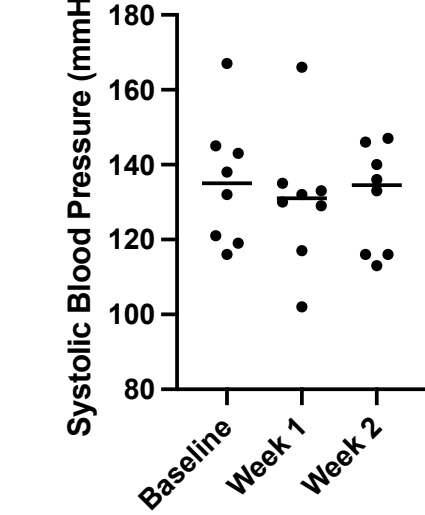

D

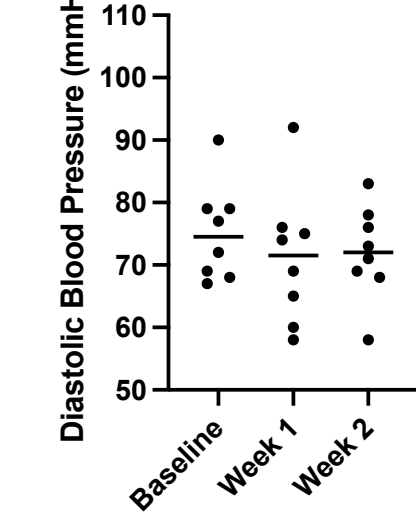

E

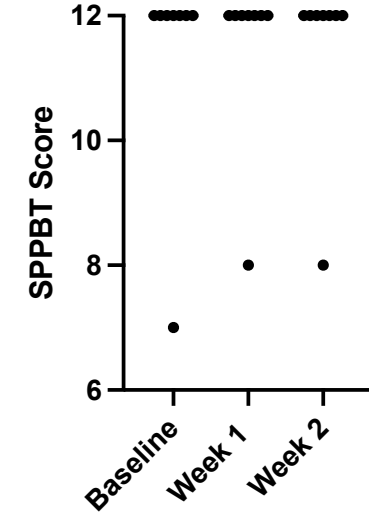

PLA

F

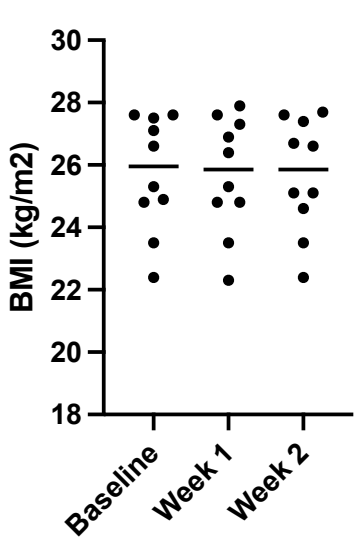

G

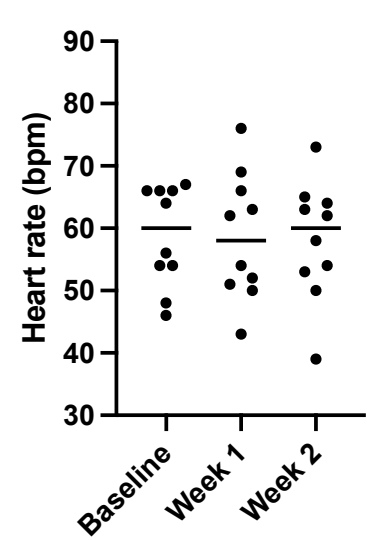

H

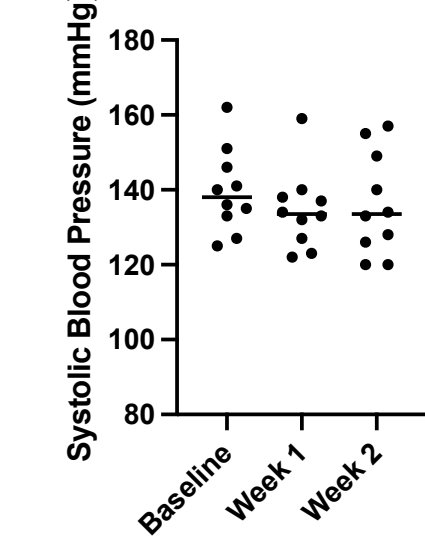

I

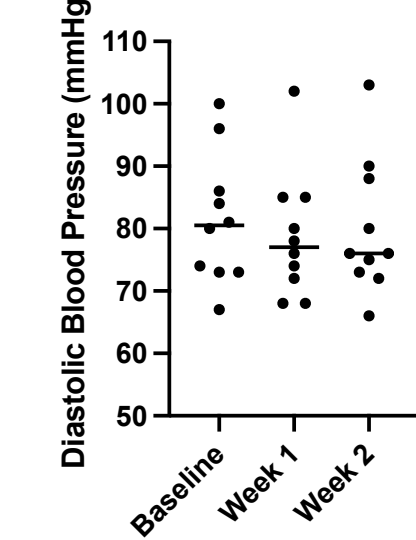

J

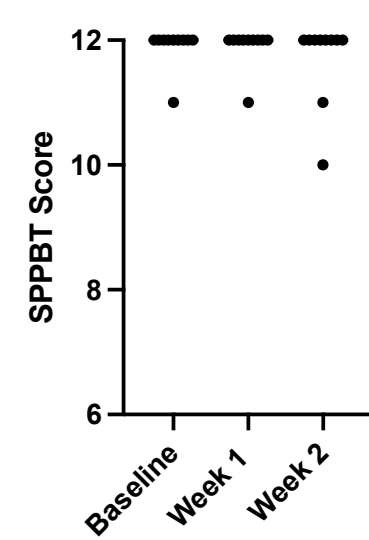

Supplement: Supplementary file 2 — Supplementary Material [file j_teb-2024-0030_suppl_002.pdf]

NA

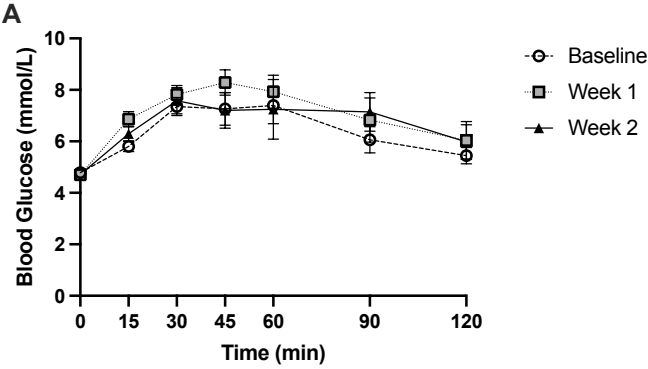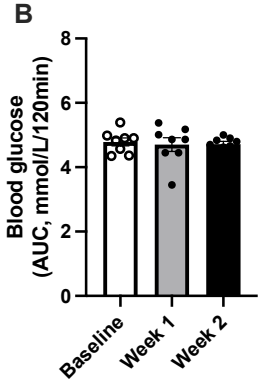

PLA

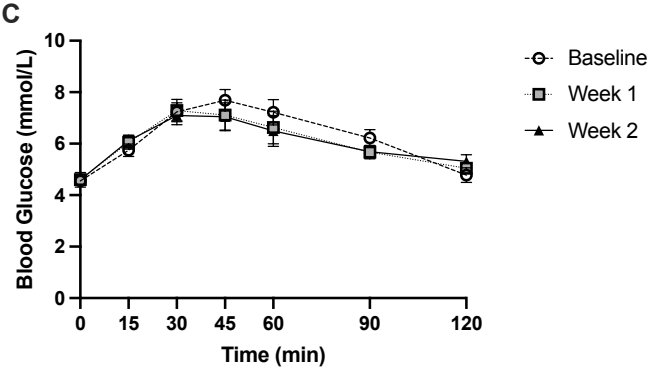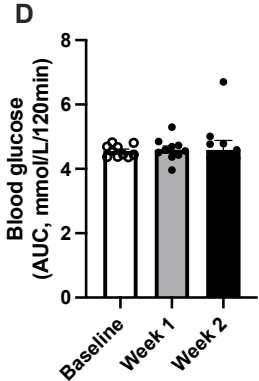

Supplement: Supplementary file 3 — Supplementary Material [file j_teb-2024-0030_suppl_003.pdf]

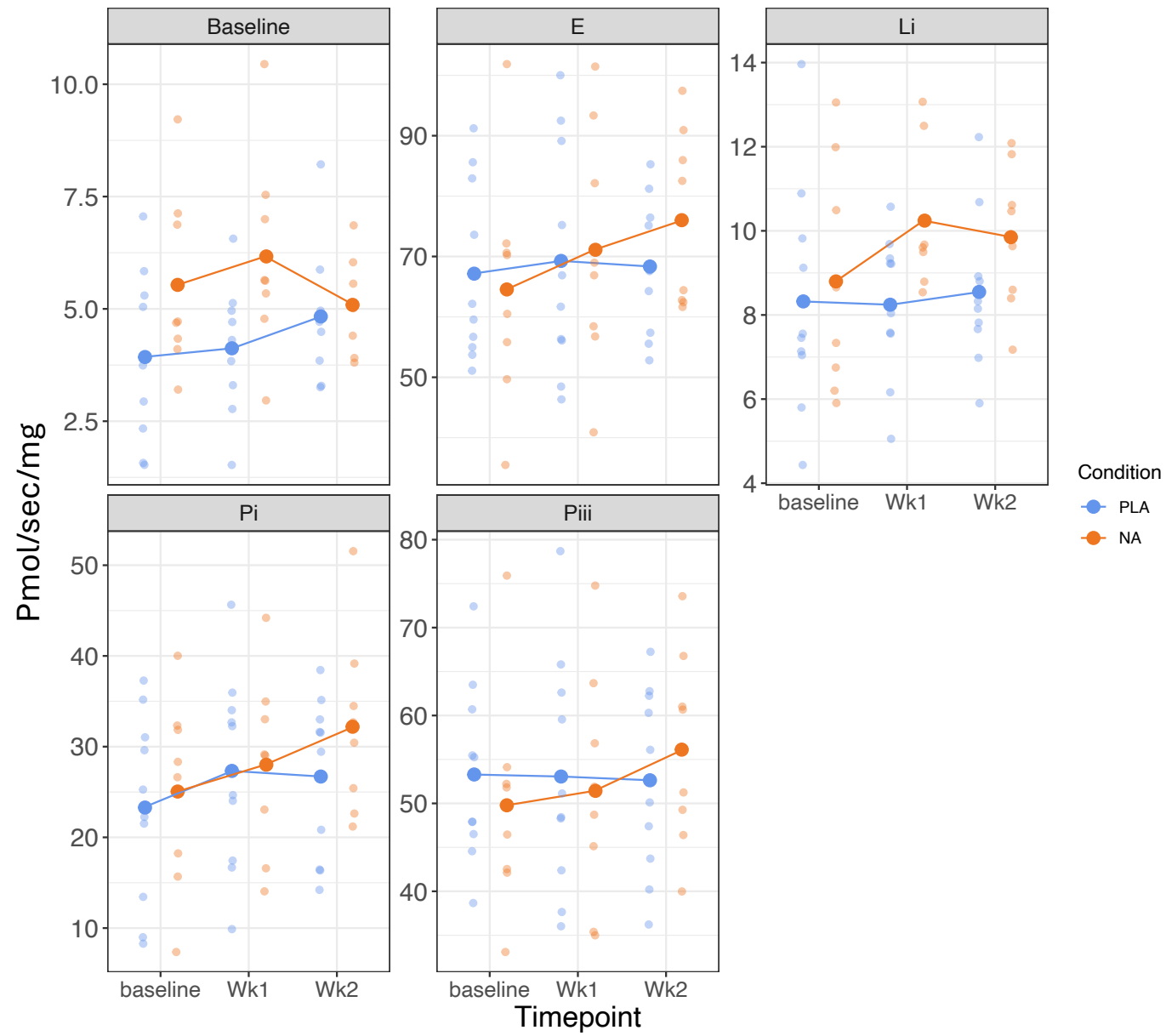

Supplement: Supplementary file 4 — Supplementary Material [file j_teb-2024-0030_suppl_004.pdf]

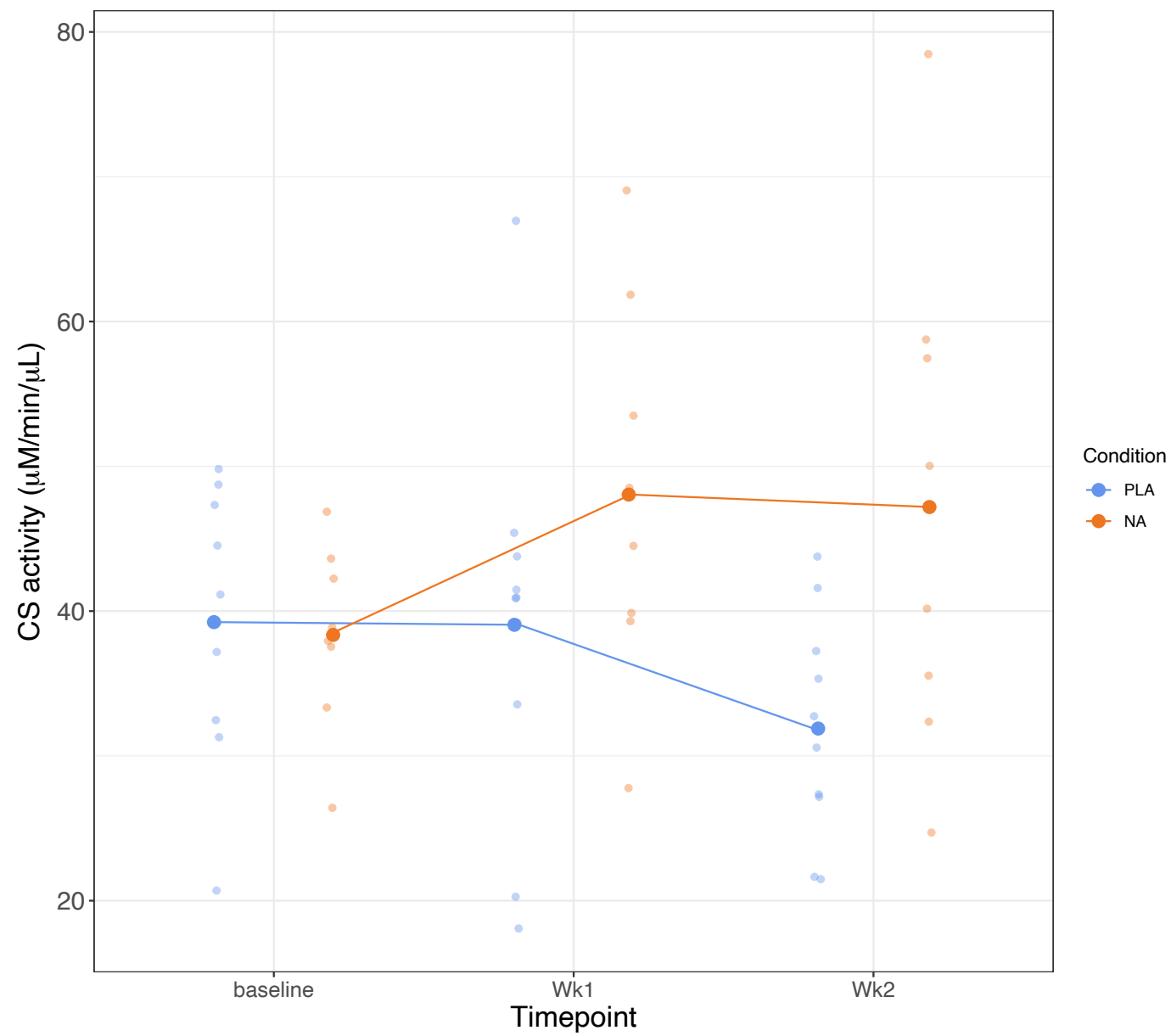

Supplement: Supplementary file 5 — Supplementary Material [file j_teb-2024-0030_suppl_005.pdf]

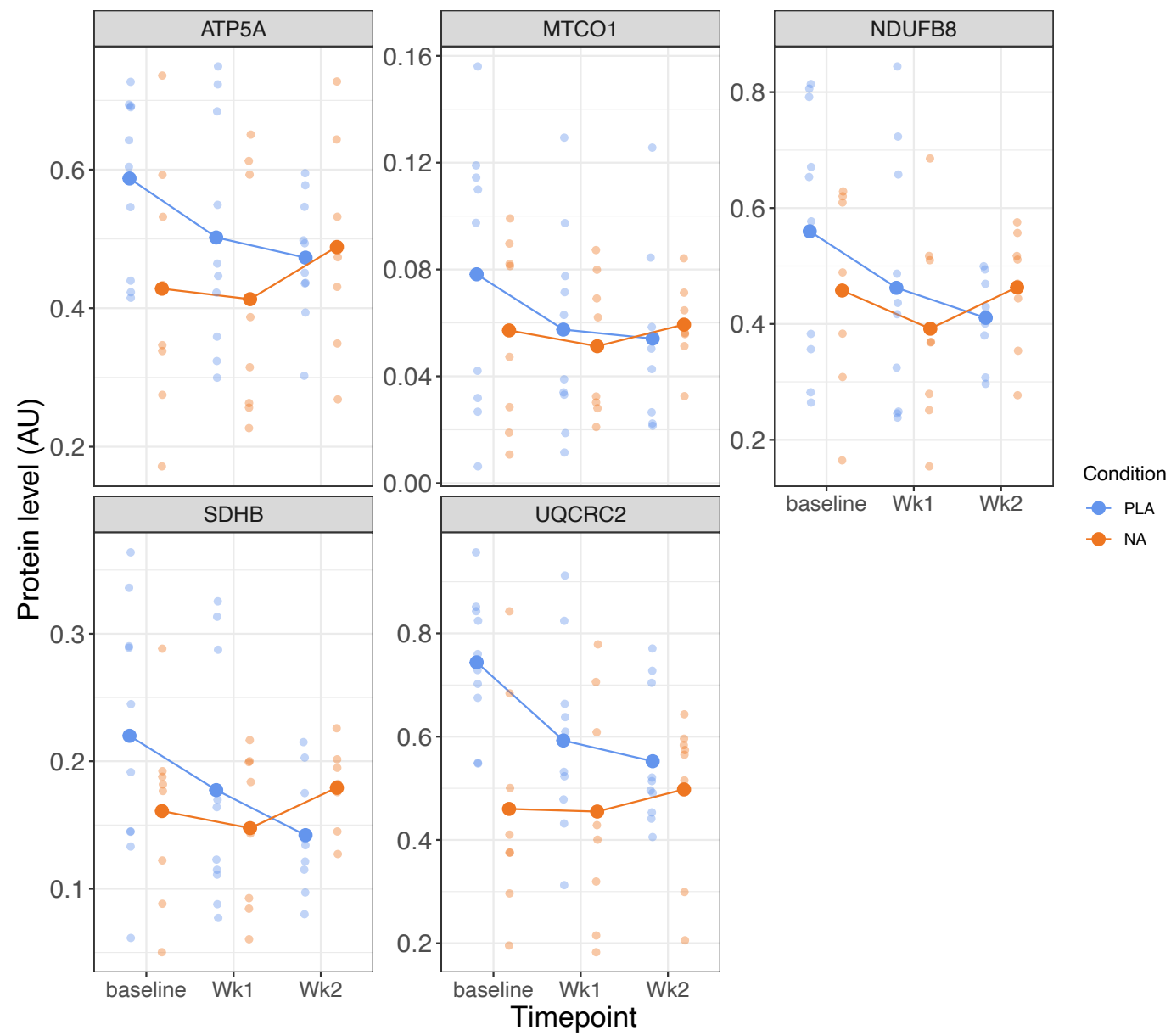

Supplement: Supplementary file 6 — Supplementary Material [file j_teb-2024-0030_suppl_006.pdf]
