# Supplementary material for: Nicotinic acid improves mitochondrial function and associated transcriptional pathways in older inactive males
Source: Transl Exerc Biomed. 2024 Nov 25;1(3-4):277–94. doi: 10.1515/teb-2024-0030 (PMC11653476; doi:10.1515/teb-2024-0030)
Supplement: Supplementary file 7 — Supplementary Material [file j_teb-2024-0030_suppl_007.docx]

**Table S1.** Volunteer compliance with nicotinic acid (NA; *n*=8) or placebo (PLA; *n*=10)

| **Volunteer** | **NA** | | | **PLA** | | |
| --- | --- | --- | --- | --- | --- | --- |
|  | Compliance (%) | Supplements missed  (out of 42) | Reported side effects | Compliance (%) | Supplements missed  (out of 42) | Reported side effects |
| 1 | 100 | 0 | None | 92.9 | 3 | None |
| 2 | 97.6 | 1 | Flushing | 100 | 0 | None |
| 3 | 100 | 0 | Flushing | 97.6 | 1 | None |
| 4 | 100 | 0 | Flushing | 97.6 | 1 | None |
| 5 | 97.6 | 1 | None | 100 | 0 | None |
| 6 | 100 | 0 | None | 100 | 0 | None |
| 7 | 78.6 | 9 | None | 90.5 | 4 | None |
| 8 | 100 | 0 | None | 100 | 0 | None |
| 9 | - | - | - | 92.9 | 3 | None |
| 10 | - | - | - | 97.6 | 1 | None |
| **Average Compliance (%)** | **97** | | | **97** | | |
