# Supplementary material for: Nicotinic acid improves mitochondrial function and associated transcriptional pathways in older inactive males
Source: Transl Exerc Biomed. 2024 Nov 25;1(3-4):277–94. doi: 10.1515/teb-2024-0030 (PMC11653476; doi:10.1515/teb-2024-0030)
Supplement: Supplementary file 8 — Supplementary Material [file j_teb-2024-0030_suppl_008.docx]

**Table S2.** Posterior medians, 95% credible interval limits and probability of direction of defined contrasts for respirometry variables in pmol/sec/mg.

| Contrast | Median | CI low | CI high | pd |
| --- | --- | --- | --- | --- |
| Baseline |  |  |  |  |
| PLA: Wk1-baseline | 0.20 | -0.95 | 1.30 | 0.64 |
| PLA: Wk2-baseline | 0.89 | -0.21 | 2.02 | 0.94 |
| NA: Wk1-baseline | 0.64 | -0.57 | 1.84 | 0.85 |
| NA: Wk2-baseline | -0.43 | -1.64 | 0.79 | 0.76 |
| Li |  |  |  |  |
| PLA: Wk1-baseline | -0.06 | -1.80 | 1.64 | 0.53 |
| PLA: Wk2-baseline | 0.22 | -1.51 | 1.94 | 0.60 |
| NA: Wk1-baseline | 1.41 | -0.54 | 3.35 | 0.93 |
| NA: Wk2-baseline | 1.02 | -0.90 | 2.97 | 0.85 |
| Pi |  |  |  |  |
| PLA: Wk1-baseline | 3.94 | -2.61 | 10.50 | 0.89 |
| PLA: Wk2-baseline | 3.34 | -3.11 | 9.95 | 0.85 |
| NA: Wk1-baseline | 2.95 | -4.33 | 10.10 | 0.79 |
| NA: Wk2-baseline | 7.12 | -0.30 | 14.29 | 0.97 |
| Pi+ii |  |  |  |  |
| PLA: Wk1-baseline | -0.16 | -8.49 | 8.11 | 0.51 |
| PLA: Wk2-baseline | -0.60 | -8.86 | 7.77 | 0.56 |
| NA: Wk1-baseline | 1.57 | -7.71 | 11.18 | 0.63 |
| NA: Wk2-baseline | 6.24 | -3.31 | 15.73 | 0.91 |
| E |  |  |  |  |
| PLA: Wk1-baseline | 2.23 | -9.46 | 13.68 | 0.65 |
| PLA: Wk2-baseline | 1.28 | -10.34 | 12.98 | 0.59 |
| NA: Wk1-baseline | 6.58 | -6.59 | 19.51 | 0.84 |
| NA: Wk2-baseline | 11.41 | -1.60 | 24.36 | 0.96 |
