# Supplementary material for: Nicotinic acid improves mitochondrial function and associated transcriptional pathways in older inactive males
Source: Transl Exerc Biomed. 2024 Nov 25;1(3-4):277–94. doi: 10.1515/teb-2024-0030 (PMC11653476; doi:10.1515/teb-2024-0030)
Supplement: Supplementary file 9 — Supplementary Material [file j_teb-2024-0030_suppl_009.docx]

**Table S3.** Posterior medians, 95% credible interval limits and probability of direction of defined contrasts for citrate synthase activity in uM/min/uL.

| Contrast | Median | CI low | CI high | pd |
| --- | --- | --- | --- | --- |
| CS activity |  |  |  |  |
| PLA: Wk1-baseline | 0.04 | -8.94 | 9.02 | 0.50 |
| PLA: Wk2-baseline | -7.18 | -16.15 | 1.86 | 0.94 |
| NA: Wk1-baseline | 9.51 | -0.65 | 19.73 | 0.97 |
| NA: Wk2-baseline | 8.57 | -1.76 | 18.57 | 0.95 |
