# Supplementary material for: Nicotinic acid improves mitochondrial function and associated transcriptional pathways in older inactive males
Source: Transl Exerc Biomed. 2024 Nov 25;1(3-4):277–94. doi: 10.1515/teb-2024-0030 (PMC11653476; doi:10.1515/teb-2024-0030)
Supplement: Supplementary file 10 — Supplementary Material [file j_teb-2024-0030_suppl_010.docx]

**Table S4.** Posterior medians, 95% credible interval limits and probability of direction of defined contrasts for each protein assayed by western blot.

| Contrast | Median | CI low | CI high | pd |
| --- | --- | --- | --- | --- |
| NDUFB8 |  |  |  |  |
| PLA: Wk1-baseline | -0.10 | -0.20 | 0.01 | 0.96 |
| PLA: Wk2-baseline | -0.15 | -0.25 | -0.04 | 1.00 |
| NA: Wk1-baseline | -0.07 | -0.18 | 0.06 | 0.87 |
| NA: Wk2-baseline | 0.01 | -0.11 | 0.12 | 0.54 |
| SDHB |  |  |  |  |
| PLA: Wk1-baseline | -0.04 | -0.09 | 0.01 | 0.95 |
| PLA: Wk2-baseline | -0.08 | -0.13 | -0.03 | 1.00 |
| NA: Wk1-baseline | -0.01 | -0.07 | 0.04 | 0.68 |
| NA: Wk2-baseline | 0.02 | -0.04 | 0.07 | 0.75 |
| MTCO1 |  |  |  |  |
| PLA: Wk1-baseline | -0.02 | -0.05 | 0.00 | 0.95 |
| PLA: Wk2-baseline | -0.02 | -0.05 | 0.00 | 0.97 |
| NA: Wk1-baseline | -0.01 | -0.03 | 0.02 | 0.66 |
| NA: Wk2-baseline | 0.00 | -0.03 | 0.03 | 0.56 |
| UQCRC2 |  |  |  |  |
| PLA: Wk1-baseline | -0.15 | -0.28 | -0.01 | 0.98 |
| PLA: Wk2-baseline | -0.19 | -0.32 | -0.05 | 1.00 |
| NA: Wk1-baseline | -0.01 | -0.16 | 0.14 | 0.55 |
| NA: Wk2-baseline | 0.03 | -0.12 | 0.19 | 0.68 |
| ATP5A |  |  |  |  |
| PLA: Wk1-baseline | -0.08 | -0.20 | 0.03 | 0.93 |
| PLA: Wk2-baseline | -0.11 | -0.22 | 0.00 | 0.98 |
| NA: Wk1-baseline | -0.02 | -0.14 | 0.11 | 0.60 |
| NA: Wk2-baseline | 0.06 | -0.07 | 0.18 | 0.82 |
