# Supplementary material for: Nicotinic acid improves mitochondrial function and associated transcriptional pathways in older inactive males
Source: Transl Exerc Biomed. 2024 Nov 25;1(3-4):277–94. doi: 10.1515/teb-2024-0030 (PMC11653476; doi:10.1515/teb-2024-0030)
Supplement: Supplementary file 12 — Supplementary Material [file j_teb-2024-0030_suppl_014.docx]

**SUPPLEMENTAL ANALYSIS**

**Western blot Bayesian analysis**

**Introduction**

This study examined the effects of acipimox, an NAD+ precursor on mitochondrial function. There were two treatment groups: placebo (n = 10) and acipimox (n = 8). Participants had muscle biopsies at baseline and at 1- & 2-weeks following placebo/acipimox treatment. Western blotting was used to gather data on the relative level of several mitochondrial proteins in skeletal muscle. These were:

- ATP5A
- MTCO1
- NDUFB8
- SDHB
- UQCRC2

In this analysis we will use a Bayesian hierarchical approach to model the average change in each mitochondrial component over time in the placebo and acipimox groups. Only results for ATP5A are shown. A different protein can be selected in the *Statistical modeling* section below.

**Packages & environement**

First, we load the packages we will need and make sure we start with clean environment.

library(brms) # bayesian modeling
library(bayesplot) # diagnostic plots etc
library(tidyverse) # data wrangling
library(gridExtra) # multiplot figures
library(tidybayes) # plots; distributions
library(easystats) # model summaries
library(priorsense) # for prior robustness checking; remotes::install_github("n-kall/priorsense")
library(gt) # tables
library(lme4) # frequentist analysis
theme_set(theme_bw())

# start clean
rm(list = ls())

Next, we load the data. The data is in long format (each row represents one measure) & contains the following variables:

- subject - subject ID
- protein - mitochondrial component
- timepoint - the timepoint (baseline, w1 & w2)
- condition - the condition (placebo, acipimox)
- value - the value of the protein from western blot in arbitrary units (AU)

We first convert the subject, protein, timepoint and condition variables to factors & reverse the order of the condition variable so placebo is the reference category for plotting and modeling.

wb_data <- read_csv("data/WB_long_format.csv")
# factors from subj, protein, timepoint & condition
wb_data <- wb_data |>
 mutate(across(where(is.character), as.factor))
# reverse order of placebo and acipimox for plots & model etc
wb_data <- wb_data |>
 mutate(condition = fct_rev(condition))

**Exploratory plots**

Next, we create an exploratory plot.

# set plot colours
clrs <- c("cornflowerblue", "chocolate2")

# all proteins over intervention b -> w1 -> w2
wb_data |>
 summarise(mn = mean(value), .by = protein:condition) |>
 ggplot(aes(timepoint, mn, colour = condition)) +
 geom_point(position = position_jitterdodge(jitter.width = 0.05), size = 3) + # point at mean
 geom_line(aes(group = condition), position = position_jitterdodge(jitter.width = 0.05)) + # lines
 geom_point(data = wb_data, aes(timepoint, value, fill = condition),
 position = position_jitterdodge(jitter.width = 0.05),
 alpha = 0.4) + # indiv points
 # x labels
 scale_x_discrete(labels=c("baseline" = "B",
 "w1" = "W1",
 "w2" = "W2")) +
 # legend labels & plot colours
 scale_colour_manual(values = clrs,
 name = "Condition", labels = c("Placebo", "Acipimox")) +
 labs(x = "Timepoint", y = "Protein level (AU)") +
 facet_wrap(~protein, scales = "free_y")

| 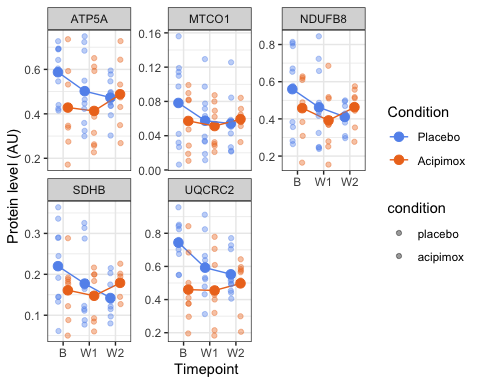  Figure 1: Exploratory figure |
| --- |

**Statistical modeling**

We will build Bayesian hierarchical (mixed) models for each protein; this document shows results for ATP5A only. The “random” term in these models will be the subject variable to account for repeated observations from each participant across the study timeline.

First, we select a protein to model and create some summary statistics. We also mean center the data as this makes the later specification of priors easier.

# select protein here
# ATP5A, MTCO1, NDUFB8, SDHB, UQCRC2
poi <- "ATP5A"
# filter to one protein
model_data <- wb_data |>
 filter(protein == poi)

# summary stats
# fname <- paste("tables/", poi, "_summary.docx", sep = "")
model_data |>
 summarise(Mean = mean(value), Std = sd(value), .by =c(timepoint, condition)) |>
 gt() |>
 tab_header(paste("Summary data for ", poi, sep = "")) |>
 fmt_number(decimals = 2) |>
 cols_align(columns = "timepoint", align = c("left"))

Table 1: Summary data for ATP5A

| timepoint | condition | Mean | Std |
| --- | --- | --- | --- |
| baseline | placebo | 0.59 | 0.12 |
| w1 | placebo | 0.50 | 0.17 |
| w2 | placebo | 0.47 | 0.09 |
| baseline | acipimox | 0.43 | 0.18 |
| w1 | acipimox | 0.41 | 0.18 |
| w2 | acipimox | 0.49 | 0.15 |

# center data
model_data <- model_data |>
 mutate(c_value = value - mean(value))

###

**Setting priors**

The model we will estimate is:

$$y_{i}=\left( \beta_{0}+\sigma_{i} \right)+\beta_{1}\times time_{i}+\beta_{2}\times cond_{i}+\beta_{3}\times\left( time_{i}\times cond_{i} \right)+\epsilon_{i}$$

where $\sigma$ is the random intercept variance and $\epsilon$ is the residual variance.

We need to set priors on each of the model parameters we are estimating. Here we estimate an overall intercept ($\beta_{0}$), a main effect of treatment ($\beta_{1}$), a main effect of time ($\beta_{2}$), the interaction between timepoint & condition ($\beta_{3}$), the variation in subject specific intercepts ($\sigma$) and the residual variation ($\epsilon$).

**Intercept prior**

The overall intercept is expected value when all other coefficients in a linear model are zero. In our context this is the mean of the placebo treatment at baseline. The data have been mean centered so we will set the prior mean at zero. With a lack of information on the centered values we would expect from western blot data a sensible prior for the overall intercept would be $\sim N\left( 0, 4\times sd of the data \right)$. The mean is our most plausible value for the overall intercept but it is plausible the overall intercept could vary by the four times amount of variability we see in the data overall. Whilst this is wide from a biological perspective it provides much more regularisation than the flat prior implied by the usual frequentist tests.

# intercept priors
int_prior_sd <- wb_data |>
 summarise(sds = ceiling(sd(value)*4)) |>
 pull()

####

**Slope and variance priors**

Next, we have to set priors for the $\beta$ (slope) coefficients in our model, the variance in the intercepts ($\sigma$) and the residual variance ($\epsilon$). For the slope coefficients we will use a student t prior with 3 degrees of freedom & a mean of zero (slopes could be positive or negative). The values for each protein are all approximately on the same scale so we will use the same dispersion value for the prior as we used for the model intercept.

For the intercept variation and residual variation, we use a $t$ distribution with a dispersion set to twice that of the data we will model but we limit the plausible values to only positive values (lb = 0; lower bound = 0) because variances cannot be negative. These variance priors suggest that the low variances are most likely but allow room for larger variance if the data suggests that. We use the stanvar() function to make the user derived values available to the the Stan language which will do the MCMC sampling for us via the brms package.

#! label: sd priors
# sd prior
sd_prior <- model_data |>
 summarise(sds = ceiling(sd(c_value))*2) |>
 pull()

# set prior; use stanvars
stanvars <- stanvar(int_prior_sd, name="int_prior_sd") +
 stanvar(sd_prior, name = "sd_prior")

rnd_int_priors <- c(prior(normal(0, int_prior_sd), class = Intercept), # intercept on mean of ALL data
 prior(student_t(3, 0, int_prior_sd), class = b), # beta coef prior
 prior(student_t(3, 0, sd_prior), class = sd, lb = 0), # random intercept sd (sigma)
 prior(student_t(3, 0, sd_prior), class = sigma, lb = 0)) # residual variance (epsilon)

We can plot these priors to examine plausible values for each of the parameters we are trying to estimate.

# plot priors
rnd_int_priors |>
 parse_dist() |>
 ggplot(aes(y = paste(class, "~", format(.dist_obj)), xdist = .dist_obj)) +
 stat_halfeye(.width = 0.95,
 slab_linewidth = 0.5,
 slab_color = "black",
 slab_fill = "cadetblue") +
 labs(title = paste(poi, "Priors", sep = " "),
 x = "AU",
 y = NULL)

| 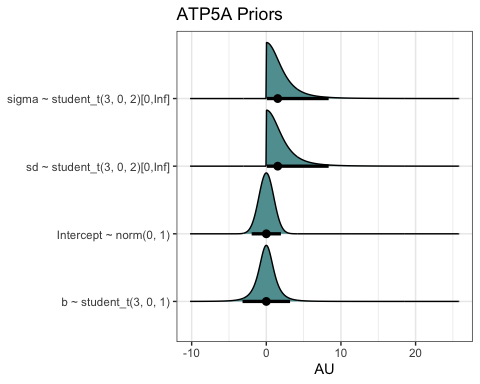  Figure 2: Priors used in the analysis |
| --- |

Next, we carry out prior predictive checks. Prior predictive checks involve simulating data from the model as defined by the priors *in the absence of data* and examining how compatible those simulations are with values we might expect. Here the priors have been chosen to be wide on the scale of the data and so our simulated data should also be more variable (wider) than the data we have but not wholly unreasonable.

# prior predictive check
prior_pred <- model_data |>
 brm(formula = c_value ~ timepoint * condition + (1|subject),
 prior = rnd_int_priors,
 family = gaussian(),
 cores = 4,
 iter = 2000,
 sample_prior = "only",
 stanvars=stanvars)

We examine the coefficients from the prior predictive model.

# check
parameters(prior_pred, effects = "fixed") |>
 gt() |>
 tab_header(title = paste(poi, " Prior predicitve check", sep = "")) |>
 fmt_number(decimal = 3) |>
 cols_hide(c("Component", "CI")) |>
 tab_header("Prior predicitve model coefficients")

Table 1: Prior predicitve model coefficients

| Parameter | Median | CI_low | CI_high | pd | Rhat | ESS |
| --- | --- | --- | --- | --- | --- | --- |
| b_Intercept | 0.009 | -2.874 | 2.916 | 0.502 | 0.999 | 3,096.874 |
| b_timepointw1 | 0.004 | -3.067 | 3.317 | 0.501 | 1.003 | 2,238.356 |
| b_timepointw2 | -0.012 | -3.204 | 3.345 | 0.507 | 1.000 | 3,149.492 |
| b_conditionacipimox | -0.016 | -3.095 | 3.490 | 0.505 | 1.001 | 1,958.278 |
| b_timepointw1:conditionacipimox | 0.010 | -3.249 | 2.855 | 0.504 | 1.000 | 1,944.702 |
| b_timepointw2:conditionacipimox | -0.019 | -3.240 | 3.131 | 0.508 | 1.000 | 2,266.881 |
| sigma | 1.512 | 0.060 | 8.367 | 1.000 | 1.000 | 4,007.852 |

Next, we generate plots to examine how the MCMC sampling process for the model has converged and the distribution of the model predictions without any data input. First we examine a histogram of the prior model predictions and MCMC traceplots.

# model sampling check
mcmc_hist(prior_pred, regex_pars = "^b_", pars = "sigma")

`stat_bin()` using `bins = 30`. Pick better value with `binwidth`.

mcmc_trace(prior_pred, regex_pars = "^b_", pars = "sigma")

| \| 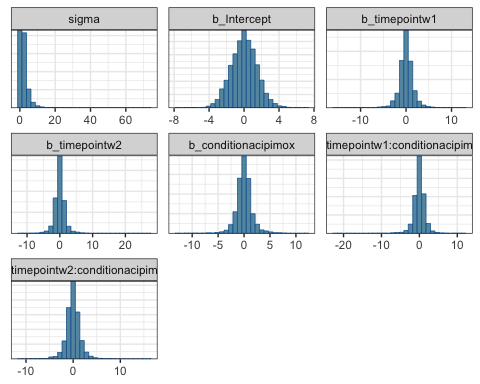  (a) Prior model predictions \| \| --- \| | \| 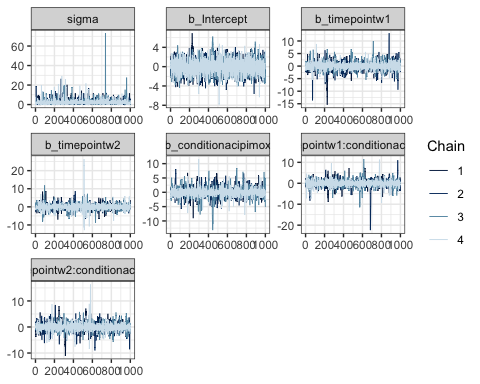  (b) Prior MCMC Traceplots \| \| --- \| |
| --- | --- | --- | --- |

Figure 3: Prior convergence checks

The histogram suggests samples in line with our expectations and the MCMC traceplots look like “hairy catepillars” as we’d hope if the MCMC process is effectively sampling the parameter space.

Next we examine the prior predictive density using 2000 draws from the prior predictive distribution. This plot shows estimates for western blot signal from the model but in the absence of data.

pp_check(prior_pred, ndraws = 2000, prefix = "ppd",
 type = "stat") +
 labs(title = paste("Prior predictive distribution for ", poi, sep = ""))

`stat_bin()` using `bins = 30`. Pick better value with `binwidth`.

| 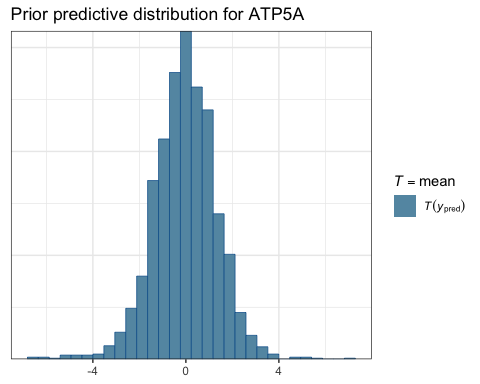  Figure 4: Prior predictive density |
| --- |

Overall we are satisfied with these checks and proceed to modeling.

**Bayesian modeling**

In the code below we use the brms package to run the model with the priors and data defined above.

rnd_int_mod <- model_data |>
 brm(formula = c_value ~ timepoint * condition + (1|subject),
 prior = rnd_int_priors,
 cores = 4,
 chains = 4,
 iter = 5000,
 stanvars=stanvars,
 seed = 7)

**Prior robustness check**

It is important that we check the robustness of the model in the face changes to the prior. Kallioinen et al present a method for carrying this out by power scaling priors (see [here](http://arxiv.org/abs/2107.14054)). This approach is implemented in the priorsense package.

chk <- powerscale_sensitivity(rnd_int_mod)$sensitivity

Loading required namespace: testthat

chk[,1:4] |>
 gt() |>
 fmt_number(decimals = 2) |>
 tab_header(paste("WB ", poi, " Prior robustness check", sep = ""))

Table 1: WB ATP5A Prior robustness check

| variable | prior | likelihood | diagnosis |
| --- | --- | --- | --- |
| b_Intercept | 0.00 | 0.07 | - |
| b_timepointw1 | 0.00 | 0.17 | - |
| b_timepointw2 | 0.00 | 0.14 | - |
| b_conditionacipimox | 0.01 | 0.06 | - |
| b_timepointw1:conditionacipimox | 0.01 | 0.16 | - |
| b_timepointw2:conditionacipimox | 0.01 | 0.15 | - |
| sd_subject__Intercept | 0.00 | 0.40 | - |
| sigma | 0.00 | 0.75 | - |
| Intercept | 0.00 | 0.02 | - |
| r_subject[RJ001,Intercept] | 0.00 | 0.17 | - |
| r_subject[RJ0010,Intercept] | 0.00 | 0.22 | - |
| r_subject[RJ0011,Intercept] | 0.00 | 0.13 | - |
| r_subject[RJ0012,Intercept] | 0.00 | 0.27 | - |
| r_subject[RJ0013,Intercept] | 0.00 | 0.05 | - |
| r_subject[RJ0014,Intercept] | 0.00 | 0.09 | - |
| r_subject[RJ0015,Intercept] | 0.00 | 0.22 | - |
| r_subject[RJ0016,Intercept] | 0.00 | 0.10 | - |
| r_subject[RJ0017,Intercept] | 0.00 | 0.11 | - |
| r_subject[RJ0018,Intercept] | 0.00 | 0.34 | - |
| r_subject[RJ0019,Intercept] | 0.00 | 0.19 | - |
| r_subject[RJ002,Intercept] | 0.00 | 0.16 | - |
| r_subject[RJ003,Intercept] | 0.00 | 0.13 | - |
| r_subject[RJ005,Intercept] | 0.00 | 0.48 | - |
| r_subject[RJ006,Intercept] | 0.00 | 0.08 | - |
| r_subject[RJ007,Intercept] | 0.00 | 0.22 | - |
| r_subject[RJ008,Intercept] | 0.00 | 0.10 | - |
| r_subject[RJ009,Intercept] | 0.00 | 0.13 | - |

The lack of any indicators (e.g. “prior-data conflict” or “weak likelihood”) in the diagnosis column of the chk object means we are happy that our prior is reasonably robust.

**Explore the model**

Next, we generate some plots to check the results of our model. First, we examine MCMC traceplots as we did above for the prior predictive checks.

# model sampling check
mcmc_hist(rnd_int_mod, regex_pars = "^b_", pars = "sigma")

`stat_bin()` using `bins = 30`. Pick better value with `binwidth`.

mcmc_trace(rnd_int_mod, regex_pars = "^b_", pars = "sigma")

| \| 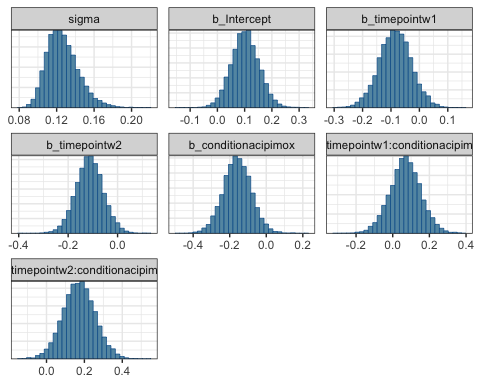  (a) Posterior model predictions \| \| --- \| | \| 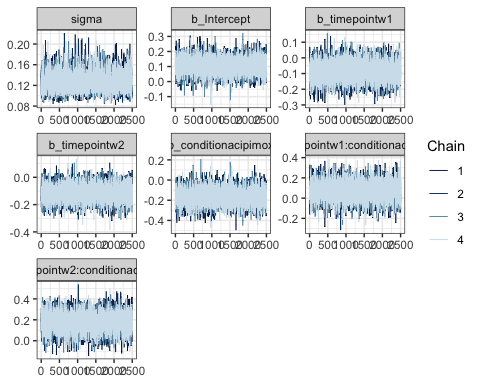  (b) Posterior MCMC Traceplots \| \| --- \| |
| --- | --- | --- | --- |

Figure 5: Prior convergence checks

These plots suggest the model has converged well.

Next we carry out posterior predictive checks to examine how well the model has fit the data.

# get yrep data
yrep <- brms::posterior_predict(rnd_int_mod, ndraws = 5000)
# plots of posteriors vs predicted
# timepoint
bayesplot::ppc_stat_grouped(model_data$c_value, yrep, stat = "mean", group = model_data$timepoint)

`stat_bin()` using `bins = 30`. Pick better value with `binwidth`.

pp_check(rnd_int_mod, ndraws = 10, group = "timepoint", type = "dens_overlay_grouped")

# condition
bayesplot::ppc_stat_grouped(model_data$c_value, yrep, stat = "mean", group = model_data$condition)

`stat_bin()` using `bins = 30`. Pick better value with `binwidth`.

pp_check(rnd_int_mod, ndraws = 10, group = "condition", type = "dens_overlay_grouped")

| \| 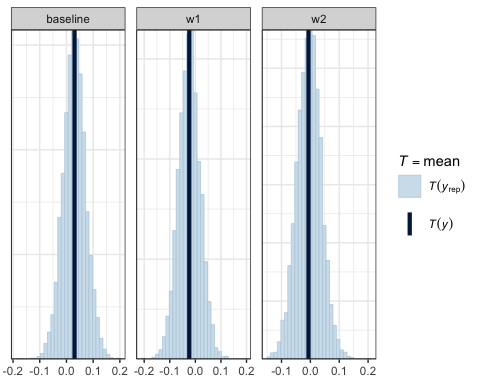  (a) Posterior predictions for timepoint - histogram \| \| --- \| | \| 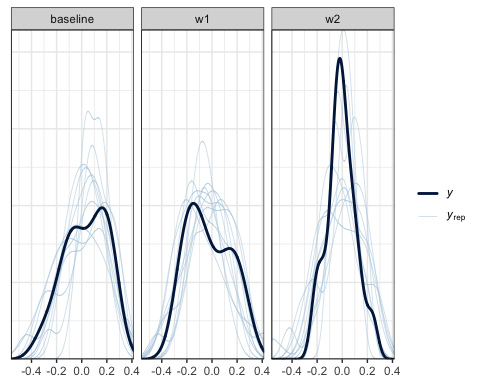  (b) Posterior predictions for timepoint - kernel density \| \| --- \| |
| --- | --- | --- | --- |

| \| 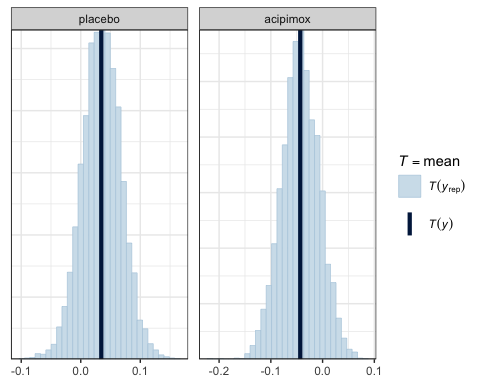  (c) Posterior predictions for condition - histogram \| \| --- \| | \| 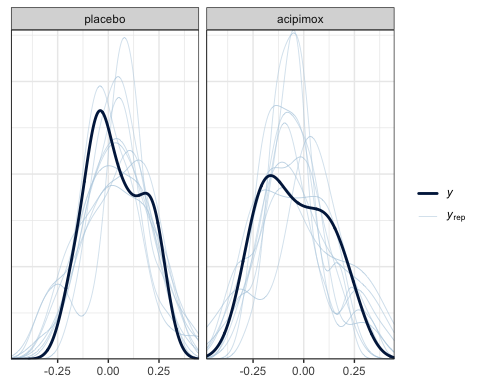  (d) Posterior predictions for condition - kernel density \| \| --- \| |
| --- | --- | --- | --- |

Figure 6: Posterior predictive check

These plots show the result of posterior predictive checking (PPC). The idea behind posterior predictive checking is simple: if a model is a good fit we should be able to use it to generate data that resemble the data that we observed (see [here](https://rss.onlinelibrary.wiley.com/doi/abs/10.1111/rssa.12378)). For both the main effects of timepoint and condition the histogram PPC plots show the mean of the data as a dark vertical line and the histogram shows the distribution of means we would predict from our model. In both cases the data mean lies in the middle of the posterior predictive distribution; this is exactly what we would expect. The density plots show different realisations of data according to the posterior distribution. The density plot for the actual data is shown as the darker line. These plots suggest reasonable posterior fit.

**Model parameters**

We can use packages from the [easystats](https://easystats.github.io/easystats/) meta-package to extract & tabulate the model coefficients.

# model parameters (easystats parameters pkg)
parameters(rnd_int_mod, effects = "fixed") |>
 gt() |>
 tab_header(title = poi) |>
 fmt_number(decimal = 3) |>
 cols_hide(c("Component", "CI")) |>
 tab_header(paste(poi, " Model coefficients", sep = ""))

Table 1: ATP5A Model coefficients

| Parameter | Median | CI_low | CI_high | pd | Rhat | ESS |
| --- | --- | --- | --- | --- | --- | --- |
| b_Intercept | 0.102 | 0.003 | 0.204 | 0.979 | 1.000 | 5,450.996 |
| b_timepointw1 | -0.085 | -0.200 | 0.029 | 0.931 | 1.000 | 7,393.579 |
| b_timepointw2 | -0.113 | -0.223 | -0.001 | 0.976 | 1.000 | 7,453.162 |
| b_conditionacipimox | -0.159 | -0.312 | -0.006 | 0.979 | 1.000 | 5,169.800 |
| b_timepointw1:conditionacipimox | 0.070 | -0.100 | 0.236 | 0.796 | 1.001 | 7,085.885 |
| b_timepointw2:conditionacipimox | 0.172 | 0.005 | 0.343 | 0.978 | 1.000 | 7,092.057 |
| sigma | 0.125 | 0.099 | 0.166 | 1.000 | 1.001 | 3,774.907 |

The (Intercept) coefficient is the mean value at baseline in the placebo group; this has to be interpreted in the context of the mean centered data. The timepointw1 and timepointw2 coefficients are the changes from baseline in the placebo group at timepoints W1 and W2. The condition acipimox coefficient is the difference between placebo and acipimox conditions at baseline. The timepointw1:conditionacipimox & timepointw2:conditionacipimox coefficients are the differences between baseline and W1 and W2 (respectively) in the acipimox condition. The Rhat & ESS are model diagnostics and suggest we have a model that has converged (Rhat) and a high number of posterior samples (ESS) (see [here](http://arxiv.org/abs/2011.01808)).

Instead of examining differences across conditions we can extract marginal means for each condition. These are the model estimated average values of the c_value variable at each condition.

estimate_means(rnd_int_mod, at = c( "condition", "timepoint"), test = NULL) |>
 gt() |>
 tab_header(title = poi) |>
 fmt_number(decimal = 3) |>
 tab_header("Marginal means")

Table 1: Marginal means

| condition | timepoint | Mean | Median.1 | CI_low | CI_high |
| --- | --- | --- | --- | --- | --- |
| placebo | baseline | 0.102 | 0.102 | 0.003 | 0.204 |
| acipimox | baseline | -0.056 | -0.056 | -0.167 | 0.056 |
| placebo | w1 | 0.017 | 0.017 | -0.084 | 0.119 |
| acipimox | w1 | -0.073 | -0.073 | -0.186 | 0.041 |
| placebo | w2 | -0.012 | -0.012 | -0.110 | 0.089 |
| acipimox | w2 | 0.002 | 0.002 | -0.107 | 0.114 |

Perhaps most informatively we can extract contrasts for the difference between each timepoint in each condition.

estimate_contrasts(rnd_int_mod, contrast = "timepoint", at = "condition", test = "pd") |>
 gt() |>
 tab_header(title = poi) |>
 fmt_number(decimal = 3) |>
 tab_header("Condition contrasts")

Table 1: Condition contrasts

| Level1 | Level2 | condition | Difference | CI_low | CI_high | pd |
| --- | --- | --- | --- | --- | --- | --- |
| baseline | w1 | placebo | 0.085 | -0.029 | 0.200 | 0.931 |
| baseline | w2 | placebo | 0.113 | 0.001 | 0.223 | 0.976 |
| w1 | w2 | placebo | 0.029 | -0.085 | 0.142 | 0.694 |
| baseline | w1 | acipimox | 0.015 | -0.110 | 0.143 | 0.596 |
| baseline | w2 | acipimox | -0.059 | -0.183 | 0.070 | 0.823 |
| w1 | w2 | acipimox | -0.075 | -0.198 | 0.049 | 0.887 |

This contrast table includes the “probability of direction” (pd column). This tells us the probability (from the posterior distribution) of the direction of a given contrast.

**Examining hypotheses**

We can also examine specific hypotheses by calculating area under the posterior distribution for a given hypothesis. Below we test hypotheses for baseline vs W1 and baseline vs W2 in the placebo and acipimox conditions being equal to zero.

# test hypotheses & get posterior draws for hypotheses
hyp <- c("timepointw1 = 0", "timepointw2 = 0",
 "timepointw1 + timepointw1:conditionacipimox = 0",
 "timepointw2 + timepointw2:conditionacipimox = 0")

# get hypothesis posterior draws
posts <- hypothesis(rnd_int_mod, hyp)$samples
# test direction of effect
hyp_tests <- bayestestR::describe_posterior(
 posts,
 test = c("p_direction"),
 centrality = "median")
hyp_tests$Parameter <- c("Placebo:T1-baseline",
 "Placebo:T2-baseline",
 "Acipimox:T1-baseline",
 "Acipimox:T2-baseline")
hyp_tests |>
 gt() |>
 fmt_number(decimal = 2) |>
 tab_header("Probability of direction for defined hypotheses")

Table 1: Probability of direction for defined hypotheses

| Parameter | Median | CI | CI_low | CI_high | pd |
| --- | --- | --- | --- | --- | --- |
| Placebo:T1-baseline | -0.08 | 0.95 | -0.20 | 0.03 | 0.93 |
| Placebo:T2-baseline | -0.11 | 0.95 | -0.22 | 0.00 | 0.98 |
| Acipimox:T1-baseline | -0.02 | 0.95 | -0.14 | 0.11 | 0.60 |
| Acipimox:T2-baseline | 0.06 | 0.95 | -0.07 | 0.18 | 0.82 |

We then extract the posterior data for these hypotheses and plot those with colour indicating the probability of direction.

# plot
# hypothesis labels
ys <- c(bquote("T1-baseline"["plac"]),
 bquote("T2-baseline"["plac"]),
 bquote("T1-baseline"["aci"]),
 bquote("T2-baseline"["aci"]))

pl_ttl <- paste("Probability of Direction: ", poi, sep = "")


# plot hypothesis posteriors i.e. prob dir; add prob info
plt_ttl <- paste("Probability of Direction: ", poi, sep = "")
posts |>
 pivot_longer(cols = everything(), names_to = "cond", values_to = "draws") |>
 ggplot(aes(x = draws, y = rev(cond), fill = after_stat(x < 0))) +
 # see https://mjskay.github.io/ggdist/articles/slabinterval.html#highlighting-and-other-combinations
 # for colouring
 geom_vline(xintercept = 0, linetype = "dashed", linewidth = 1) +
 # useful for appearance of stat_halfeye
 stat_halfeye(.width = 0.95, slab_linewidth = 0.5, slab_color = "black") +
 scale_y_discrete(name = "",
 breaks = c("H1", "H2", "H3", "H4"),
 labels = rev(ys)) +
 scale_fill_manual(name = "Probability of direction: ", labels = c("Positive", "Negative"),
 values = clrs) +
 labs(title = plt_ttl,
 x = "Possible parameter values") +
 theme(legend.position = "bottom",
 axis.text.x = element_text(size=10),
 axis.text.y = element_text(size=12))

| 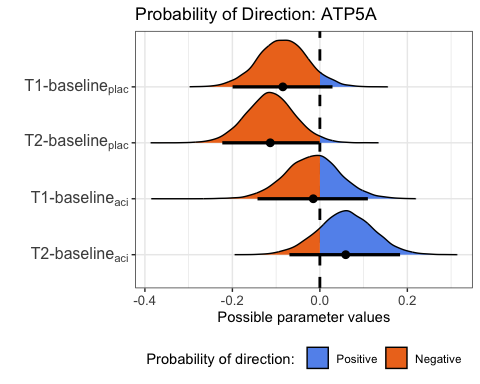  Figure 7: Probability of direction for study effects |
| --- |

**Frequentist Analysis**

We can also run a frequentist hierarchical model using the lme4 package to compare the model coefficients.

fmod <- lmer(c_value ~ timepoint * condition + (1|subject), data = model_data)

# extract model coefs
parameters::parameters(fmod) |>
 gt() |>
 fmt_number(decimal = 3) |>
 cols_hide(c("Effects", "Group", "CI", "df_error")) |>
 tab_header(paste(poi, " Frequentist model results", sep = ""))

Table 1: ATP5A Frequentist model results

| Parameter | Coefficient | SE | CI_low | CI_high | t | p |
| --- | --- | --- | --- | --- | --- | --- |
| (Intercept) | 0.101 | 0.047 | 0.006 | 0.196 | 2.142 | 0.038 |
| timepointw1 | -0.085 | 0.054 | -0.193 | 0.023 | -1.589 | 0.119 |
| timepointw2 | -0.114 | 0.054 | -0.222 | -0.006 | -2.132 | 0.038 |
| conditionacipimox | -0.159 | 0.071 | -0.302 | -0.017 | -2.250 | 0.029 |
| timepointw1:conditionacipimox | 0.070 | 0.080 | -0.092 | 0.232 | 0.871 | 0.388 |
| timepointw2:conditionacipimox | 0.175 | 0.080 | 0.013 | 0.336 | 2.170 | 0.035 |
| SD (Intercept) | 0.089 | 0.026 | 0.050 | 0.158 | NA | NA |
| SD (Observations) | 0.120 | 0.015 | 0.094 | 0.153 | NA | NA |

# bayesian model for comparison
parameters::parameters(rnd_int_mod) |>
 gt() |>
 fmt_number(decimal = 3) |>
 cols_hide(c("Component", "CI")) |>
 tab_header(paste(poi, " Bayesian model results", sep = ""))

Table 1: ATP5A Bayesian model results

| Parameter | Median | CI_low | CI_high | pd | Rhat | ESS |
| --- | --- | --- | --- | --- | --- | --- |
| b_Intercept | 0.102 | 0.003 | 0.204 | 0.979 | 1.000 | 5,450.996 |
| b_timepointw1 | -0.085 | -0.200 | 0.029 | 0.931 | 1.000 | 7,393.579 |
| b_timepointw2 | -0.113 | -0.223 | -0.001 | 0.976 | 1.000 | 7,453.162 |
| b_conditionacipimox | -0.159 | -0.312 | -0.006 | 0.979 | 1.000 | 5,169.800 |
| b_timepointw1:conditionacipimox | 0.070 | -0.100 | 0.236 | 0.796 | 1.001 | 7,085.885 |
| b_timepointw2:conditionacipimox | 0.172 | 0.005 | 0.343 | 0.978 | 1.000 | 7,092.057 |
| sigma | 0.125 | 0.099 | 0.166 | 1.000 | 1.001 | 3,774.907 |

The predictions from the frequentist model are plotted along with the data below.

# plot
plot(estimate_means(fmod)) +
 scale_colour_manual(values = clrs,
 name = "condition", labels = c("Placebo", "Acipimox")) +
 labs(title = paste("WB Frequentist Model: ", poi, sep = ""),
 x = "Timepoint", y = "AU")

We selected `at = c("timepoint", "condition")`.

| 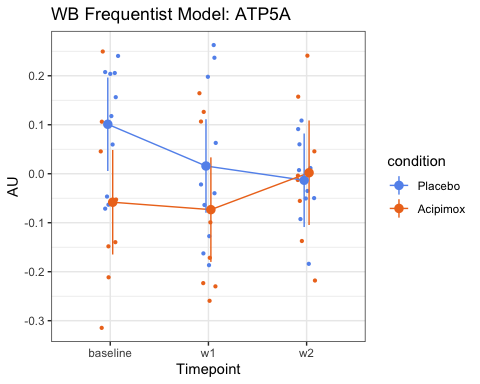  Figure 8: Frequentist model predictions (with data) |
| --- |

The frequentist and Bayesian models are in agreement in terms of model coefficients. Note that we are able to get a richer inference (e.g probability of direction) from the Bayesian model.

**Citrate synthase Bayesian analysis**

**Introduction**

This study examined the effects of acipimox, an NAD+ precursor on mitochondrial function. There were two treatment groups: placebo (n = 10) and acipimox (n = 8). Participants had muscle biopsies at baseline and at 1- & 2-weeks following placebo/acipimox treatment and citrate synthase activity was assessed in these muscle biopsies.

In this analysis we will use a Bayesian hierarchical approach to model the average change in citrate synthase activity over time in the placebo and acipimox groups.

**Packages & environment**

First, we load the packages we will need and make sure we start with clean environment.

library(brms) # bayesian modeling
library(bayesplot) # diagnostic plots etc
library(tidyverse) # data wrangling
library(gridExtra) # multiplot figures
library(tidybayes) # plots; distributions
library(easystats) # model summaries
library(priorsense) # for prior robustness checking; remotes::install_github("n-kall/priorsense")
library(gt) # tables
library(lme4) # frequentist analysis
theme_set(theme_bw())

# start clean
rm(list = ls())

Next, we load the data. The data is in long format (each row represents one measure) & contains the following variables:

- subject - subject ID
- timepoint - the timepoint (baseline, w1 & w2)
- condition - the condition (placebo, acipimox)
- value - measured citrate synthase activity in µM/min/µL

We first convert the subject, timepoint and condition variables to factors & reverse the order of the condition variable so placebo is the reference category for plotting and modeling.

cs_data <- read_csv('data/CS_long_data.csv')
# values are in µM/min/µL
# factors from subj, condition, timepoint
cs_data <- cs_data |>
 mutate(across(where(is.character), as.factor))
# reverse order of placebo and acipimox for plots & model etc
cs_data <- cs_data |>
 mutate(condition = fct_rev(condition))

**Exploratory plots**

Next, we create an exploratory plot.

# set plot colours
clrs <- c("cornflowerblue", "chocolate2")

# CS activity over intervention b -> w1 -> w2
ylab <- bquote('CS activity ('*mu*'M/min/'*mu*'L)') # yaxis label; * removes spaces from bquote
cs_data |>
 summarise(mn = mean(value), .by = condition:timepoint) |>
 ggplot(aes(timepoint, mn, colour = condition)) +
 geom_point(position = position_jitterdodge(jitter.width = 0.05), size = 3) +
 geom_line(aes(group = condition), position = position_jitterdodge(jitter.width = 0.05)) +
 geom_point(data = cs_data, aes(timepoint, value, colour = condition),
 position = position_jitterdodge(jitter.width = 0.05),
 alpha = 0.4) +
 # x labels
 scale_x_discrete(labels=c("baseline" = "B",
 "w1" = "W1",
 "w2" = "W2")) +
 # legend labels & plot colours
 scale_colour_manual(values = clrs,
 name = "Condition", labels = c("Placebo", "Acipimox")) +
 labs(x = 'Timepoint', y = ylab)

| 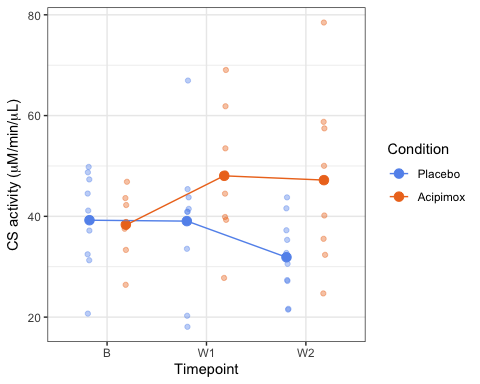  Figure 1: Exploratory figure |
| --- |

**Statistical modeling**

We will build a Bayesian hierarchical (mixed) model to assess changes in citrate synthase activity. The “random” term in these models will be the subject variable to account for repeated observations from each participant across the study timeline.

We will mean center the data as this makes the later specification of priors easier.

# summary stats
model_data <- cs_data
model_data |>
 summarise(Mean = mean(value), Std = sd(value), .by =c(timepoint, condition)) |>
 gt() |>
 fmt_number(decimals = 2) |>
 cols_align(columns = 'timepoint',
 align = c("left"))

| timepoint | condition | Mean | Std |
| --- | --- | --- | --- |
| baseline | acipimox | 38.36 | 6.37 |
| w1 | acipimox | 48.05 | 13.25 |
| w2 | acipimox | 47.19 | 17.47 |
| baseline | placebo | 39.24 | 9.17 |
| w1 | placebo | 39.05 | 13.65 |
| w2 | placebo | 31.89 | 7.70 |

# center data
model_data <- model_data |>
 mutate(c_value = value - mean(value))

**Setting priors**

The model we will estimate is:

$$y_{i}=\left( \beta_{0}+\sigma_{i} \right)+\beta_{1}\times time_{i}+\beta_{2}\times cond_{i}+\beta_{3}\times\left( time_{i}\times cond_{i} \right)+\epsilon_{i}$$

where $\sigma$ is the random intercept variance and $\epsilon$ is the residual variance.

We need to set priors on each of the model parameters we are estimating. Here we estimate an overall intercept ($\beta_{0}$), a main effect of treatment ($\beta_{1}$), a main effect of time ($\beta_{2}$), the interaction between timepoint & condition ($\beta_{3}$), the variation in subject specific intercepts ($\sigma$) and the residual variation ($\epsilon$).

**Intercept prior**

The overall intercept is expected value when all other coefficients in a linear model are zero. In our context this is the mean of the placebo treatment at baseline. The data have been mean centered so we will set the prior mean at zero. A sensible prior for the overall intercept would be $\sim N\left( 0, 4\times sd of the data \right)$. The mean is our most plausible value for the overall intercept but it is plausible the overall intercept could vary by the four times amount of variability we see in the data overall. Whilst this is wide from a biological perspective it provides much more regularisation than the flat prior implied by the usual frequentist tests.

We use the stanvar() function to make the user derived values available to the Stan language which will do the MCMC sampling for us via the brms package.

# intercept prior
int_prior_sd <- model_data |>
 summarise(sds = ceiling(sd(c_value))*4) |>
 pull()

# sd prior
sd_prior <- model_data |>
 summarise(sds = ceiling(sd(c_value))*2) |>
 pull()

# set prior; use stanvars
stanvars <- stanvar(int_prior_sd, name="int_prior_sd") +
 stanvar(sd_prior, name = "sd_prior")

**Slope and variance priors**

Next, we have to set priors for the $\beta$ (slope) coefficients in our model, the variance in the intercepts ($\sigma$) and the residual variance ($\epsilon$). For the slope coefficients we will use a student t prior with 3 degrees of freedom, a mean of zero (slopes could be positive or negative) and a standard deviation four times the standard deviation of the data overall (as in the intercept prior).

For the intercept variation and residual variation, we use a $t$ distribution, again wide on the scale of the data. We limit the plausible values to only positive values (lb = 0; lower bound = 0) because variances cannot be negative. These variance priors suggest that the low variances are most likely but allow room for larger variance if the data suggests that.

rnd_int_priors <- c(prior(normal(0, int_prior_sd), class = Intercept), # intercept
 prior(student_t(3, 0, int_prior_sd), class = b), # slope; wide on scale of data
 prior(student_t(3, 0, sd_prior), class = sd, lb = 0), # random intercept sd; sd set on scale of data
 prior(student_t(3, 0, sd_prior), class = sigma, lb = 0)) # residual variance) # residual var; sd set on scale of data

We can plot these priors to examine plausible values for each of the parameters we are trying to estimate.

# plot priors
xlab <- bquote('CS activity ('*mu*'M/min/'*mu*'L)')
rnd_int_priors |>
 parse_dist() |>
 ggplot(aes(y = paste(class, "~", format(.dist_obj)), xdist = .dist_obj)) +
 stat_halfeye(.width = 0.95, limits = c(-100,100),
 slab_linewidth = 0.5,
 slab_color = 'black',
 slab_fill = 'cadetblue') +
 labs(title = "Priors",
 x = xlab,
 y = NULL)

| 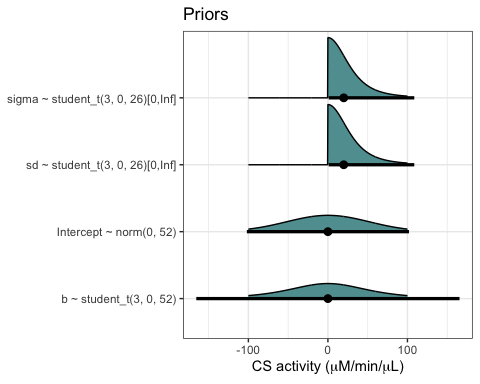  Figure 2: Priors used in the analysis |
| --- |

Next, we carry out prior predictive checks. Prior predictive checks involve simulating data from the model as defined by the priors *in the absence of data* and examining how compatible those simulations are with values we might expect. Here the priors have been chosen to be wide on the scale of the data and so our simulated data should also be more variable (wider) than the data we have but not wholly unreasonable.

# prior predictive check
prior_pred <- model_data |>
 brm(formula = c_value ~ timepoint * condition + (1|subject),
 prior = rnd_int_priors,
 family = gaussian(),
 cores = 4,
 iter = 2000,
 sample_prior = "only",
 stanvars=stanvars)

We examine the coefficients from the prior predictive model.

# check
parameters(prior_pred, effects = "fixed") |>
 gt() |>
 fmt_number(decimal = 3) |>
 cols_hide(c("Component", "CI")) |>
 tab_header("CS Prior predicitve model coefficients")

Table 1: CS Prior predicitve model coefficients

| Parameter | Median | CI_low | CI_high | pd | Rhat | ESS |
| --- | --- | --- | --- | --- | --- | --- |
| b_Intercept | 0.545 | -158.996 | 162.636 | 0.503 | 1.003 | 2,045.621 |
| b_timepointw1 | 0.857 | -169.012 | 169.464 | 0.506 | 1.002 | 2,188.914 |
| b_timepointw2 | -0.400 | -153.101 | 148.198 | 0.503 | 1.002 | 2,139.331 |
| b_conditionacipimox | 0.055 | -168.179 | 159.670 | 0.501 | 1.005 | 1,088.368 |
| b_timepointw1:conditionacipimox | -0.414 | -178.239 | 162.714 | 0.502 | 1.012 | 283.542 |
| b_timepointw2:conditionacipimox | 0.900 | -146.932 | 144.681 | 0.509 | 1.002 | 2,578.278 |
| sigma | 19.576 | 0.871 | 108.225 | 1.000 | 0.999 | 5,132.869 |

Next, we generate plots to examine how the MCMC sampling process for the model has converged and the distribution of the model predictions without any data input. First, we examine a histogram of the prior model predictions and MCMC traceplots.

# model sampling check
mcmc_hist(prior_pred, regex_pars = "^b_", pars = "sigma")

`stat_bin()` using `bins = 30`. Pick better value with `binwidth`.

mcmc_trace(prior_pred, regex_pars = "^b_", pars = "sigma")

| \| 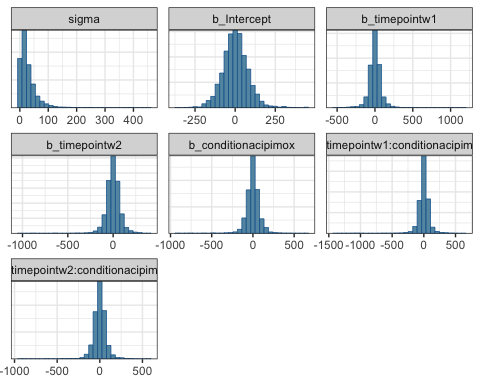  (a) Prior model predictions \| \| --- \| | \| 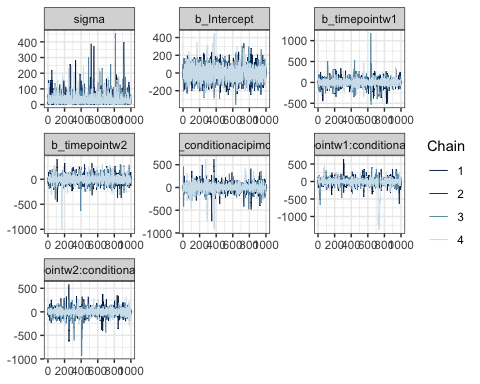  (b) Prior MCMC Traceplots \| \| --- \| |
| --- | --- | --- | --- |

Figure 3: Prior convergence checks

The histogram suggests samples in line with our expectations and the MCMC traceplots look like “hairy catepillars” as we’d hope if the MCMC process is effectively sampling the parameter space.

Next, we examine the prior predictive density using 2000 draws from the prior predictive distribution. This plot shows estimates for CS activity from the model but in the absence of data.

xlab <- bquote('Centered CS activity ('*mu*'M/min/'*mu*'L)')
pp_check(prior_pred, ndraws = 2000, prefix = "ppd",
 type = "stat") +
 labs(title = "Prior predictive distribution for centered CS activity",
 x = xlab)

`stat_bin()` using `bins = 30`. Pick better value with `binwidth`.

| 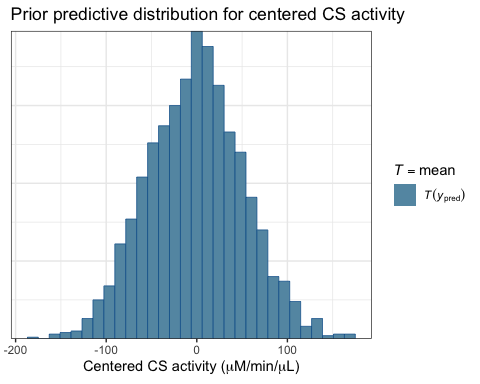  Figure 4: Prior predictive density |
| --- |

Overall, we are satisfied with these checks and proceed to modeling.

**Bayesian modeling**

In the code below we use the brms package to run the model with the priors and data defined above.

Rnd_int_mod <- model_data |>
 brm(formula = c_value ~ timepoint * condition + (1|subject),
 prior = rnd_int_priors,
 cores = 4,
 chains = 4,
 iter = 5000,
 stanvars=stanvars,
 seed = 7)

**Prior robustness check**

It is important that we check the robustness of the model in the face changes to the prior. Kallioinen et al present a method for carrying this out by power scaling priors (see [here](http://arxiv.org/abs/2107.14054)). This approach is implemented in the priorsense package.

chk <- powerscale_sensitivity(rnd_int_mod)$sensitivity

Loading required namespace: testthat

chk[,1:4] |>
 gt() |>
 fmt_number(decimals = 2) |>
 tab_header("CS activity Prior robustness check")

Table 1: CS activity Prior robustness check

| variable | prior | likelihood | diagnosis |
| --- | --- | --- | --- |
| b_Intercept | 0.01 | 0.07 | - |
| b_timepointw1 | 0.01 | 0.16 | - |
| b_timepointw2 | 0.01 | 0.14 | - |
| b_conditionacipimox | 0.01 | 0.08 | - |
| b_timepointw1:conditionacipimox | 0.01 | 0.16 | - |
| b_timepointw2:conditionacipimox | 0.01 | 0.16 | - |
| sd_subject__Intercept | 0.01 | 0.40 | - |
| sigma | 0.01 | 0.66 | - |
| Intercept | 0.00 | 0.02 | - |
| r_subject[RJ001,Intercept] | 0.00 | 0.05 | - |
| r_subject[RJ0010,Intercept] | 0.00 | 0.15 | - |
| r_subject[RJ0011,Intercept] | 0.00 | 0.09 | - |
| r_subject[RJ0012,Intercept] | 0.00 | 0.22 | - |
| r_subject[RJ0013,Intercept] | 0.00 | 0.29 | - |
| r_subject[RJ0014,Intercept] | 0.00 | 0.05 | - |
| r_subject[RJ0015,Intercept] | 0.00 | 0.30 | - |
| r_subject[RJ0016,Intercept] | 0.00 | 0.25 | - |
| r_subject[RJ0017,Intercept] | 0.00 | 0.28 | - |
| r_subject[RJ0018,Intercept] | 0.00 | 0.22 | - |
| r_subject[RJ0019,Intercept] | 0.00 | 0.39 | - |
| r_subject[RJ002,Intercept] | 0.00 | 0.15 | - |
| r_subject[RJ003,Intercept] | 0.00 | 0.11 | - |
| r_subject[RJ005,Intercept] | 0.00 | 0.23 | - |
| r_subject[RJ006,Intercept] | 0.00 | 0.04 | - |
| r_subject[RJ007,Intercept] | 0.00 | 0.07 | - |
| r_subject[RJ008,Intercept] | 0.00 | 0.12 | - |
| r_subject[RJ009,Intercept] | 0.00 | 0.11 | - |

The lack of any indicators (e.g. “prior-data conflict” or “weak likelihood”) in the diagnosis column of the chk object means we are happy that our prior is robust.

**Explore the model**

Next, we generate some plots to check the results of our model. First, we examine the overall predictions MCMC traceplots as we did above for the prior predictive checks.

# model sampling check
mcmc_hist(rnd_int_mod, regex_pars = “^b_”, pars = “sigma”)

`stat_bin()` using `bins = 30`. Pick better value with `binwidth`.

Mcmc_trace(rnd_int_mod, regex_pars = “^b_”, pars = “sigma”)

| \| 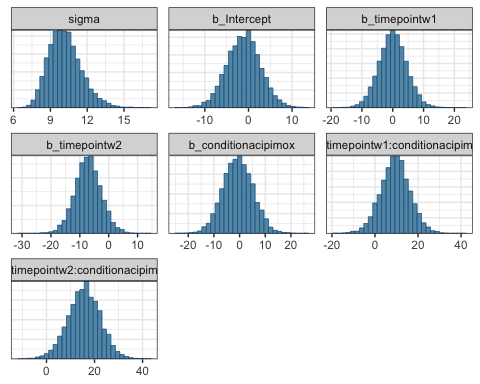  (a) Posterior model predictions \| \| --- \| | \| 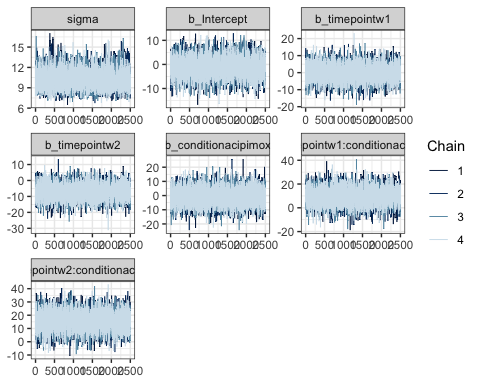  (b) Posterior MCMC Traceplots \| \| --- \| |
| --- | --- | --- | --- |

Figure 5: Prior convergence checks

These plots suggest the model has converged well.

Next, we carry out posterior predictive checks to examine how well the model has fit the data.

# get yrep data
yrep <- brms::posterior_predict(rnd_int_mod, ndraws = 5000)
# plots of posteriors vs predicted
# timepoint
bayesplot::ppc_stat_grouped(model_data$c_value, yrep, stat = "mean", group = model_data$timepoint)

`stat_bin()` using `bins = 30`. Pick better value with `binwidth`.

pp_check(rnd_int_mod, ndraws = 10, group = "timepoint", type = "dens_overlay_grouped")

# condition
bayesplot::ppc_stat_grouped(model_data$c_value, yrep, stat = "mean", group = model_data$condition)

`stat_bin()` using `bins = 30`. Pick better value with `binwidth`.

pp_check(rnd_int_mod, ndraws = 10, group = "condition", type = "dens_overlay_grouped")

| \| 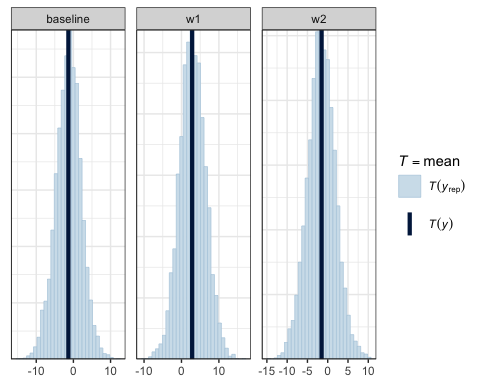  (a) Posterior predictions for timepoint - histogram \| \| --- \| | \| 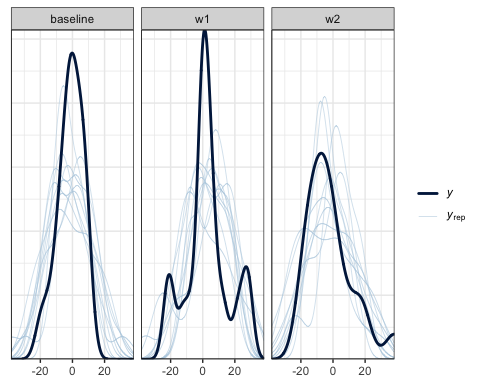  (b) Posterior predictions for timepoint - kernel density \| \| --- \| |
| --- | --- | --- | --- |

| \| 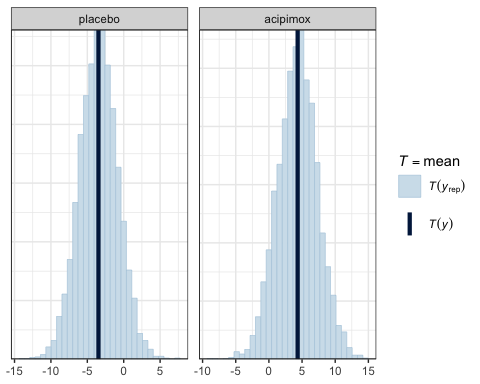  (c) Posterior predictions for condition - histogram \| \| --- \| | \| 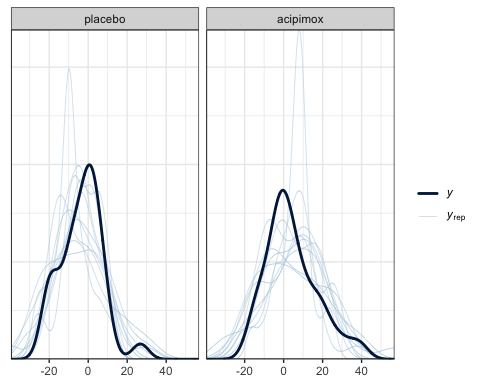  (d) Posterior predictions for condition - kernel density \| \| --- \| |
| --- | --- | --- | --- |

Figure 6: Posterior predictive check

These plots show the result of posterior predictive checking (PPC). The idea behind posterior predictive checking is simple: if a model is a good fit we should be able to use it to generate data that resemble the data that we observed (see [here](https://rss.onlinelibrary.wiley.com/doi/abs/10.1111/rssa.12378)). For both the main effects of timepoint and condition the histogram PPC plots show the mean of the data as a dark vertical line and the histogram shows the distribution of means we would predict from our model. In both cases the data mean lies in the middle of the posterior predictive distribution; this is exactly what we would expect. The density plots show different realisations of data according to the posterior distribution. The density plot for the actual data is shown as the darker line. These plots suggest reasonably good posterior fit.

**Model parameters**

We can use packages from the [easystats](https://easystats.github.io/easystats/) meta-package to extract & tabulate the model coefficients.

# model parameters (easystats parameters pkg)
parameters(rnd_int_mod, effects = "fixed") |>
 gt() |>
 fmt_number(decimal = 3) |>
 cols_hide(c("Component", "CI")) |>
 tab_header("CS activity model coefficients")

Table 1: CS activity model coefficients

| Parameter | Median | CI_low | CI_high | pd | Rhat | ESS |
| --- | --- | --- | --- | --- | --- | --- |
| b_Intercept | -1.036 | -8.699 | 6.581 | 0.608 | 1.000 | 6,016.164 |
| b_timepointw1 | 0.041 | -8.944 | 9.024 | 0.504 | 1.000 | 7,957.055 |
| b_timepointw2 | -7.181 | -16.145 | 1.865 | 0.944 | 1.000 | 7,427.161 |
| b_conditionacipimox | -0.676 | -11.878 | 10.682 | 0.547 | 1.000 | 6,050.306 |
| b_timepointw1:conditionacipimox | 9.519 | -4.072 | 22.913 | 0.919 | 1.000 | 7,213.620 |
| b_timepointw2:conditionacipimox | 15.798 | 2.198 | 28.757 | 0.988 | 1.001 | 7,205.233 |
| sigma | 10.092 | 7.951 | 13.211 | 1.000 | 1.001 | 3,633.210 |

The (Intercept) coefficient is the mean value at baseline in the placebo group; this has to be interpreted in the context of the mean centered data. The timepointw1 and timepointw2 coefficients are the changes from baseline in the placebo group at timepoints W1 and W2. The condition acipimox coefficient is the difference between placebo and acipimox conditions at baseline. The timepointw1:conditionacipimox & timepointw2:conditionacipimox coefficients are the differences between baseline and W1 and W2 (respectively) in the acipimox condition. The Rhat & ESS are model diagnostics and suggest we have a model that has converged (Rhat) and a high number of posterior samples (ESS) (see [here](http://arxiv.org/abs/2011.01808)).

Instead of examining differences across conditions we can extract marginal means for each condition. These are the model estimated average values of the centered (i.e. c_value) variable at each condition.

estimate_means(rnd_int_mod, at = c( "condition", "timepoint"), test = NULL) |>
 gt() |>
 fmt_number(decimal = 3) |>
 tab_header("CS activity marginal means")

Table 1: CS activity marginal means

| condition | timepoint | Mean | Median.1 | CI_low | CI_high |
| --- | --- | --- | --- | --- | --- |
| placebo | baseline | -1.036 | -1.036 | -8.699 | 6.581 |
| acipimox | baseline | -1.759 | -1.759 | -10.465 | 7.084 |
| placebo | w1 | -1.032 | -1.032 | -8.829 | 6.747 |
| acipimox | w1 | 7.759 | 7.759 | -0.945 | 16.398 |
| placebo | w2 | -8.282 | -8.282 | -15.951 | -0.444 |
| acipimox | w2 | 6.826 | 6.826 | -1.950 | 15.351 |

Perhaps most informatively we can extract contrasts for the difference between each timepoint in each condition.

estimate_contrasts(rnd_int_mod, contrast = "timepoint", at = "condition", test = "pd") |>
 gt() |>
 fmt_number(decimal = 3) |>
 tab_header("CS activity condition contrasts")

Table 1: CS activity condition contrasts

| Level1 | Level2 | condition | Difference | CI_low | CI_high | pd |
| --- | --- | --- | --- | --- | --- | --- |
| baseline | w1 | placebo | -0.041 | -9.024 | 8.944 | 0.504 |
| baseline | w2 | placebo | 7.181 | -1.865 | 16.145 | 0.944 |
| w1 | w2 | placebo | 7.192 | -1.724 | 16.089 | 0.944 |
| baseline | w1 | acipimox | -9.509 | -19.726 | 0.645 | 0.967 |
| baseline | w2 | acipimox | -8.567 | -18.573 | 1.755 | 0.951 |
| w1 | w2 | acipimox | 0.960 | -9.313 | 11.187 | 0.574 |

This contrast table includes the “probability of direction” (pd column). This tells us the probability (from the posterior distribution) of the direction of a given contrast.

**Examining hypotheses**

We can also examine specific hypotheses by calculating area under the posterior distribution for a given hypothesis. Below we test hypotheses for baseline vs W1 and baseline vs W2 in the placebo and acipimox conditions being equal to zero.

# test hypotheses & get posterior draws for hypotheses
hyp <- c("timepointw1 = 0", "timepointw2 = 0",
 "timepointw1 + timepointw1:conditionacipimox = 0",
 "timepointw2 + timepointw2:conditionacipimox = 0")

# get hypothesis posterior draws
posts <- hypothesis(rnd_int_mod, hyp)$samples
# test direction of effect
hyp_tests <- bayestestR::describe_posterior(
 posts,
 test = c("p_direction"),
 centrality = "median")
hyp_tests$Parameter <- c("Placebo:T1-baseline",
 "Placebo:T2-baseline",
 "Acipimox:T1-baseline",
 "Acipimox:T2-baseline")
hyp_tests |>
 gt() |>
 fmt_number(decimal = 2) |>
 tab_header("Probability of direction for defined hypotheses")

Table 1: Probability of direction for defined hypotheses

| Parameter | Median | CI | CI_low | CI_high | pd |
| --- | --- | --- | --- | --- | --- |
| Placebo:T1-baseline | 0.04 | 0.95 | -8.94 | 9.02 | 0.50 |
| Placebo:T2-baseline | -7.18 | 0.95 | -16.15 | 1.86 | 0.94 |
| Acipimox:T1-baseline | 9.51 | 0.95 | -0.65 | 19.73 | 0.97 |
| Acipimox:T2-baseline | 8.57 | 0.95 | -1.76 | 18.57 | 0.95 |

We then extract the posterior data for these hypotheses and plot those with colour indicating the probability of direction.

# plot
# hypothesis labels
ys <- c(bquote("T1-baseline"["plac"]),
 bquote("T2-baseline"["plac"]),
 bquote("T1-baseline"["aci"]),
 bquote("T2-baseline"["aci"]))

# plot hypothesis posteriors i.e. prob dir; add prob info
plt_ttl <- "Probability of Direction: CS activity"
posts |>
 pivot_longer(cols = everything(), names_to = "cond", values_to = "draws") |>
 ggplot(aes(x = draws, y = rev(cond), fill = after_stat(x < 0))) +
 # see https://mjskay.github.io/ggdist/articles/slabinterval.html#highlighting-and-other-combinations
 # for colouring
 geom_vline(xintercept = 0, linetype = "dashed", linewidth = 1) +
 # useful for appearance of stat_halfeye
 stat_halfeye(.width = 0.95, slab_linewidth = 0.5, slab_color = "black") +
 scale_y_discrete(name = "",
 breaks = c("H1", "H2", "H3", "H4"),
 labels = rev(ys)) +
 scale_fill_manual(name = "Probability of direction: ", labels = c("Positive", "Negative"),
 values = clrs) +
 labs(title = plt_ttl,
 x = "Possible parameter values") +
 theme(legend.position = "bottom",
 axis.text.x = element_text(size=10),
 axis.text.y = element_text(size=12))

| 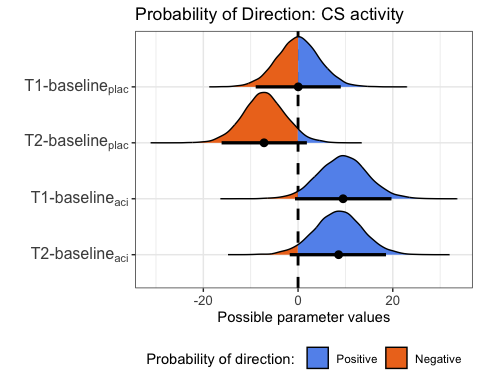  Figure 7: Probability of direction for study effects |
| --- |

**Frequentist Analysis**

We can also run a frequentist hierarchical model using the lme4 package to compare the model coefficients.

fmod <- lmer(c_value ~ timepoint * condition + (1|subject), data = model_data)

# extract model coefs
parameters::parameters(fmod) |>
 gt() |>
 fmt_number(decimal = 3) |>
 cols_hide(c("Effects", "Group", "CI", "df_error")) |>
 tab_header("Frequentist model results: CS activity")

Table 1: Frequentist model results: CS activity

| Parameter | Coefficient | SE | CI_low | CI_high | t | p |
| --- | --- | --- | --- | --- | --- | --- |
| (Intercept) | -0.957 | 3.713 | -8.430 | 6.516 | -0.258 | 0.798 |
| timepointw1 | -0.184 | 4.333 | -8.906 | 8.537 | -0.043 | 0.966 |
| timepointw2 | -7.348 | 4.333 | -16.070 | 1.373 | -1.696 | 0.097 |
| conditionacipimox | -0.883 | 5.569 | -12.092 | 10.327 | -0.158 | 0.875 |
| timepointw1:conditionacipimox | 9.876 | 6.499 | -3.206 | 22.959 | 1.520 | 0.135 |
| timepointw2:conditionacipimox | 16.180 | 6.499 | 3.098 | 29.263 | 2.490 | 0.016 |
| SD (Intercept) | 6.631 | 2.091 | 3.574 | 12.303 | NA | NA |
| SD (Observations) | 9.689 | 1.211 | 7.583 | 12.378 | NA | NA |

# bayesian model for comparison
parameters::parameters(rnd_int_mod) |>
 gt() |>
 fmt_number(decimal = 3) |>
 cols_hide(c("Component", "CI")) |>
 tab_header("Bayesian model results: CS activity")

Table 1: Bayesian model results: CS activity

| Parameter | Median | CI_low | CI_high | pd | Rhat | ESS |
| --- | --- | --- | --- | --- | --- | --- |
| b_Intercept | -1.036 | -8.699 | 6.581 | 0.608 | 1.000 | 6,016.164 |
| b_timepointw1 | 0.041 | -8.944 | 9.024 | 0.504 | 1.000 | 7,957.055 |
| b_timepointw2 | -7.181 | -16.145 | 1.865 | 0.944 | 1.000 | 7,427.161 |
| b_conditionacipimox | -0.676 | -11.878 | 10.682 | 0.547 | 1.000 | 6,050.306 |
| b_timepointw1:conditionacipimox | 9.519 | -4.072 | 22.913 | 0.919 | 1.000 | 7,213.620 |
| b_timepointw2:conditionacipimox | 15.798 | 2.198 | 28.757 | 0.988 | 1.001 | 7,205.233 |
| sigma | 10.092 | 7.951 | 13.211 | 1.000 | 1.001 | 3,633.210 |

The predictions from the frequentist model are plotted along with the data below.

# plot
plot(estimate_means(fmod)) +
 scale_colour_manual(values = clrs,
 name = "condition", labels = c("Placebo", "Acipimox")) +
 labs(title = "Frequentist model results: CS activity",
 x = "Timepoint", y = xlab)

We selected `at = c("timepoint", "condition")`.

| 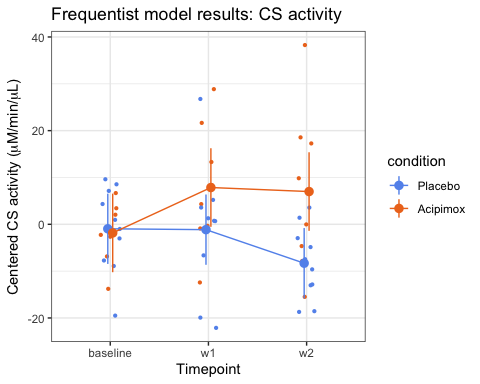  Figure 8: Frequentist model predictions (with data) |
| --- |

The frequentist and Bayesian models are generally in agreement in terms of model coefficients. Note that we are able to get a richer inference (e.g probability of direction) from the Bayesian model.

**Respirometry Bayesian analysis**

**Introduction**

This study examined the effects of acipimox, an NAD^+^ precursor on mitochondrial function. There were two treatment groups: placebo (n = 10) and acipimox (n = 8). Participants had muscle biopsies at baseline and at 1- & 2-weeks following placebo/acipimox treatment. High resolution respirometry was used to gather data on mitochondrial activity in skeletal muscle. The parameters examined were:

- Baseline - baseline measurement
- Li - uncoupled respiration associated with heat generation
- Pi - coupled respiration supported by complex I
- Pi+ii - coupled respiration supported by complex I + II
- E - maximal uncoupled respiration

In this analysis we will use a Bayesian hierarchical approach to model the average change in each respirometry variable over time in the placebo and acipimox groups. Only results for E are shown. A different variable can be selected in the *Statistical modeling* section below.

**Packages & environment**

First, we load the packages we will need and make sure we start with clean environment.

library(brms) # bayesian modeling
library(bayesplot) # diagnostic plots etc
library(tidyverse) # data wrangling
library(gridExtra) # multiplot figures
library(tidybayes) # plots; distributions
library(easystats) # model summaries
library(priorsense) # for prior robustness checking; remotes::install_github("n-kall/priorsense")
library(gt) # tables
library(lme4) # frequentist analysis
theme_set(theme_bw())

# start clean
rm(list = ls())

Next, we load the data. The data is in long format (each row represents one measure) & contains the following variables:

- subject - subject ID
- HRR_status - respirometry variable
- timepoint - the timepoint (baseline, w1 & w2)
- condition - the condition (placebo, acipimox)
- value - the value of the respirometry variable in arbitrary units (AU)

We first convert the subject, HRR_state, timepoint and condition variables to factors & reverse the order of the condition variable so placebo is the reference category for plotting and modeling.

hrr_data <- read_csv('data/HRR_long_data.csv')
# remove -ve values; impossible; prob miscalibarion
hrr_data <- hrr_data |>
 filter(value > 0)

# factors from subj, condition, timepoint
hrr_data <- hrr_data |>
 mutate(across(where(is.character), as.factor))
# reverse order of placebo and acipimox for plots & model etc
hrr_data <- hrr_data |>
 mutate(condition = fct_rev(condition))

**Exploratory plots**

Next, we create an exploratory plot.

# set plot colours
clrs <- c("cornflowerblue", "chocolate2")

# all proteins over intervention b -> w1 -> w2
hrr_data |>
 summarise(mn = mean(value), .by = timepoint:HRR_state) |>
 ggplot(aes(timepoint, mn, colour = condition)) +
 geom_point(position = position_jitterdodge(jitter.width = 0.05), size = 3) + # plot means
 geom_line(aes(group = condition), position = position_jitterdodge(jitter.width = 0.05)) + # addline
 geom_point(data = hrr_data, aes(timepoint, value, colour = condition),
 position = position_jitterdodge(jitter.width = 0.05),
 alpha = 0.4) + # add raw values
 # x labels
 scale_x_discrete(labels=c("baseline" = "B",
 "w1" = "W1",
 "w2" = "W2")) +
 # legend labels & plot colours
 scale_colour_manual(values = clrs,
 name = "Condition", labels = c("Placebo", "Acipimox")) +
 labs(x = 'Timepoint', y = 'AU') +
 facet_wrap(~HRR_state, scales = 'free_y')

ggsave(filename = 'plots/EDA_plot1.png')

Saving 5 x 4 in image

| 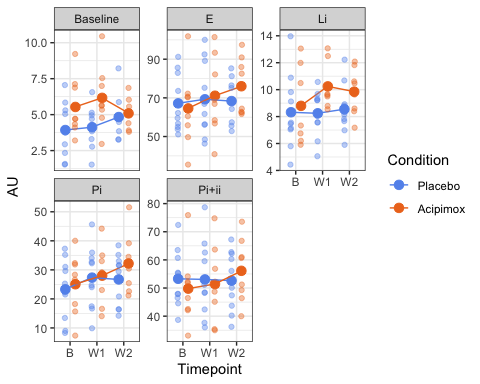  Figure 1: Exploratory figure |
| --- |

## Statistical modeling

We will build Bayesian hierarchical (mixed) models for each respirometry variable; this document shows results for E (maximal uncoupled respiration) only. The “random” term in these models will be the subject variable to account for repeated observations from each participant across the study timeline.

First, we select a respirometry variable to model and create some summary statistics. We also mean center the data as this makes the later specification of priors easier.

# select HRR_state here
# 'Baseline', 'Pi', 'Li', 'E', 'Pi+ii'
hoi <- 'E'
hoi_data <- hrr_data |>
 filter(HRR_state == hoi)

# get summary stats
summary_tab_nm <- paste("tables/", hoi, "_summary_data.docx", sep = "")
hoi_data |>
 summarise(Mean = mean(value), SD = sd(value), .by =c(condition, timepoint)) |>
 gt() |>
 fmt_number(decimals = 2) |>
 cols_align(columns = 'timepoint',
 align = c("left")) |>
 tab_footnote(paste("Condition: ", hoi, sep = "")) |>
 gtsave(summary_tab_nm)

# center data for ease of prior setting
model_data <- hoi_data |>
 mutate(c_value = value-mean(value))

**Setting priors**

The model we will estimate is:

$$y_{i}=\left( \beta_{0}+\sigma_{i} \right)+\beta_{1}\times time_{i}+\beta_{2}\times cond_{i}+\beta_{3}\times\left( time_{i}\times cond_{i} \right)+\epsilon_{i}$$

We need to set priors on each of the model parameters we are estimating. Here we estimate an overall intercept ($\beta_{0}$), a main effect of treatment ($\beta_{1}$), a main effect of time ($\beta_{2}$), the interaction between timepoint & condition ($\beta_{3}$), the variation in subject specific intercepts ($\sigma$) and the residual variation ($\epsilon$).

**Intercept prior**

The overall intercept is expected value when all other coefficients in a linear model are zero. In our context this is the mean of the placebo treatment at baseline. The data have been mean centered so we will set the prior mean at zero. With a lack of information on the centered values we would expect from respirometery data a sensible prior for the overall intercept would be $\sim N\left( 0, 4\times sd of the data \right)$. The mean is our most plausible value for the overall intercept but it is plausible the overall intercept could vary by the four times amount of variability we see in the data overall. Whilst this is wide from a biological perspective it provides much more regularisation than the flat prior implied by the usual frequentist tests.

# intercept priors
int_prior_sd <- model_data |>
 summarise(sds = ceiling(sd(c_value)*4)) |>
 pull()

**Slope and variance priors**

Next, we have to set priors for the $\beta$ (slope) coefficients in our model, the variance in the intercepts ($\sigma$) and the residual variance ($\epsilon$). For the slope coefficients we will use a student t prior with 3 degrees of freedom & a mean of zero (slopes could be positive or negative) because we expect the slopes could be positive or negative. The HRR_state variables are on different scales, so we set the prior dispersion using the observed standard deviation for each HRR_state multiplied by 4.

# slope sd
beta_prior_sd <- hoi_data |>
 summarise(sds = ceiling(sd(value)*4)) |>
 pull()

# sd prior
sd_prior <- model_data |>
 summarise(sds = ceiling(sd(c_value))*2) |>
 pull()

Below we define the prior for the $\beta$ coefficients in the model as $\sim t\left( 3,0,sd \right)$ where 3 is the “degrees of freedom” (dof) parameter, 0 is the mean and sd is the dispersion parameter as defined above.

We use the stanvar() function to make the user derived values available to the Stan language which will do the MCMC sampling for us via the brms package.

stanvars <- stanvar(int_prior_sd, name="int_prior_sd" ) +
 stanvar(beta_prior_sd, name = "beta_prior_sd") +
 stanvar(sd_prior, name = "sd_prior")

For the intercept variation and residual variation, we use the same $t$ distribution but we limit the plausible values to only positive values (lb = 0; lower bound = 0) because variances cannot be negative and set the dispersion parameter to be wide on the scale of all the data using the standard deviation of all the data. These variance priors suggest that the low variances are most likely but allow room for larger variance if the data suggests that.

rnd_int_priors <- c(prior(normal(0, int_prior_sd), class = Intercept), # intercept on mean of ALL data
 prior(student_t(3, 0, beta_prior_sd), class = b), # beta coef prior
 prior(student_t(3, 0, sd_prior), class = sd, lb = 0), # random intercept sd (sigma)
 prior(student_t(3, 0, sd_prior), class = sigma, lb = 0)) # residual variance (epsilon)

We can plot these priors to examine plausible values for each of the parameters we are trying to estimate.

# plot priors
rnd_int_priors |>
 parse_dist() |>
 ggplot(aes(y = paste(class, "~", format(.dist_obj)), xdist = .dist_obj)) +
 stat_halfeye(.width = 0.95,
 slab_linewidth = 0.5,
 slab_color = "black",
 slab_fill = "cadetblue") +
 labs(title = paste("Respirometry Priors for ", hoi, sep = " "),
 x = "AU",
 y = NULL)

| 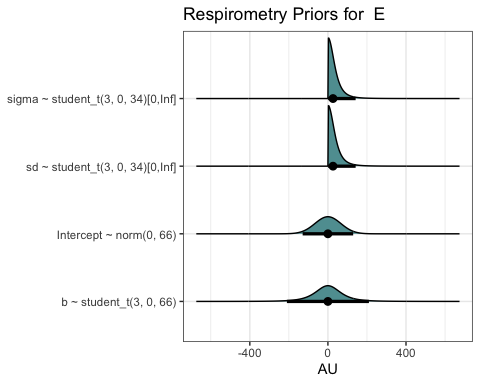  Figure 2: Priors used in the analysis |
| --- |

Next, we carry out prior predictive checks. Prior predictive checks involve simulating data from the model as defined by the priors *in the absence of data* and examining how compatible those simulations are with values we might expect. Here the priors have been chosen to be wide on the scale of the data and so our simulated data should also be more variable (wider) than the data we have but not wholly unreasonable.

# prior predictive check
prior_pred <- model_data |>
 brm(formula = c_value ~ timepoint * condition + (1|subject),
 prior = rnd_int_priors,
 family = gaussian(),
 cores = 4,
 iter = 2000,
 sample_prior = "only",
 stanvars=stanvars)

We examine the coefficients from the prior predictive model.

# check
parameters(prior_pred, effects = "fixed") |>
 gt() |>
 tab_header(title = paste(hoi, " Prior predicitve check", sep = "")) |>
 fmt_number(decimal = 3) |>
 cols_hide(c("Component", "CI")) |>
 tab_header("Prior predicitve model coefficients")

Table 1: Prior predicitve model coefficients

| Parameter | Median | CI_low | CI_high | pd | Rhat | ESS |
| --- | --- | --- | --- | --- | --- | --- |
| b_Intercept | 1.107 | -191.655 | 188.559 | 0.504 | 1.000 | 2,555.268 |
| b_timepointw1 | 0.065 | -206.183 | 225.744 | 0.500 | 1.001 | 2,427.578 |
| b_timepointw2 | 0.067 | -178.456 | 197.711 | 0.501 | 1.002 | 4,273.457 |
| b_conditionacipimox | -1.514 | -216.033 | 229.630 | 0.510 | 1.001 | 1,338.414 |
| b_timepointw1:conditionacipimox | -1.115 | -221.721 | 196.882 | 0.507 | 1.001 | 1,883.485 |
| b_timepointw2:conditionacipimox | 1.298 | -214.293 | 210.735 | 0.508 | 1.001 | 2,858.275 |
| sigma | 26.690 | 1.238 | 144.878 | 1.000 | 0.999 | 4,832.519 |

Next, we generate plots to examine how the MCMC sampling process for the model has converged and the distribution of the model predictions without any data input. First, we examine a histogram of the prior model predictions and MCMC traceplots.

# model sampling check
mcmc_hist(prior_pred, regex_pars = "^b_", pars = "sigma")

`stat_bin()` using `bins = 30`. Pick better value with `binwidth`.

mcmc_trace(prior_pred, regex_pars = "^b_", pars = "sigma")

| \| 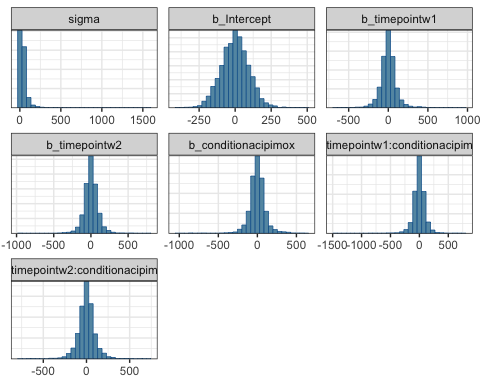  (a) Prior model predictions \| \| --- \| \| 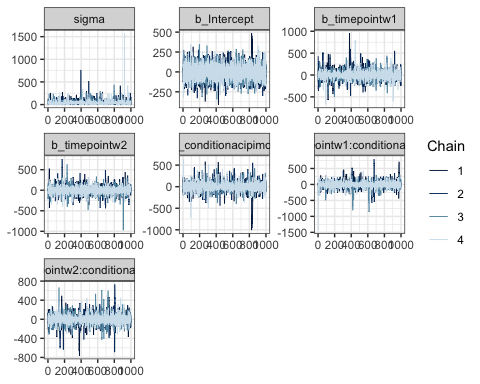  (b) Prior MCMC Traceplots \|   Figure 3: Prior convergence checks |
| --- | --- | --- |

The histogram suggests samples in line with our expectations and the MCMC traceplots look like “hairy catepillars” as we’d hope if the MCMC process is effectively sampling the parameter space.

Next, we examine the prior predictive density using 2000 draws from the prior predictive distribution. This plot shows estimates for respirometry signal from the model but in the absence of data.

Pp_check(prior_pred, ndraws = 2000, prefix = “ppd”,
 type = “stat”) +
 labs(title = paste(“Prior predictive distribution for “, hoi, sep = “”))

`stat_bin()` using `bins = 30`. Pick better value with `binwidth`.

| 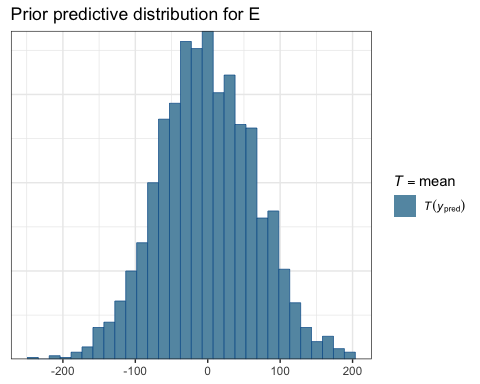  Figure 4: Prior predictive density |
| --- |

Overall, we are satisfied with these checks and proceed to modeling.

**Bayesian modeling**

In the code below we use the brms package to run the model with the priors and data defined above.

rnd_int_mod <- model_data |>
 brm(formula = c_value ~ timepoint * condition + (1|subject),
 prior = rnd_int_priors,
 cores = 4,
 chains = 4,
 iter = 5000,
 stanvars=stanvars,
 seed = 7)

**Prior robustness check**

It is important that we check the robustness of the model in the face changes to the prior. Kallioinen et al present a method for carrying this out by power scaling priors (see [here](http://arxiv.org/abs/2107.14054)). This approach is implemented in the priorsense package.

chk <- powerscale_sensitivity(rnd_int_mod)$sensitivity

Loading required namespace: testthat

chk[,1:4] |>
 gt() |>
 fmt_number(decimals = 2) |>
 tab_header(paste("Respirometry ", hoi, " Prior robustness check", sep = ""))

Table 1: Respirometry E Prior robustness check

| variable | prior | likelihood | diagnosis |
| --- | --- | --- | --- |
| b_Intercept | 0.00 | 0.06 | - |
| b_timepointw1 | 0.00 | 0.15 | - |
| b_timepointw2 | 0.00 | 0.16 | - |
| b_conditionacipimox | 0.01 | 0.05 | - |
| b_timepointw1:conditionacipimox | 0.01 | 0.15 | - |
| b_timepointw2:conditionacipimox | 0.01 | 0.15 | - |
| sd_subject__Intercept | 0.01 | 0.32 | - |
| sigma | 0.01 | 0.74 | - |
| Intercept | 0.00 | 0.03 | - |
| r_subject[RJ001,Intercept] | 0.00 | 0.08 | - |
| r_subject[RJ0010,Intercept] | 0.00 | 0.14 | - |
| r_subject[RJ0011,Intercept] | 0.00 | 0.21 | - |
| r_subject[RJ0012,Intercept] | 0.00 | 0.30 | - |
| r_subject[RJ0013,Intercept] | 0.00 | 0.28 | - |
| r_subject[RJ0014,Intercept] | 0.00 | 0.05 | - |
| r_subject[RJ0015,Intercept] | 0.00 | 0.24 | - |
| r_subject[RJ0016,Intercept] | 0.00 | 0.21 | - |
| r_subject[RJ0017,Intercept] | 0.00 | 0.04 | - |
| r_subject[RJ0018,Intercept] | 0.00 | 0.04 | - |
| r_subject[RJ0019,Intercept] | 0.00 | 0.32 | - |
| r_subject[RJ002,Intercept] | 0.00 | 0.06 | - |
| r_subject[RJ003,Intercept] | 0.00 | 0.05 | - |
| r_subject[RJ005,Intercept] | 0.00 | 0.23 | - |
| r_subject[RJ006,Intercept] | 0.00 | 0.19 | - |
| r_subject[RJ007,Intercept] | 0.00 | 0.18 | - |
| r_subject[RJ008,Intercept] | 0.00 | 0.14 | - |
| r_subject[RJ009,Intercept] | 0.00 | 0.06 | - |

The lack of any indicators (e.g. “prior-data conflict” or “weak likelihood”) in the diagnosis column of the chk object means we are happy that our prior is reasonably robust.

**Explore the model**

Next we generate some plots to check the results of our model. First, we examine the overall predictions MCMC traceplots as we did above for the prior predictive checks.

# model sampling check
mcmc_hist(rnd_int_mod, regex_pars = "^b_", pars = "sigma")

`stat_bin()` using `bins = 30`. Pick better value with `binwidth`.

mcmc_trace(rnd_int_mod, regex_pars = "^b_", pars = "sigma")

| \| 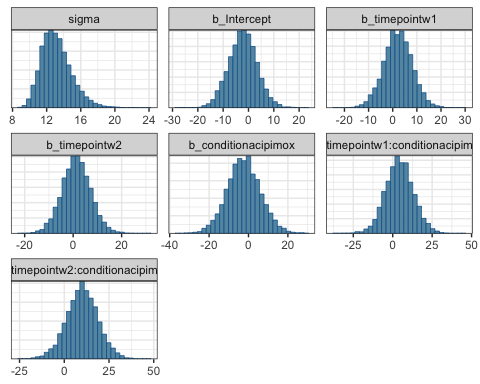  (a) Posterior model predictions \| \| --- \| | \| 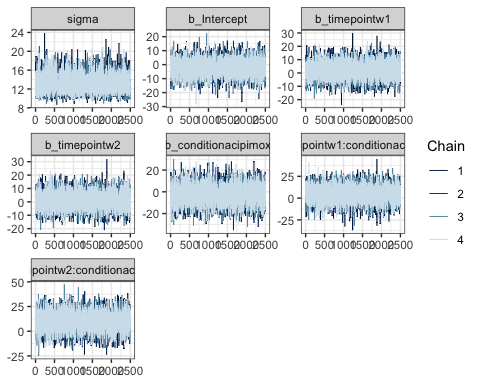  (b) Posterior MCMC Traceplots \| \| --- \| |
| --- | --- | --- | --- |

Figure 5: Prior convergence checks

These plots suggest the model has converged well.

Next, we carry out posterior predictive checks to examine how well the model has fit the data.

# get yrep data
yrep <- brms::posterior_predict(rnd_int_mod, ndraws = 5000)
# plots of posteriors vs predicted
# timepoint
bayesplot::ppc_stat_grouped(model_data$c_value, yrep, stat = "mean", group = model_data$timepoint)

`stat_bin()` using `bins = 30`. Pick better value with `binwidth`.

pp_check(rnd_int_mod, ndraws = 10, group = "timepoint", type = "dens_overlay_grouped")

# condition
bayesplot::ppc_stat_grouped(model_data$c_value, yrep, stat = "mean", group = model_data$condition)

`stat_bin()` using `bins = 30`. Pick better value with `binwidth`.

pp_check(rnd_int_mod, ndraws = 10, group = "condition", type = "dens_overlay_grouped")

| \| 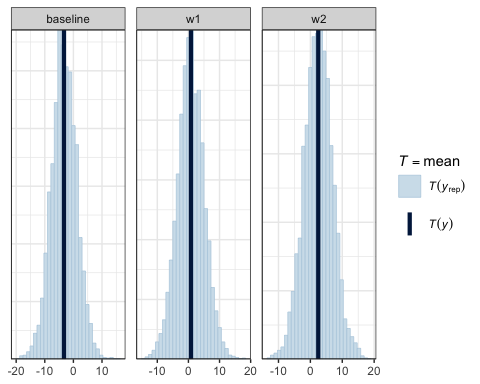  (a) Posterior predictions for timepoint - histogram \| \| --- \| | \| 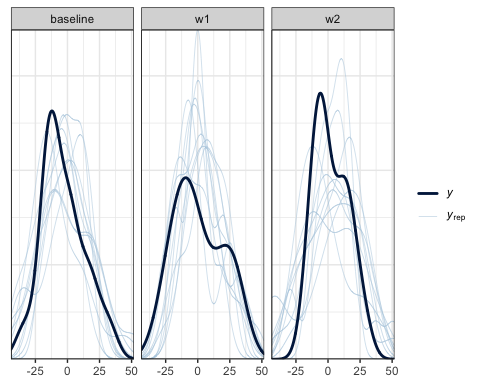  (b) Posterior predictions for timepoint - kernel density \| \| --- \| |
| --- | --- | --- | --- |

| \| 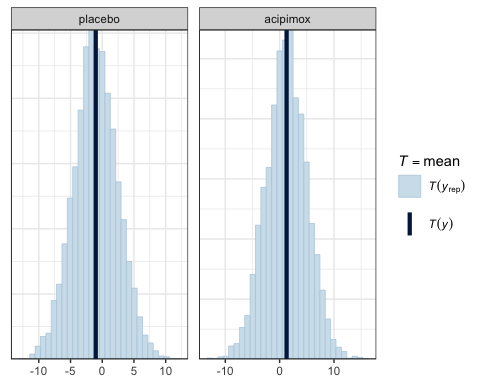  (c) Posterior predictions for condition - histogram \| \| --- \| | \| 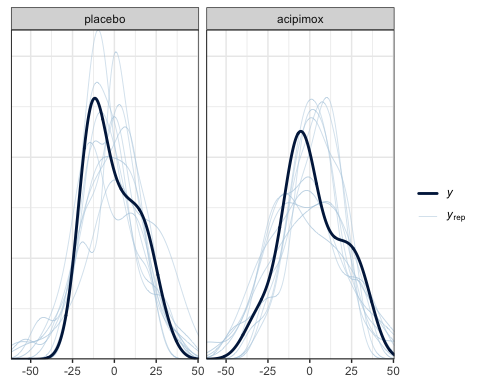  (d) Posterior predictions for condition - kernel density \| \| --- \| |
| --- | --- | --- | --- |

Figure 6: Posterior predictive check

These plots show the result of posterior predictive checking (PPC). The idea behind posterior predictive checking is simple: if a model is a good fit we should be able to use it to generate data that resemble the data that we observed (see [here](https://rss.onlinelibrary.wiley.com/doi/abs/10.1111/rssa.12378)). For both the main effects of timepoint and condition the histogram PPC plots show the mean of the data as a dark vertical line and the histogram shows the distribution of means we would predict from our model. In both cases the data mean lies in the middle of the posterior predictive distribution; this is exactly what we would expect. The density plots show different realisations of data according to the posterior distribution. The density plot for the actual data is shown as the darker line. These plots suggest reasonable posterior fit.

**Model parameters**

We can use packages from the [easystats](https://easystats.github.io/easystats/) meta-package to extract & tabulate the model coefficients.

# model parameters (easystats parameters pkg)
parameters(rnd_int_mod, effects = "fixed") |>
 gt() |>
 fmt_number(decimal = 3) |>
 cols_hide(c("Component", "CI")) |>
 tab_header(paste(hoi, " Model coefficients", sep = ""))

Table 1: E Model coefficients

| Parameter | Median | CI_low | CI_high | pd | Rhat | ESS |
| --- | --- | --- | --- | --- | --- | --- |
| b_Intercept | -2.117 | -13.202 | 8.877 | 0.652 | 1.000 | 4,829.772 |
| b_timepointw1 | 2.229 | -9.463 | 13.678 | 0.649 | 1.001 | 6,651.218 |
| b_timepointw2 | 1.278 | -10.344 | 12.976 | 0.587 | 1.001 | 6,918.067 |
| b_conditionacipimox | -2.463 | -19.035 | 14.357 | 0.618 | 1.000 | 4,498.272 |
| b_timepointw1:conditionacipimox | 4.187 | -12.887 | 21.797 | 0.689 | 1.001 | 5,745.180 |
| b_timepointw2:conditionacipimox | 10.089 | -7.888 | 27.358 | 0.877 | 1.000 | 6,003.214 |
| sigma | 12.950 | 10.286 | 17.199 | 1.000 | 1.000 | 4,491.050 |

The (Intercept) coefficient is the mean value at baseline in the placebo group; this has to be interpreted in the context of the mean centered data. The timepointw1 and timepointw2 coefficients are the changes from baseline in the placebo group at timepoints W1 and W2. The conditionacipimox coefficient is the difference between placebo and acipimox conditions at baseline. The timepointw1:conditionacipimox & timepointw2:conditionacipimox coefficients are the differences between baseline and W1 and W2 (respectively) in the acipimox condition. The Rhat & ESS are model diagnostics and suggest we have a model that has converged (Rhat) and a high number of posterior samples (ESS) (see [here](http://arxiv.org/abs/2011.01808)).

Instead of examining differences across conditions we can extract marginal means for each condition. These are the model estimated average values of the c_value variable at each condition.

estimate_means(rnd_int_mod, at = c( "condition", "timepoint"), test = NULL) |>
 gt() |>
 fmt_number(decimal = 3) |>
 tab_header(paste(hoi, " Marginal means", sep = ""))

Table 1: E Marginal means

| condition | timepoint | Mean | Median.1 | CI_low | CI_high |
| --- | --- | --- | --- | --- | --- |
| placebo | baseline | -2.117 | -2.117 | -13.202 | 8.877 |
| acipimox | baseline | -4.587 | -4.587 | -17.204 | 7.688 |
| placebo | w1 | -0.021 | -0.021 | -10.841 | 11.065 |
| acipimox | w1 | 1.940 | 1.940 | -10.773 | 14.443 |
| placebo | w2 | -0.882 | -0.882 | -11.987 | 10.128 |
| acipimox | w2 | 6.653 | 6.653 | -5.767 | 19.520 |

Perhaps most informatively we can extract contrasts for the difference between each timepoint in each condition.

estimate_contrasts(rnd_int_mod, contrast = "timepoint", at = "condition", test = "pd") |>
 gt() |>
 fmt_number(decimal = 3) |>
 tab_header(paste(hoi, " Condition contrasts", sep = ""))

Table 1: E Condition contrasts

| Level1 | Level2 | condition | Difference | CI_low | CI_high | pd |
| --- | --- | --- | --- | --- | --- | --- |
| baseline | w1 | placebo | -2.229 | -13.678 | 9.463 | 0.649 |
| baseline | w2 | placebo | -1.278 | -12.976 | 10.344 | 0.587 |
| w1 | w2 | placebo | 0.846 | -10.593 | 12.481 | 0.558 |
| baseline | w1 | acipimox | -6.576 | -19.508 | 6.587 | 0.842 |
| baseline | w2 | acipimox | -11.411 | -24.357 | 1.605 | 0.959 |
| w1 | w2 | acipimox | -4.861 | -17.842 | 8.316 | 0.775 |

This contrast table includes the “probability of direction” (pd column). This tells us the probability of the direction of a given contrast.

**Examining hypotheses**

We can also examine specific hypotheses by calculating area under the posterior distribution for a given hypothesis. Below we test hypotheses for baseline vs W1 and baseline vs W2 in the placebo and acipimox conditions being equal to zero.

# test hypotheses & get posterior draws for hypotheses
hyp <- c("timepointw1 = 0", "timepointw2 = 0",
 "timepointw1 + timepointw1:conditionacipimox = 0",
 "timepointw2 + timepointw2:conditionacipimox = 0")

# get hypothesis posterior draws
posts <- hypothesis(rnd_int_mod, hyp)$samples
# test direction of effect
hyp_tests <- bayestestR::describe_posterior(
 posts,
 test = c("p_direction"),
 centrality = "median")
hyp_tests$Parameter <- c("Placebo:T1-baseline",
 "Placebo:T2-baseline",
 "Acipimox:T1-baseline",
 "Acipimox:T2-baseline")
hyp_tests |>
 gt() |>
 fmt_number(decimal = 2) |>
 tab_header("Probability of direction for defined hypotheses")

Table 1: Probability of direction for defined hypotheses

| Parameter | Median | CI | CI_low | CI_high | pd |
| --- | --- | --- | --- | --- | --- |
| Placebo:T1-baseline | 2.23 | 0.95 | -9.46 | 13.68 | 0.65 |
| Placebo:T2-baseline | 1.28 | 0.95 | -10.34 | 12.98 | 0.59 |
| Acipimox:T1-baseline | 6.58 | 0.95 | -6.59 | 19.51 | 0.84 |
| Acipimox:T2-baseline | 11.41 | 0.95 | -1.60 | 24.36 | 0.96 |

We then extract the posterior data for these hypotheses and plot those to examine the probability of direction.

# plot
# hypothesis labels
ys <- c(bquote("T1-baseline"["plac"]),
 bquote("T2-baseline"["plac"]),
 bquote("T1-baseline"["aci"]),
 bquote("T2-baseline"["aci"]))

pl_ttl <- paste("Probability of Direction: ", hoi, sep = "")


# plot hypothesis posteriors i.e. prob dir; add prob info
plt_ttl <- paste("Probability of Direction: ", hoi, sep = "")
posts |>
 pivot_longer(cols = everything(), names_to = "cond", values_to = "draws") |>
 ggplot(aes(x = draws, y = rev(cond), fill = after_stat(x < 0))) +
 # see https://mjskay.github.io/ggdist/articles/slabinterval.html#highlighting-and-other-combinations
 # for colouring
 geom_vline(xintercept = 0, linetype = "dashed", linewidth = 1) +
 # useful for appearance of stat_halfeye
 stat_halfeye(.width = 0.95, slab_linewidth = 0.5, slab_color = "black") +
 scale_y_discrete(name = "",
 breaks = c("H1", "H2", "H3", "H4"),
 labels = rev(ys)) +
 scale_fill_manual(name = "Probability of direction: ", labels = c("Positive", "Negative"),
 values = clrs) +
 labs(title = plt_ttl,
 x = "Possible parameter values") +
 theme(legend.position = "bottom",
 axis.text.x = element_text(size=10),
 axis.text.y = element_text(size=12))

| 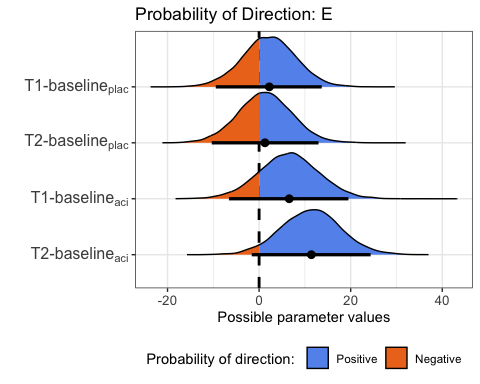  Figure 7: Probability of direction for study effects |
| --- |

**Frequentist Analysis**

We can also run a frequentist hierarchical model using the lme4 package to compare the model coefficients.

fmod <- lme4::lmer(c_value ~ timepoint * condition + (1|subject), data = model_data)

# extract model coefs
parameters::parameters(fmod) |>
 gt() |>
 fmt_number(decimal = 3) |>
 cols_hide(c("Effects", "Group", "CI", "df_error")) |>
 tab_header(paste(hoi, " Frequentist model results", sep = ""))

Table 1: E Frequentist model results

| Parameter | Coefficient | SE | CI_low | CI_high | t | p |
| --- | --- | --- | --- | --- | --- | --- |
| (Intercept) | -2.120 | 5.286 | -12.761 | 8.520 | -0.401 | 0.690 |
| timepointw1 | 2.108 | 5.613 | -9.190 | 13.406 | 0.376 | 0.709 |
| timepointw2 | 1.184 | 5.613 | -10.113 | 12.482 | 0.211 | 0.834 |
| conditionacipimox | -2.613 | 7.929 | -18.573 | 13.347 | -0.330 | 0.743 |
| timepointw1:conditionacipimox | 4.460 | 8.419 | -12.487 | 21.406 | 0.530 | 0.599 |
| timepointw2:conditionacipimox | 10.285 | 8.419 | -6.661 | 27.232 | 1.222 | 0.228 |
| SD (Intercept) | 11.041 | 2.855 | 6.652 | 18.328 | NA | NA |
| SD (Observations) | 12.550 | 1.569 | 9.823 | 16.035 | NA | NA |

# bayesian model for comparison
parameters::parameters(rnd_int_mod) |>
 gt() |>
 fmt_number(decimal = 3) |>
 cols_hide(c("Component", "CI")) |>
 tab_header(paste(hoi, " Bayesian model results", sep = ""))

Table 1: E Bayesian model results

| Parameter | Median | CI_low | CI_high | pd | Rhat | ESS |
| --- | --- | --- | --- | --- | --- | --- |
| b_Intercept | -2.117 | -13.202 | 8.877 | 0.652 | 1.000 | 4,829.772 |
| b_timepointw1 | 2.229 | -9.463 | 13.678 | 0.649 | 1.001 | 6,651.218 |
| b_timepointw2 | 1.278 | -10.344 | 12.976 | 0.587 | 1.001 | 6,918.067 |
| b_conditionacipimox | -2.463 | -19.035 | 14.357 | 0.618 | 1.000 | 4,498.272 |
| b_timepointw1:conditionacipimox | 4.187 | -12.887 | 21.797 | 0.689 | 1.001 | 5,745.180 |
| b_timepointw2:conditionacipimox | 10.089 | -7.888 | 27.358 | 0.877 | 1.000 | 6,003.214 |
| sigma | 12.950 | 10.286 | 17.199 | 1.000 | 1.000 | 4,491.050 |

The predictions from the frequentist model are plotted along with the data below.

# plot
plot(estimate_means(fmod)) +
 scale_colour_manual(values = clrs,
 name = "condition", labels = c("Placebo", "Acipimox")) +
 labs(title = paste("Respirometry Frequentist Model: ", hoi, sep = ""),
 x = "Timepoint", y = "AU")

We selected `at = c("timepoint", "condition")`.

| 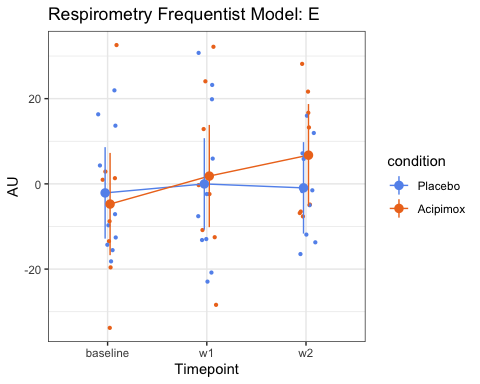  Figure 8: Frequentist model predictions (with data) |
| --- |

The frequentist and Bayesian models are in agreement in terms of model coefficients. Note that we are able to get a richer inference (e.g probability of direction) from the Bayesian model.
