# Supplementary material for: Nicotinic acid improves mitochondrial function and associated transcriptional pathways in older inactive males
Source: Transl Exerc Biomed. 2024 Nov 25;1(3-4):277–94. doi: 10.1515/teb-2024-0030 (PMC11653476; doi:10.1515/teb-2024-0030)
Supplement: Supplementary file 13 — Supplementary Material [file j_teb-2024-0030_suppl_013.docx]

**SUPPLEMENTAL METHODS**

**SPPBT**

The SPPBT was used to measure of lower extremity function of older adults and was performed similarly to previously described (1). The SPPBT involved three tests; balance, gait speed and chair rise, which were demonstrated to volunteers beforehand. Balance testing consisted of side by side, semi-tandem and tandem standing. Volunteers were asked to assume the correct foot positioning and when ready, the timer was initiated. Timing was ceased when volunteers moved their feet, grabbed surroundings for support or when 10 seconds had elapsed. Each volunteer began with the side by side stand, in which the heels are aligned. Those able to hold the side by side stand for 10 seconds then proceeded to the semi-tandem stand, in which the heel of one foot was placed to the side of the first toe of the contralateral foot (volunteers chose which foot to place forward). Those maintaining a semi-tandem stand for 10 seconds, continued to the full tandem stand, where the heel of one foot was placed directly in front of the toes of the contralateral foot. If the volunteer was unable to hold a balance for 10 seconds the rest of the balance challenges were terminated.

The gait speed test was a 2.44 m short walking course where volunteers were instructed to walk from the beginning of the course to the end at their normal walking speed, “as if they were walking to the shops”. After a visual demonstration by the researcher, volunteers performed the gait speed test 3 times. The average walking speed was calculated and used against the marking criteria.

Chair rise was tested by placing a chair against the wall and asking the volunteers to sit in the chair with their arms across their chest and rise to a full stand and then fully sit down. If successful, volunteers were asked to stand up and sit down 5 times, as quickly as they could.

Each test (i.e. balance, gait speed and chair rise) was given a score out of four and an overall score out of 12 was given, in line with originally described (1).

**Wingate**

Anaerobic power (mean, peak and lowest power) was determined by the Wingate anaerobic cycle test (Monarch 894E, Varberg, Sweden), which was modified to 10 seconds (as opposed to 30 seconds) to ensure the safety of the older volunteers. Volunteers warmed-up for 3 minutes by cycling at 60 rpm against minimal resistance. At the end of the warm-up, volunteers sprint cycled against a resistance of 7.5% of body weight for 10 seconds, remaining in the seated position throughout. Upon completion of the test, volunteers cooled down by cycling against minimal resistance for 1 minute, and thereafter volunteers were seated in a chair to rest for 10 minutes, allowing heart rate to return to baseline. Volunteers were monitored by 12-lead ECG throughout, which was supervised by a medic to monitor signs of cardiovascular abnormalities.

**Moderate and ramp incremental cycling**

Volunteers performed moderate intensity cycling exercise to obtain V_O2_ dynamics and the O_2_ cost, followed by incremental cycling exercise to exhaustion to obtain maximal measures of cardiorespiratory fitness *(VO_2peak_)* on an electronically-braked cycle ergometer (Lode Excalibur, Groningen, The Netherlands) (Figure S1). The same saddle and handlebar height configuration were used on all subsequent study visit after familiarisation and volunteers were asked to cycle at 70 prm throughout the both cycling tests. Volunteers were monitored by 12-lead ECG throughout, which was supervised by a medic to monitor signs of cardiovascular abnormalities.

**Moderate-intensity ‘step’ test**

On each experimental visit (i.e. V1, 2 and 3), volunteers completed a single moderate-intensity ‘step’ exercise bout to assess exercise economy (i.e. the steady-state oxygen (O_2_) cost of submaximal exercise) (2). This test consisted of 4 minutes of cycling against 20 W followed immediately by 6 minutes at a work rate that would require 80% of the GET (as determined on the pre-experimental visit (3)). During the exercise test, pulmonary gas exchange and ventilation were measured breath-by-breath using a respiratory gas analysis system (Ultima Series, Medgraphics, Minneapolis, USA). This system was calibrated before each test as per the manufacturer’s instructions. After the breath-by-breath data were averaged over 10-second periods, the O_2_ cost of exercise was determined as the mean O_2_ uptake ($\dot{V}$O_2_) over the final 60 seconds of exercise. The mean expired carbon dioxide ($\dot{V}C$O_2_), respiratory exchange ratio (RER) and minute ventilation ($\dot{V}$_E_) over the final 60 seconds of exercise were also determined.

**Ramp incremental exercise test**

On the familiarisation visit and each experimental visit, volunteers completed a ramp incremental exercise test to the limit of tolerance for the determination of the peak oxygen uptake ($\dot{V}$O_2peak_) and peak aerobic power output (PPO). After 4 minutes of cycling at 20 W the power output was increased linearly by 15 W/min until volitional exhaustion. The test was terminated when cadence fell by >10 rpm below 70 rpm. Pulmonary gas exchange and ventilation were measured throughout the test, as described above (see Moderate-intensity ‘step’ test). The $\dot{V}$O_2peak_, as well as the peak $\dot{V}C$O_2_ ($\dot{V}C$O_2peak_), RER (RER_peak_) and $\dot{V}$_E_ ($\dot{V}$_Epeak)_ were determined as the highest 30-s mean values attained before volitional exhaustion. The PPO was determined as the power output at the point of test termination.

**Physical activity levels**

Daily step count was measured using FitBit One (FitBit Inc, San Francisco, USA) throughout the duration of the study. Volunteers were instructed to wear their FitBit at hip height and to keep the FitBit on at all times except during showering/bathing and sleeping. All volunteers had at least 6 days of data that was included in analysis. Step count data from experimental visits was not included in statistical analysis.

**References**

1. Guralnik JM, Simonsick EM, Ferrucci L, Glynn RJ, Berkman LF, Blazer DG, Scherr PA, Wallace RB. A short physical performance battery assessing lower extremity function: Association with self-reported disability and prediction of mortality and nursing home admission. Journals Gerontol. 1994;49:85–94.

2. Vanhatalo A, Bailey SJ, Blackwell JR, DiMenna FJ, Pavey TG, Wilkerson DP, Benjamin N, Winyard PG, Jones AM. Acute and chronic effects of dietary nitrate supplementation on blood pressure and the physiological responses to moderate-intensity and incremental exercise. Am J Physiol - Regul Integr Comp Physiol. 2010;299:1121–31.

3. Beaver WL, Wasserman K, Whipp BJ. A new method for detecting anaerobic threshold by gas exchange. J Appl Physiol. 2016;121:2020–7.
